# Sample-efficient generative molecular design using memory manipulation

**DOI:** 10.1038/s42256-026-01200-4

**Published:** 2026-03-17

**Authors:** Jeff Guo, Junwu Chen, Anthony GX-Chen, Philippe Schwaller

**Affiliations:** 1 École Polytechnique Fédérale de Lausanne (EPFL), Lausanne, Switzerland; 2 National Centre of Competence in Research (NCCR) Catalysis, Lausanne, Switzerland; 3 New York University, New York, NY USA

**Keywords:** Drug discovery, Cheminformatics

## Abstract

Generative molecular design for drug discovery has recently achieved a wave of experimental validation. Language models operating on string-based representations of molecules are amongst the most successful architectures. The most important factor for downstream success is whether an in silico oracle (computational predictor of a molecule property) is well correlated with the desired end point (such as binding affinity). To this end, current methods use cheaper proxy oracles with a higher throughput before evaluating the most promising subset with high-fidelity oracles. The ability to directly generate molecules with optimal properties as predicted by high-fidelity oracles (computationally expensive simulations with greater predictive accuracy) could greatly enhance generative design and improve hit rates. However, current models are not efficient enough to consider such a prospect, exemplifying the sample efficiency problem. Recently, the Mamba architecture has been proposed as an alternative to transformers, which are widely used in large language models. Existing works have validated Mamba’s performance on tasks spanning natural language completion to biology foundation models. In this work, we introduce a framework called Saturn, which demonstrates the application of the Mamba architecture for generative molecular design. Here we elucidate how experience replay with data augmentation improves the sample efficiency and how Mamba intensifies the effect of this mechanism. Next, we show that Mamba with experience replay outperforms 16 models on multiparameter optimization tasks relevant to drug discovery and possesses sufficient sample efficiency to directly optimize density functional theory simulations as a high-fidelity oracle.

## Abstract (web summary)

Guo et al. train a Mamba-based language model for molecule generation and find that data augmentation and experience replay can enable the efficient generation of property-optimized small molecules.

## Main

In the last year and a half, there has been a surge of studies reporting the experimental validation of generative molecular design for drug discovery^1^. The most widely represented paradigm is algorithmic optimization, which proceeds via the following workflow: generate molecules, assess desirability using in silico oracles (computational predictor of molecule properties), update the model and repeat. When assessing the suitability of molecules without experimental validation, the crucial indicator of success is the correlation of an in silico oracle to the actual end point. Typically, the design follows a funnel workflow in which less resource-intensive oracles are initially used to prioritize the most promising subset for evaluation with computationally expensive high-fidelity oracles. A ubiquitous example is designing molecules with high binding affinity to a protein target, as estimated by molecular docking^2–5^. A tuned molecular docking protocol can offer some predictive capability^2,6^ and has been a common component in generative design workflows achieving experimental validation^1^. Subsequently, the most promising candidates from docking are subjected to higher-fidelity oracles, particularly molecular dynamics simulations, which can offer a much more accurate estimation of binding affinity^7^. Directly optimizing high-fidelity oracles offers the potential to greatly improve the quality of the generated set^8^. However, doing so is impractical due to computational cost, exemplifying the sample efficiency problem. Either simulation protocols become much faster without sacrificing accuracy, or generative models become sufficiently efficient to optimize under an acceptable oracle budget. We note that quantitative structure–activity relationship models are often used, which can have great predictive accuracy, but may suffer from a narrow domain of applicability (within their training data)^9^.

Existing works have tackled generative design using a variety of architectures, including simplified molecular-input line-entry systems (SMILES)^10^-based recurrent neural networks (RNNs)^11–15^, transformers^16–20^, variational autoencoders^21–24^, adversarial approaches^25,26^, graph-based models^3,26–28^, GFlowNets^29^, genetic algorithms (GAs)^5,30^, and diffusion or flow-based models^4,31–33^. Long short-term memory RNNs^11^ have also been used as a predictive model within a GA workflow to generate self-assembling peptides, thereby serving as a fitness function^34^.

Despite the recent trend towards three-dimensional molecular generation^31–33^, language-based models are amongst the most sample efficient^35–37^ and can satisfy three-dimensional-dependent objectives such as docking. Accordingly, text is still one of the most widely used molecular representations^10,38^ for generative design, with recent model architectures expanding beyond RNNs and transformers to structured state space sequence models^39^. The non-injective syntax of SMILES can confer advantageous properties for generative design. Specifically, a single molecule can be expressed as at least *N* (number of heavy atoms) SMILES, in a process known as SMILES augmentation, enumeration or randomization^40^. This mechanism can be exploited to pretrain models under low-data regimes^41–43^, improve sample efficiency^44,45^ and perform transfer learning with a single positive example^46^.

**Fig. 1:**
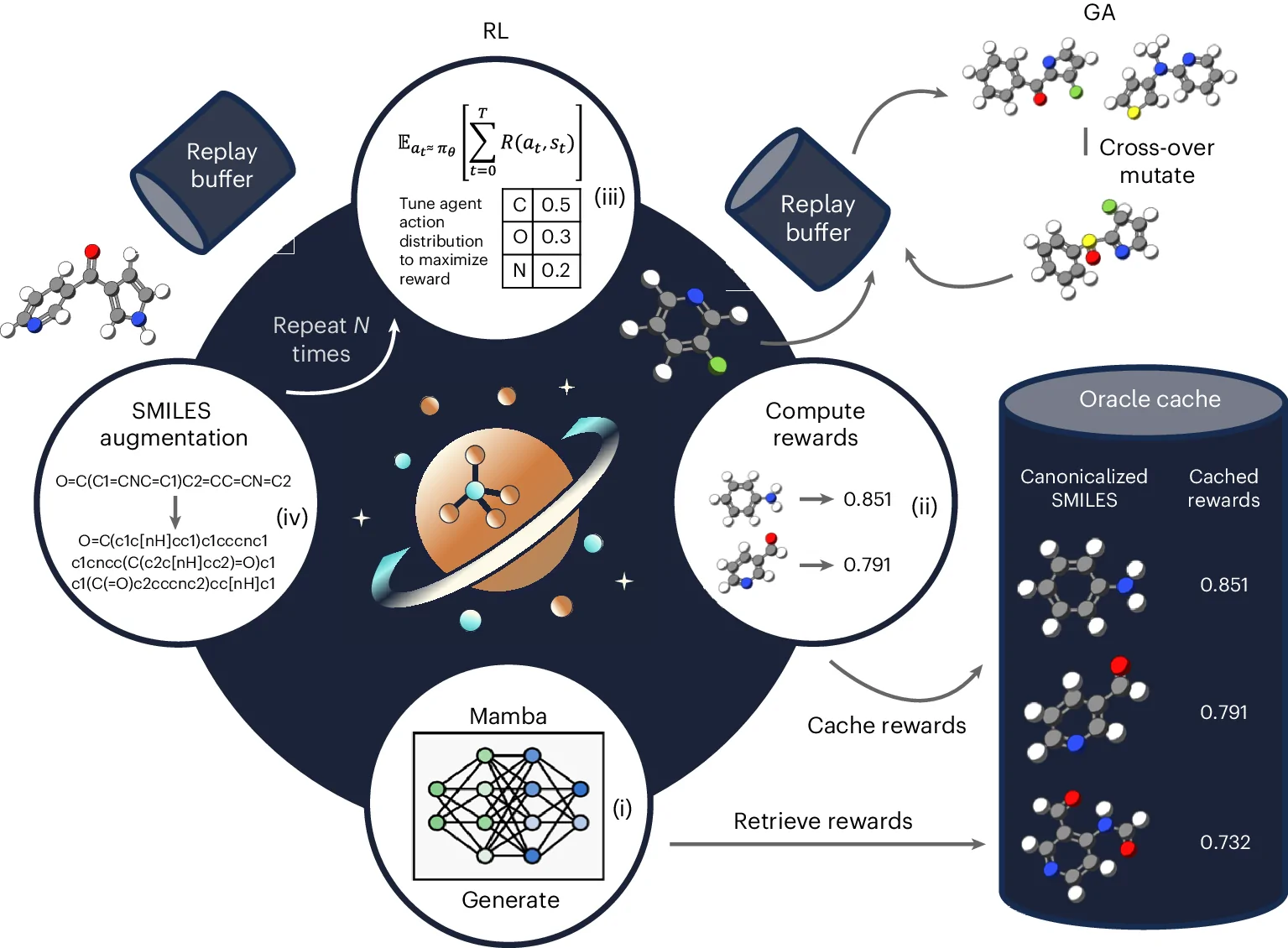
Saturn generative framework using the Mamba architecture. All generated SMILES and their rewards are stored in the oracle cache after canonicalization. A GA can be optionally applied using the replay buffer as the parent population. Augmented Memory is used to update the agent numerous times.

The release of the practical molecular optimization benchmark^35^ highlighted that improvements in sample efficiency is practically important. Accordingly, more recent studies^3–5,45,47^ have enforced fixed oracle budgets compared with other methods, with language-based models still amongst the best performing ones. More recently, Guo et al. proposed Augmented Memory, which combines experience replay^48^ with SMILES augmentation^40^ to improve sample efficiency. In this work, we release Saturn as a framework for generative molecular design. First, we elucidate the mechanism of Augmented Memory as the original work only showed its empirical benefits. Next, we systematically assess language-based architectures including RNNs^11^, decoder transformers^16,17^ and the recent Mamba^49^ model. Our results show that Mamba intensifies the effect of data augmentation and experience replay to improve sample efficiency by strategic overfitting. Our contributions are as follows:

- We elucidate the mechanism into how data augmentation, experience replay, and reinforcement learning improves sample efficiency.
- We comprehensively evaluate language model backbones including RNN, decoder transformer and Mamba, which enables us to characterize model-intrinsic and scaling properties that lead to improved sample efficiency.
- We propose a molecular generation framework called Saturn (Fig. 1), and show that the use of the Mamba architecture with the Augmented Memory^45^ optimization algorithm outperforms 15 models on multiparameter optimization (MPO) drug discovery tasks under heavily constrained oracle budgets.
- Using the same setup, we demonstrate the direct optimization of electronic properties with full geometry optimization at the density functional theory (DFT) fidelity level.

## Results

This section comprises three parts: understanding the optimization dynamics of Augmented Memory^45^ using a toy MPO task, benchmarking against 15 models (including dataset-screening baselines) on MPO docking tasks and directly optimizing for DFT electronic properties as a high-fidelity oracle. Every experiment was run across ten seeds (0–9 inclusive) excluding the DFT experiments due to high computational costs (three seeds, 0–2 inclusive instead). The oracle budget (number of unique molecules assessed) is varied across experiments to enable a fair comparison with previous works and will be clearly stated.

### Part 1: elucidating the optimization dynamics of Augmented Memory

We begin by identifying the optimal architecture and hyperparameters of Augmented Memory^45^. First, we experiment with varying the batch size and augmentation rounds of the Augmented Memory algorithm across the RNN^11^, decoder transformer^16,17^ and Mamba^49^ backbones. Next, we illustrate the sample efficiency–diversity trade-off and demonstrate that local exploitation in the chemical space can be a vital mechanism for sample efficiency.

Experimental details: we define a toy experiment with the following MPO objective: molecular weight of <350 Da; number of rings, ≥2; and maximize topological polar surface area^47^. Optimizing this objective requires generating molecules with rings saturated with heteroatoms, which are dissimilar from the training data. Hence, it is also testing out-of-support optimization. The oracle budget is 1,000 and the models were pretrained with ChEMBL 33 (ref. ^50^; Supplementary Section 1.6).

Metrics: the sample efficiency metrics are yield and oracle burden (OB). Yield is the number of unique generated molecules above a reward threshold, and OB is the number of oracle calls required to generate *N* unique molecules above a reward threshold. The reward threshold in this experiment is 0.7 as molecules start to possess saturated heteroatom rings. Most configurations generate at least some molecules passing this threshold within the budget, enabling us to report statistics.

Understanding the limits of Augmented Memory across backbones: Augmented Memory^45^ is a reinforcement learning (RL) algorithm that improves the sample efficiency by repeated learning from high-reward SMILES. We hypothesized that as long as unique high-reward SMILES are still generated, sample efficiency can improve with decreasing batch size, at the expense of diversity. Here repeat-generated SMILES do not impose additional oracle evaluations as we retrieve their rewards from a cache using SMILES canonicalization matching. We perform a grid search and vary the batch size (64, 32, 16 and 8) and augmentation rounds (0–20) across architecture backbones with similar parameter counts: RNN^11^ (5.8M), decoder transformer^16,17^ (6.3M) and Mamba^49^ (5.2M) (Supplementary Section 2 provides comprehensive results). Extended Data Table 1 contrasts the performance across the backbones given this hyperparameter range: increasing augmentation rounds improves the sample efficiency metrics (yield and OB). However, this trend is more consistent for Mamba in which the improvements are generally monotonic. In the most challenging OB 100 metric, Mamba is successful in 10/10 replicates, compared with only 5/10 and 4/10 for RNN and transformer, respectively (Extended Data Table 1). Given Mamba’s improved sample efficiency, we focus our analysis on comparing it with the RNN baseline in the remainder of this section (decoder transformer results are provided in Supplementary Section 2.2).

**Fig. 2:**
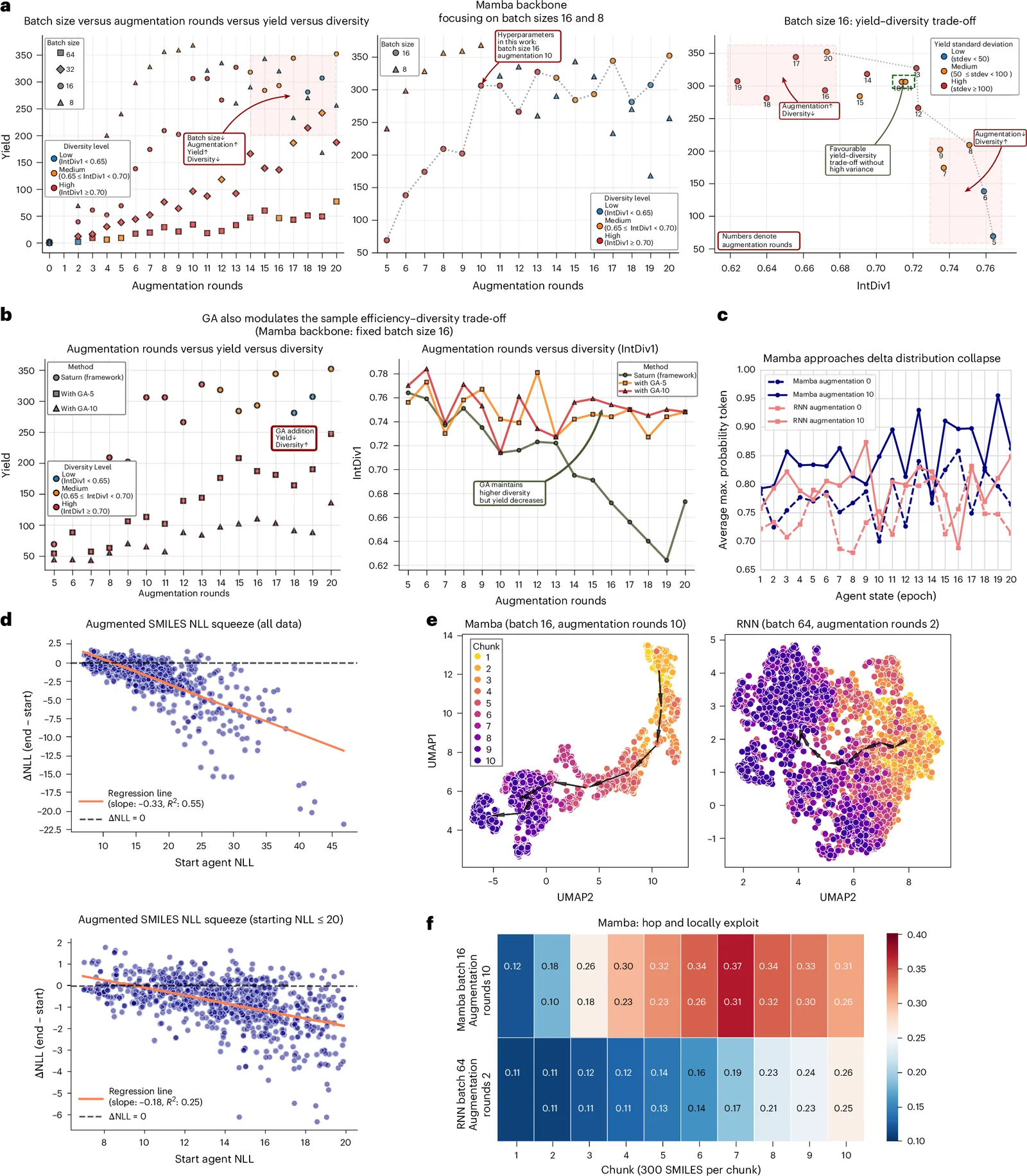
Modulating the exploration-exploitation trade-off and the mechanism of Augmented Memory. **a**, How batch size and augmentation rounds affect the yield and IntDiv1 (diversity). Generally, decreasing the batch size and increasing the augmentation rounds improve the yield at the expense of diversity. stdev, standard deviation. **b**, Fixing the batch size to 16, activating the GA maintains diversity but yield improves to a much lesser degree with increasing augmentation rounds. **c**, Average maximum token probability across RL epochs. Augmentation pushes the agent action distribution towards a delta distribution (putting most of the probability mass on specific tokens). **d**, Top: after running Augmented Memory, augmented forms of the buffer SMILES become more likely to be generated. The regression line measures the ΔNLL shift as the starting NLL varies and conveys that the larger the starting NLL (less likely), the larger the likelihood increase. Bottom: focusing on the SMILES forms that have a starting NLL ≤ 20, this relationship is lessened but the observation holds. The coefficient of determination (*R*^2^) is annotated in both subplots. **e**, 3,000 oracle budget test experiments chunked into 300 SMILES. Uniform manifold approximation and projection embedding of the agent chemical space traversal (arrows are the centroid of each chunk). Mamba exhibits a directional traversal as RNN (baseline Augmented Memory) continues to sample globally. **f**, Mamba exhibits a ‘hop and locally exploit’ behaviour in which the intrachunk Tanimoto similarity (top values) are higher than RNN. The bottom value is the interchunk similarity. Source data

Sample efficiency–diversity trade-off: we fix the Mamba backbone to better understand the interplay between batch size, augmentation rounds, yield and diversity. Figure 2a (left) shows that increasing augmentation rounds generally increases yield, but is inconsistent at low batch sizes (8). This is expected since the approximation to the expected reward (equation (3)) becomes more noisy. Yield improvements are more notable at lower batch sizes (16 and 8) with increasing augmentation rounds but comes at the expense of diversity (Fig. 2a (middle)). In this plot, batch size 8 with high augmentation rounds leads to high variance and a sharper diversity trade-off. Focusing on batch size 16, Fig. 2a (right) illustrates the efficiency–diversity Pareto front. The default hyperparameters in this work (batch size 16 and 10 augmentation rounds) balance sample efficiency and diversity and bounding variance. Taken together, these results corroborate the efficiency–diversity trade-off noted in previous works^35^, but goes further to explicitly show how this can be modulated by the batch size and augmentation rounds. Next, we posit that adding a GA, that is, GraphGA^30^, as part of the generative process can better maintain diversity, but we were interested in its effect on sample efficiency. Fixing the batch size to 16 (still using Mamba), we ran another grid search varying the augmentation rounds but now also with the GA activated: at every generation epoch, the GA was applied to the replay buffer, and five or ten resulting molecules were randomly selected and added to the sampled batch for RL. Note that since the reward function needs to be evaluated for these GA-derived molecules, this entails allocating a portion of the oracle budget to the GA. There are three main observations from Fig. 2b (left). (1) The results reaffirm that increasing the augmentation rounds generally increases yield, at the expense of diversity. (2) With the GA activated, the yield improves to a lesser degree but diversity is maintained. (3) Allocating more of the oracle budget to the GA (Saturn-GA-10) leads to a lower yield. Next, focusing on illustrating the effect of the GA on diversity, Fig. 2b (right) shows that activating the GA maintains diversity even with increasing augmentation rounds.

Considering the results from this section, it is clear that lower batch sizes and higher augmentation rounds with SMILES augmentation together with the GA are tools that modulate the efficiency–diversity trade-off. Practically, this implies that in situations where generating as many optimal solutions as possible is important, lower batch sizes and higher augmentation rounds can be advantageous. Conversely, if solutions should be more diverse, higher batch sizes, lower augmentation rounds and GA may be better suited. In the remainder of the main experiments, we do not use the GA (Supplementary Section 2.4 provides more GA results). Thus far, the results validate the efficiency–diversity trade-off, but why does Mamba outperform RNN?

Higher model capacity can benefit sample efficiency: Mamba (5.2M) and RNN (5.8M) have similar parameter counts, but during pretraining, the former converges to a lower loss (Supplementary Section 1.6). Mamba generates repeated SMILES (Extended Data Table 1), suggesting it overfits the data distribution. Figure 2c shows that with high augmentation rounds, the average maximum conditional token probability during generation approaches 1 (less so for RNN). This makes it likely—but not deterministic—to generate the same SMILES repeatedly. The results suggest that increased capability to model distributions can improve the sample efficiency, which naturally raises the question: can RNN (and decoder transformer) also achieve this? From a modelling perspective, one would expect that increasing the parameter count will enhance distribution matching. We verify this in Supplementary Section 5.7 and show that larger RNN and decoder transformers (>20M) can match the performance of Mamba (5.2M). Thus, these results affirm that enhanced distribution matching is not unique to Mamba. We use Mamba as the default architecture solely because it is more parameter efficient in our experiments. We next set out to better understand what exactly Augmented Memory is doing.

Squeezing the likelihood of augmented SMILES: although the original Augmented Memory work^45^ demonstrated its empirical benefits, we elucidate the underlying mechanism. To isolate its effect, we design a subexperiment as follows: generate molecules until the buffer is full (100) and then save the agent checkpoints before and after executing Augmented Memory (10 augmentation rounds) and save every augmented SMILES form (total, 1,000 SMILES). After execution, the (end) agent becomes more likely to generate the set of augmented SMILES (Fig. 2d, top). The more improbable the SMILES (high negative log-likelihood (NLL)), the larger its likelihood increases after Augmented Memory, that is, the ΔNLL shift is larger. Next, probable SMILES already exhibit a smaller ΔNLL shift because the softmax function saturates, causing minimal changes to the softmax output when the logits are tuned (Fig. 2d, bottom). Despite this, there is still a clear negative slope observed. Focusing on Fig. 2d (bottom), there are cases in which ΔNLL is positive after Augmented Memory, that is, it actually becomes less likely to generate the specific SMILES sequence. This observation is explained by the loss function (equation (5)), which is defined as the squared difference between the Augmented and Agent Likelihoods, and can be zero for some values. In this scenario, ΔNLL is dictated by the other SMILES in the batch. Conversely, as long as these likelihoods remain different, the loss function can push up the likelihood of all augmented SMILES. Therefore, Augmented Memory squeezes the likelihoods of generating augmented SMILES sequences towards the most likely one.

Mamba—hop and locally exploit: in the previous section, we showed that the agent becomes increasingly likely to generate the buffer molecules, as summarized by the regression line in Fig. 2d. We hypothesized that Mamba exhibits a hop and locally exploit behaviour. Because it may generate some SMILES representation of these molecules (strategic overfitting), small changes to any tokens in these augmented sequences equates to small changes to the same molecular graph, essentially performing a local exploitation (similar molecules, on average, exhibit similar properties). We verify our hypothesis with the following experiment: generate 3,000 molecules and separate the generated set into ten chunks (300 SMILES each). We trace the generated trajectory using uniform manifold approximation and projection^51^ and plot the chunk centroids, comparing Mamba and the baseline (vanilla Augmented Memory) (Fig. 2e). Mamba traverses the chemical space in an increasingly directional manner and the chunks are more locally confined. Further analysis into the intra- and interchunk Tanimoto similarity reveals that within chunks (top value), Mamba exhibits greater similarity than the baseline, and similarity is always lower between chunks (bottom values) (Fig. 2f). Taking these observations together, Mamba (batch size 16) with Augmented Memory (10 augmentation rounds) improves the sample efficiency via the hop and locally exploit behaviour. From here on, this model configuration will be referred to as Saturn and hyperparameters are fixed such that all performance metrics in the following sections are out of the box.

### Part 2: benchmarking the Saturn framework and demonstrating enhanced optimization

In this section, we follow Lee et al.^5^ (goal-aware fragment extraction, assembly and modification (GEAM) model) and benchmark the Saturn framework on MPO docking tasks.

Experimental details: for an exact comparison with GEAM^5^, we extracted their oracle code for our experiments and pretrained on the provided ZINC 250k (ref. ^52^) dataset (Supplementary Section 5.1). We note that the other model baselines were adapted from ref. ^5^ and these model checkpoints were also trained on the same ZINC 250k dataset. All models were tasked to optimize GEAM’s MPO objective function (equation (1)) for an exact comparison:

1 $$R(x)=\widehat{\mathrm{DS}}(x)\times \mathrm{QED}(x)\times \widehat{\mathrm{SA}}(x)\in [0,1],$$

where $\widehat{\mathrm{DS}}$ is the normalized QuickVina 2 (ref. ^53^) docking score and $\widehat{\mathrm{SA}}$ is the normalized synthetic accessibility score^54^ (Supplementary Section 5.3 provides the normalization details). Following GEAM^5^, docking was performed against five targets: parp1, fa7, 5ht1b, braf and jak2. We reproduced GEAM’s results and ran Saturn across ten seeds (0–9 inclusive) with an oracle budget of 3,000. We note that GEAM’s pretraining requires the labelled ZINC 250k dataset with all docking values already precomputed; therefore, there is a large upfront oracle cost (250k oracle calls, across five targets, equating to 1.25 million docking simulations).

Metrics: following Lee et al.^4,5^, we assess the hit ratio (%) (molecules with a better docking score than the median of known actives, the quantitative estimate of drug-likeness^55^ (QED) > 0.5, SA < 5) and novel hit ratio (%) (with the additional constraint of maximum Tanimoto similarity of 0.4 to the training data). We further propose strict hit ratio (%) and strict novel hit ratio (%), which filter for the more stringent criteria of QED > 0.7 (based on the DrugStore dataset of marketed drugs^55^) and SA < 3 (based on off-the-shelf catalogue molecules^54^). Although drug candidates need not necessarily meet these stricter thresholds, this metric that assesses optimization capability, which becomes pertinent when jointly optimizing all components, is especially crucial. From an optimization perspective, the objective function (equation (1)) aims to maximize QED and minimize SA and docking score simultaneously. Therefore, achieving high QED and low SA is part of the goal itself. We additionally measure molecular diversity using IntDiv1 (ref. ^56^) and #Circles^57^ with a distance threshold of 0.75.

The Saturn framework and GEAM outperform all baselines. We evaluate the hit ratio and include the random sampling of 3,000 molecules from ZINC 250k (ref. ^52^) and ChEMBL 33 (ref. ^50^) as the baselines (Table 1). The results show that only Augmented Memory^45^, Molecular Out-Of-distribution Diffusion (MOOD)^4^, GEAM^5^ and Saturn outperform these baselines, with GEAM and Saturn displaying similar performance. For the novel hit ratio (Extended Data Table 2), GEAM outperforms Saturn, which generates molecules more similar to the ZINC 250k pretraining dataset. To demonstrate how to satisfy this ‘novel‘ metric, we divide the task into two phases, akin to curriculum learning^58^. First, we task the base Saturn framework to generate molecules with a high Tanimoto dissimilarity (this is, the only optimization objective) to ZINC 250k (1,500 oracle calls). This new model checkpoint (Saturn–Tanimoto) is the new starting point for GEAM’s MPO task. Extended Data Table 2 shows that the performance immediately recovers and matches GEAM. We believe this is still a fair assessment as computing the Tanimoto similarity is inexpensive (this process took minutes) and also shows the flexibility of the framework.

**Table 1:** Hit ratio (%)

Method	Target protein				
	parp1	fa7	5ht1b	braf	jak2
**Datasets**					
ZINC 250k (ref. ^52^)	3.993 ± 0.355	1.097 ± 0.192	24.260 ± 0.622	1.020 ± 0.193	6.183 ± 0.344
ChEMBL 33 (ref. ^50^)	6.077 ± 0.453	1.830 ± 0.240	24.163 ± 0.715	2.073 ± 0.181	9.013 ± 0.562
**Generative models**					
REINVENT^13^	4.693 ± 1.776	1.967 ± 0.661	26.047 ± 2.497	2.207 ± 0.800	5.667 ± 1.067
JT-VAE^23^	3.200 ± 0.348	0.933 ± 0.152	18.044 ± 0.747	0.644 ± 0.157	5.856 ± 0.204
GraphAF^80^	0.822 ± 0.113	0.011 ± 0.016	6.978 ± 0.952	1.422 ± 0.556	1.233 ± 0.284
MORLD^81^	0.047 ± 0.050	0.007 ± 0.013	0.893 ± 0.758	0.047 ± 0.040	0.227 ± 0.118
HierVAE^82^	1.180 ± 0.182	0.033 ± 0.030	0.740 ± 0.371	0.367 ± 0.187	0.487 ± 0.183
GraphDF^83^	0.044 ± 0.031	0.000 ± 0.000	0.000 ± 0.000	0.011 ± 0.016	0.011 ± 0.016
FREED^3^	4.860 ± 1.415	1.487 ± 0.242	14.227 ± 5.116	2.707 ± 0.721	6.067 ± 0.790
FREED-QS^3^	5.960 ± 0.902	1.687 ± 0.177	23.140 ± 2.422	3.880 ± 0.623	7.653 ± 1.373
LIMO^84^	0.456 ± 0.057	0.044 ± 0.016	1.200 ± 0.178	0.278 ± 0.134	0.711 ± 0.329
GDSS^85^	2.367 ± 0.316	0.467 ± 0.112	6.267 ± 0.287	0.300 ± 0.198	1.367 ± 0.258
MOOD^4^	7.260 ± 0.764	0.787 ± 0.128	21.427 ± 0.502	5.913 ± 0.311	10.367 ± 0.616
Augmented Memory^45^	16.966 ± 3.224	2.637 ± 0.860	52.016 ± 2.302	8.307 ± 1.714	21.548 ± 4.938
Genetic generative flow networks^86^	8.420 ± 1.794	1.532 ± 0.272	36.122 ± 2.568	3.375 ± 0.634	10.818 ± 1.636
GEAM^5^	**45.158** ± **2.408**	**20.552** ± **2.357**	47.664 ± 1.198	30.444 ± 1.610	46.129 ± 2.073
Saturn (framework)	**57.981** ± **18.537**	**14.527** ± **9.961**	**68.185** ± **3.400**	**38.999** ± **10.114**	**60.827** ± **11.502**

Results are from Lee et al.^4,5^ except Augmented Memory, GEAM, datasets, genetic generative flow networks and Saturn (framework), which we ran across ten seeds (0–9 inclusive). The mean and standard deviation are reported. Best results (statistically significant at the 95% confidence level using a two-sided Welch’s *t*-test) are bolded. Comparing GEAM and Saturn (framework) results, the *P* values are (rounded to four decimal points) as follows: parp1 = 0.0687, fa7 = 0.1078, 5ht1b = 0, braf = 0.03236, jak2 = 0.0039.

Source data

Saturn framework: enhanced MPO. Next, we assess the optimization capability of GEAM and the Saturn framework by applying a strict filter for QED > 0.7 and SA < 3 (Table 2). The results show that GEAM’s hit ratios notably drop, whereas Saturn’s remain relatively unchanged, which demonstrates that Saturn optimizes the MPO objective to a much greater degree (Supplementary Section 5.5 shows the novel strict filter results). Importantly, Saturn finds molecules passing this strict filter with much fewer oracle calls (the OB metrics are listed in Table 2), trading off diversity to do so. For fa7 and braf, GEAM does not find 100 molecules passing the strict filter in 9/10 and 4/10 replicates, respectively, whereas Saturn is successful in 10/10 for both (Table 2).

**Table 2:** Strict hit ratio (%)

Method	Target protein				
	parp1	fa7	5ht1b	braf	jak2
**GEAM**^5^					
Strict hit ratio (*↑*)	6.510 ± 1.087	2.106 ± 0.958	8.719 ± 0.903	3.685 ± 0.524	7.944 ± 1.157
OB (1) (*↓*)	250 ± 157(10)	433 ± 209(10)	114 ± 112(10)	355 ± 96(10)	230 ± 117(10)
OB (10) (*↓*)	743 ± 52(10)	1,446 ± 404(10)	531 ± 38(10)	892 ± 144(10)	537 ± 70(10)
OB (100) (*↓*)	2,106 ± 202(10)	2,927 ± 0(1)	1,527 ± 110(10)	2,674 ± 163(6)	1,606 ± 218(10)
IntDiv1 (*↑*)	0.760 ± 0.016	0.702 ± 0.042	0.792 ± 0.016	0.745 ± 0.023	0.758 ± 0.021
*#*Circles (*↑*)	14 ± 1	6 ± 1	23 ± 2	11 ± 1	17 ± 2
**Saturn (framework)**					
Strict hit ratio	55.102 ± 18.027	13.887 ± 9.723	64.730 ± 3.717	37.250 ± 9.615	55.903 ± 13.613
OB (1) (*↓*)	139 ± 96(10)	352 ± 206(10)	21 ± 7(10)	291 ± 143(10)	88 ± 56(10)
OB (10) (*↓*)	518 ± 92(10)	924 ± 247(10)	105 ± 23(10)	581 ± 123(10)	348 ± 96(10)
OB (100) (*↓*)	956 ± 259(10)	1,776 ± 551(10)	441 ± 44(10)	1,057 ± 187(10)	785 ± 191(10)
IntDiv1 (*↑*)	0.585 ± 0.049	0.583 ± 0.067	0.675 ± 0.021	0.587 ± 0.042	0.628 ± 0.036
*#*Circles (*↑*)	5 ± 1	3 ± 1	17 ± 3	4 ± 1	8 ± 2

GEAM and Saturn results are across ten seeds (0–9 inclusive). The number in the parentheses in the OB statistics represents how many runs out of 10 were successful. The mean and standard deviation are reported. Comparing GEAM and Saturn ‘strict hit ratio’ results at the 95% confidence level using a two-sided Welch’s *t*-test, the *P* values are (rounded to 4 decimal points) as follows: parp1 = 0, fa7 = 0.0054, 5ht1b = 0, braf = 0, jak2 = 0.

**Fig. 3:**
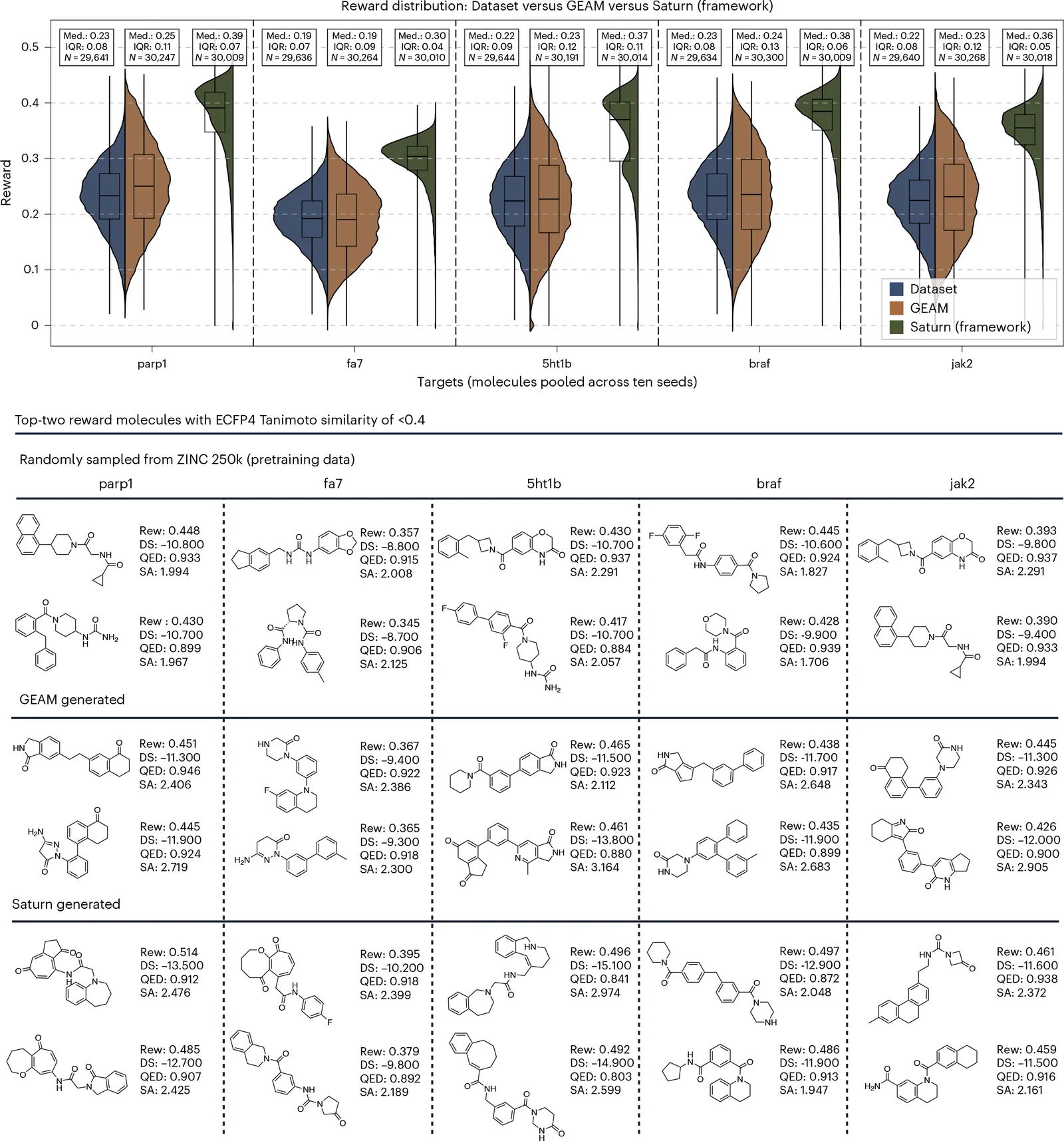
Reward distribution across all five protein targets for molecules randomly sampled from ZINC 250k, GEAM generated and Saturn generated. The molecules are pooled across ten seeds (0–9 inclusive) and molecules that failed to dock were removed. The box-plot whiskers cover the full data range. The middle line is the median (Med.), and the upper and lower lines denote the interquartile range (IQR). The top-two (by reward (Rew)) molecules from each pooled set are shown. QED, quantitative estimate of drug-likeness; SA, synthetic accessibility score; DS, 2 docking score. Note that the pairs of molecules *N* are also dissimilar by an ECFP4 Tanimoto similarity of <0.4. Source data

To further investigate the optimization capability, we pool all molecules across the ten seeds and plot the reward distributions comparing random sampling from the ZINC 250k pretraining dataset, GEAM and Saturn (Fig. 3). GEAM molecules have a similar reward distribution to random sampling, whereas Saturn notably shifts the reward higher. The reason why GEAM and random sampling have similar reward distributions is because the MPO function (equation (1)) jointly considers QED, SA score and docking score. Although GEAM molecules possess better docking scores than random sampling, QED and SA score are less optimal (Supplementary Fig. 6). Supplementary Table 27 also shows that the GEAM-generated molecules have a higher molecular weight and lower ligand efficiency (docking score divided by the number of heavy atoms) compared with Saturn-generated molecules. Finally, Fig. 3 also displays the top-two (by reward) pooled molecules with an ECFP4 Tanimoto similarity of <0.4. Here the results illustrate that GEAM molecules have more favourable docking scores than random sampling, but the QED and SA score are less optimal. By contrast, Saturn jointly optimizes all objectives to a greater degree, ultimately explaining why the reward distribution is shifted to higher values.

### Part 3: directly optimizing electronic properties at the DFT fidelity level

**Fig. 4:**
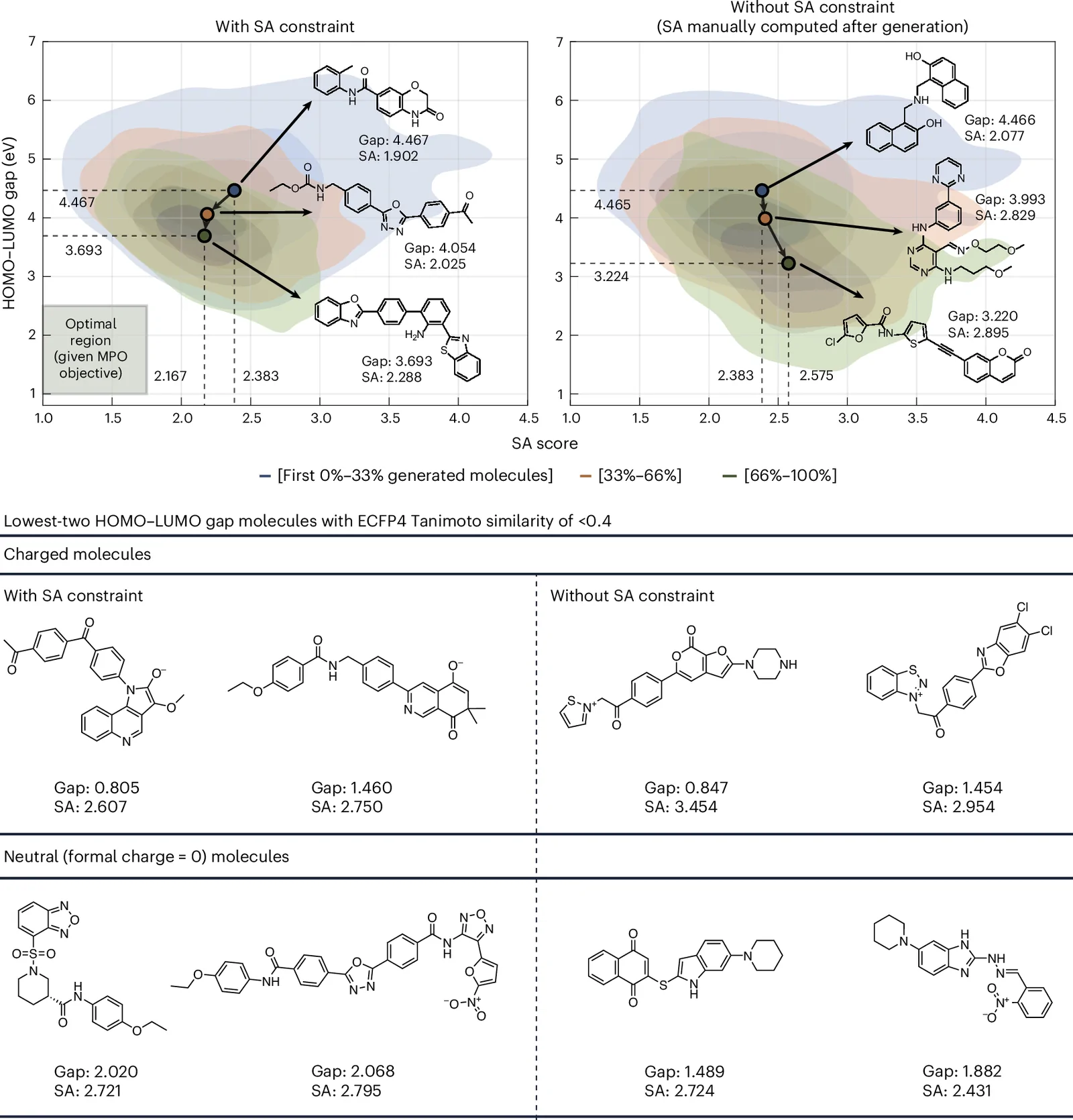
Directly optimizing at the DFT fidelity level with an oracle budget of 500. The optimization objectives are to minimize the HOMO–LUMO gap alone (right plot) or jointly minimizing it with the SA score (left). Molecules are pooled across three seeds (0–2 inclusive) and the contour densities show the shift to lower HOMO–LUMO energies from the first 33% generated molecules to the next 33%, and the final 33%. The dots and annotated molecules are the median HOMO–LUMO value at each fraction. The median HOMO–LUMO energy and SA score between the first and last fractions are also annotated. Removing the SA score constraint results in lower HOMO–LUMO values, as expected. The two lowest HOMO–LUMO gap molecules from each pooled set are shown. Note that the pairs of molecules are also dissimilar by an ECFP4 Tanimoto similarity of <0.4. Source data

In this section, we demonstrate the feasibility of directly optimizing high-fidelity oracles with the Saturn framework. Using DFT to optimize the geometry of all the generated molecules, we target the computed highest occupied molecular orbital–lowest unoccupied molecular orbital (HOMO–LUMO) gap as a property of interest for functional materials design, such as semiconductors^59,60^. The optimization objective is to minimize the HOMO–LUMO gap with and without jointly minimizing the SA score. To keep computational costs manageable, we run three seeds (0–2 inclusive) and enforce a 500 oracle budget (Supplementary Section 6 provides the DFT details). Figure 4 shows a pool of all the generated molecules across the three seeds as well as shows the property densities of the sequential fractions of generated molecules (every 33%). We do this to convey a notion of early stage, middle-stage and late-stage optimization given the highly constrained oracle budget. The Saturn framework successfully shifts the property densities to lower HOMO–LUMO gap values and is affirmed by the annotated molecules on the plot, which are the median HOMO–LUMO gap values.

Next, we show the two-lowest HOMO–LUMO gap molecules with ECFP4 Tanimoto similarity of <0.4 and note that the model implicitly incorporates charged atoms that are involved in resonance and ring conjugation, in agreement with previous observations^60^ (Fig. 4). Neutral molecules are also shown and are also highly conjugated structures. Cross-referencing the density plots, one can observe that without the SA constraint, the generated molecules start to deviate towards higher SA scores. Thus, the bulk statistics reaffirm that the SA constraint does indeed constrain the generative process. Overall, the results demonstrate that it is feasible to directly optimize the electronic properties at the DFT level of theory using the Saturn framework.

## Discussion

In this work, we present Saturn, a framework for sample-efficient de novo molecular design. We use the Mamba^49^ architecture with RL and show how it intensifies the effect of Augmented Memory^45^, which combines SMILES augmentation and experience replay for enhanced sample efficiency. We elucidate the mechanism of Augmented Memory and show it makes generating varying SMILES forms of the replay buffer molecules more likely (Fig. 2d). By investigating the sample efficiency–diversity trade-off across architectures (long short-term memory^11^ RNN, decoder transformer^16,17^ and Mamba^49^), we show that models with increased distribution learning capacity enhance Augmented Memory’s effect. This leads to generated molecules being more similar, which can be beneficial for sample-efficient optimization, but comes at the expense of sample diversity.

The Saturn framework can directly optimize for high-fidelity oracles, such as DFT, as shown in the ‘Part 3: directly optimizing electronic properties at the DFT fidelity level’ section. However, there are two particularly important limitations we highlight. First, methods such as molecular mechanics Poisson–Boltzmann surface area^61^ or free energy perturbation^7^ generally have improved predictive accuracy of binding affinity over molecular docking used in this work. Accordingly, existing studies have generated molecules directly optimizing for molecular mechanics Poisson–Boltzmann surface area^62^ or absolute free energy perturbation^8^. Although the current Saturn framework was validated on a DFT case study, future work will extend to these high-fidelity binding affinity oracles. Another limitation is the synthesizability of the generated molecules. Although some experiments incorporated the SA score^54^ in the optimization objective, running explicit retrosynthetic analysis would better assess synthesizability and guide the model towards the synthesizable space^63^. Subsequently, follow-up works have coupled retrosynthesis models with the Saturn framework to enable generating molecules predicted to be synthesizable using specific building block sets, enforcing or avoiding specific reactions and minimizing the synthesis route length^64,65^.

Augmented Memory algorithm^45^ makes generating the replay buffer molecules more likely, leading to local exploitation. In the case studies performed, this behaviour was beneficial for sample efficiency. However, this will not generalize to every optimization objective, as structure–property relationships can impose troughs in the reward signal. A concrete and extreme example is a steep ‘activity cliff’ in which a small change (even a single atom) elicits a large property value difference (for instance, binding affinity)^66^. In these scenarios, generating similar molecules can be disadvantageous and a more exploratory strategy would probably perform better. Therefore, an important research avenue is to characterize the failure modes of the Saturn framework and enable future generative workflows to more optimally or adaptively balance the exploration–exploitation trade-off when faced with an arbitrary optimization objective. Relatedly, existing studies have investigated parallel generative models, each separately tasked with exploration and exploitation^67^, or an ensemble of cooperating generative models to better navigate the design space^68^.

## Methods

In this section, each component of the Saturn framework (Fig. 1) is described: the language model backbone for molecular generation, the Augmented Memory^45^ RL algorithm, the GA, and specific details into key components responsible for sample efficiency and mitigating mode collapse.

Autoregressive language model backbone for molecular generation: molecules are represented as SMILES^10^ and the task of goal-directed generation is cast as an RL problem. Let *S*_*t*_ denote the state space representing all intermediate token sequences during molecular generation. The action space, *A*_*t*_(*s*_*t*_), is defined as the conditional token distribution induced by the policy, *π*_*θ*_, and parameterized by a language model backbone. In this work, we investigated RNN, decoder transformer and Mamba backbones with the latter chosen as the default after extensive experimentation. Therefore, *θ* represents the parameters of the Mamba backbone^49,69^, which is a state space model^70,71^ and features four learnable matrices to propagate sequence information $\bar{{\bf{A}}},\bar{{\bf{B}}},\bar{{\bf{C}}}\,\mathrm{and}\,\bar{{\bf{D}}}$:

$$\begin{array}{rcl}{h}_{t} & = & \bar{{\bf{A}}}{h}_{t-1}+\bar{{\bf{B}}}({x}_{t}){x}_{t}\\ {y}_{t} & = & \bar{{\bf{C}}}({x}_{t}){h}_{t}+\bar{{\bf{D}}}{x}_{t},\end{array}$$

where *h* is the state, *x* is the input sequence (SMILES in this work) and *y* is the output. Importantly, the input-dependent parameters confer a selective mechanism that can handle contextual importance, and notably differs from previous state space models^70,71^. Like other language models, a linear projection transforms the Mamba output to a multinomial token distribution. This enables sequence generation, which we define as a Markov process, and thus, sampling a SMILES, *x*, is given by the product of conditional token probabilities (equation (2)):

2 $$P(x)=\mathop{\prod }\limits_{t=1}^{T}{\pi }_{{\theta }_{{\rm{Agent}}}}({a}_{t}| {s}_{t}),$$

where *π*_*θ*_ is the Mamba backbone and referred to as the agent to match RL terminology and *a*_*t*_ and *s*_*t*_ are the token selected and token sequence so far, at time-step *t*, respectively. We couple RL to the generative process to enable MPO. The general objective in RL is to maximize the expected reward (equation (3)):

3 $$J(\theta )={{\mathbb{E}}}_{{a}_{t}\approx {\pi }_{{\theta }_{\mathrm{Agent}}}}\left[\mathop{\sum }\limits_{t=1}^{T}R({a}_{t},{s}_{t})\right].$$

*R* is the reward function and can represent any arbitrary MPO objective and *σ* is a scalar factor modulating its effect. Next, the Augmented Likelihood^13^ (equation (4)) is defined, where the prior is the pretrained model with frozen weights:

4 $$\log [{\pi }_{{\rm{Augmented}}}(x)]=\log [{\pi }_{{\rm{prior}}}(x)]+\sigma R(x).$$

The reward is defined as $\log [{\pi }_{\mathrm{Augmented}}]$ – $\log [{\pi }_{{\theta }_{\mathrm{Agent}}}]$. Following previous works^13,45,72^, maximizing equation (3) is equivalent (up to a factor) to minimizing the squared difference between the Augmented and Agent Likelihoods (equation (5)):

5 $$L(\theta )=\frac{1}{| B| }{\left[\mathop{\sum }\limits_{a\in {A}^{* }}(\log [{\pi }_{\mathrm{Augmented}}]-\log [{\pi }_{{\theta }_{\mathrm{Agent}}}])\right]}^{2}.$$

A* is defined as the actions taken across all time steps in a given batch. During optimization, the expected reward (equation (3)) is approximated by sampling a batch, *B*, of SMILES. The batch size controls for variance as approximating the expectation with fewer samples is necessarily more noisy. Supplementary Section 1.5 provides complete details on the algorithm and pseudo-code.

Augmented Memory: in the Saturn framework, Augmented Memory maintains a replay buffer of the top-100 SMILES ranked by their rewards. At each generation epoch, the agent is updated *N* augmentation rounds times. Each augmentation round involves taking every SMILES in the buffer, augmenting (randomizing)^40^ them and updating the agent following equation (5). A diversity filter^73^ stores the Bemis–Murcko^74^ scaffolds of every SMILES generated. If a scaffold is generated more than a permitted threshold (*M* = 10 in this work), its reward is truncated to 0. Before executing Augmented Memory, scaffolds associated with penalized rewards are purged from the buffer, preventing mode collapse.

GA: the Saturn framework adapts GraphGA^30^ in which the replay buffer is treated as the parent population. In this work, the replay buffer stores the top-100 SMILES generated so far (by reward) and serves as the parent population. When activated, at each generation epoch, the GA performs ‘cross-over’ and ‘mutation’ operations. Concretely, the two parents are selected from the pool of top-100 SMILES with a probability proportional to their reward and converted to RDKit^75^ ‘Mol’ graphs. All GA operations are performed on this molecular graph representation. Cross-over selects, with equal probability, cutting the parent molecules either at ring or non-ring positions. The resulting fragments are combined to produce the ‘child’ molecule. In particular, the child molecule is only kept if it satisfies the following hard constraints: 15 < number of atoms < 40, the maximum ring size is <7, no allenes in rings and no double bond in a three- or four-membered ring. Subsequently, the child molecule undergoes random mutation operations that can include inserting or deleting atoms, changing atoms or changing bonds. In total, 100 molecules are produced using the GA and a sampled subset is added to the generative model’s sampled batch before executing Augmented Memory. Our main hypothesis is as follows: since Augmented Memory learns repeatedly from the same molecules, this inherently causes the agent to generate less diverse molecules over time. For Augmented Memory to enhance the sample efficiency, the replay buffer should be populated by high-reward molecules. Therefore, our main hypothesis is that executing GraphGA on these high-reward molecules will, on average, yield other molecules that are also high reward and more diverse. Since GA-resulting high-reward molecules will refresh the buffer, gaining access to diverse, high-reward molecules might be able to offer a better sample efficiency–diversity trade-off. Implementation wise, after executing GraphGA on the replay buffer molecules, we experiment with selecting five or ten GA-derived molecules to keep by either (1) random sampling or (2) selecting based on dissimilarity (Tanimoto Morgan fingerprint) to the buffer molecules. Supplementary Section 2.4 provides more details.

### Reporting summary

Further information on research design is available in the Nature Portfolio Reporting Summary linked to this article.

## Supplementary information

Supplementary Information Supplementary Sections 1–6, Figs. 1–6 and Tables 1–31.

Reporting Summary

## Source data

Source Data Fig. 2 Values shown in Fig. 2a,b.

Source Data Fig. 3 Raw reward values for the three methods shown in Fig. 3: Saturn (framework), GEAM and ZINC 250k sampling. Values provided for all five docking protein targets.

Source Data Fig. 4 Raw data for the three fractions used in Fig. 4.

Source Data Table 1 Raw hit ratios for the models we ran: Augmented Memory, GEAM, Saturn (framework) and genetic generative flow networks.

Source Data Fig. 6 Raw GEAM and Saturn (framework) values for all metrics in the table.

Source Data Extended Data Fig./Table 1 Values in the table.

Source Data Extended Data Fig./Table 2 Raw novel hit ratio values for the models we ran: GEAM, Saturn (framework) and Saturn–Tanimoto (framework).

## Figures and Tables

**Extended Data Table 1:**
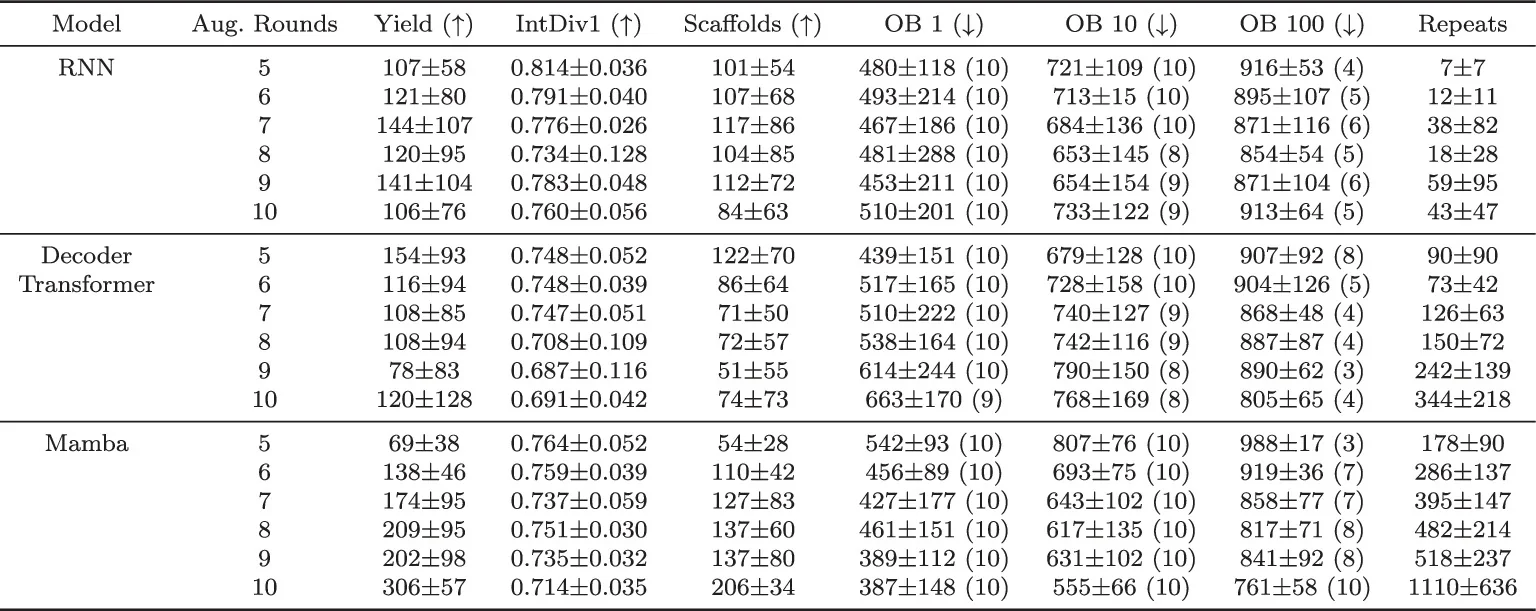
Sample efficiency across architectures (batch size 16)

1,000 oracle budget. All metrics are computed at the 0.7 reward threshold. IntDiv1 is the internal diversity, Scaffolds is the number of unique Bemis-Murcko scaffolds, OB is Oracle Burden (oracle calls required to generate N unique molecules). The number in parentheses in the OB statistics represents how many runs out of 10 were successful. Repeats are the number of times an identical SMILES was generated during the run. The mean and standard deviation across 10 seeds (0-9 inclusive) is reported.

Source data

**Extended Data Table 2:**
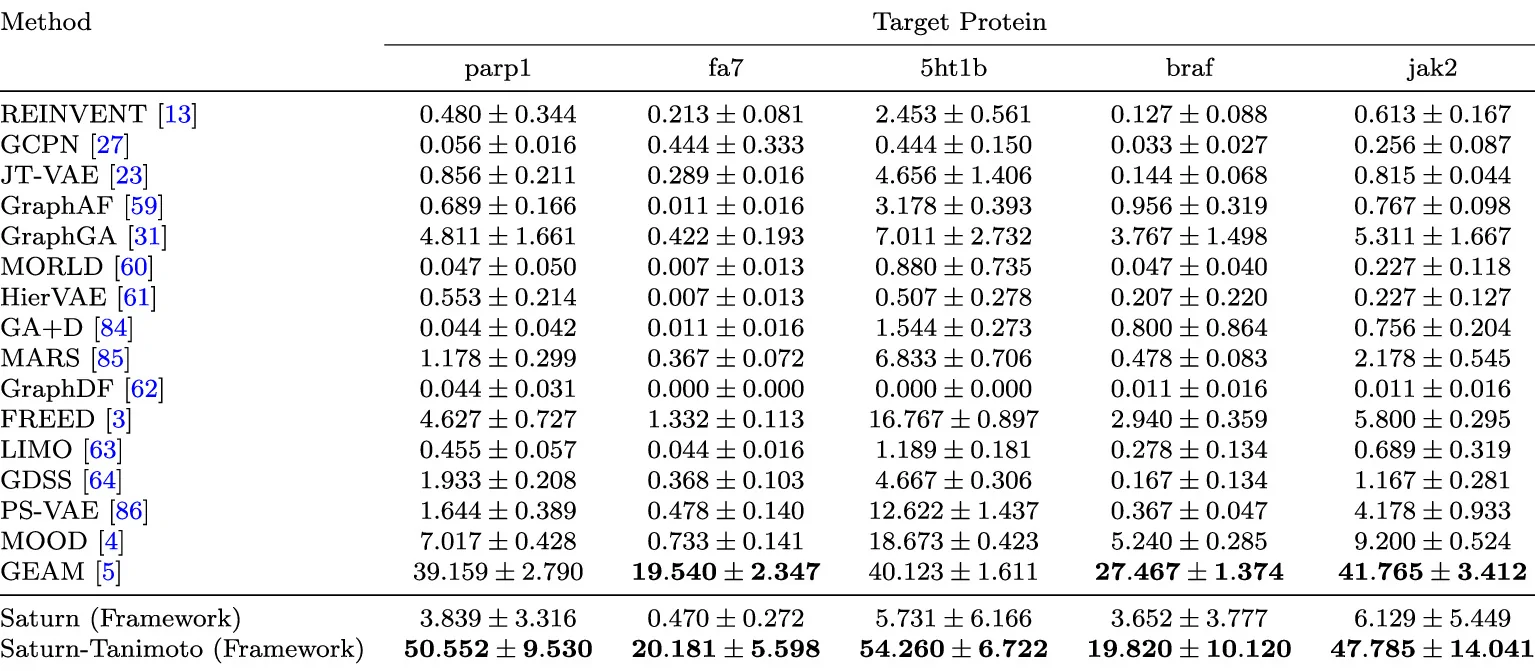
Novel Hit Ratio (%)

Results are from Lee et al. except GEAM and the Saturn (Framework) which we ran across 10 seeds (0-9 inclusive). The mean and standard deviation are reported. Best results (statistically significant at the 95% confidence level using a two-sided Welch’s t-test) are bolded. Comparing GEAM and Saturn-Tanimoto (Framework) results, the p-values are (rounded to 4 decimal points): parp1 = 0.0059, fa7 = 0.7567, 5ht1b = 0.0001, braf = 0.0503, jak2 = 0.2397.

Source data

## Data Availability

The datasets used for pretraining are ChEMBL 33 (ref. ^50^) and ZINC 250k (ref. ^52^), which can be freely downloaded from https://ftp.ebi.ac.uk/pub/databases/chembl/ChEMBLdb/releases/chembl_33/ and https://github.com/SeulLee05/GEAM/tree/main/data, respectively. The instructions for processing these datasets are provided in Supplementary Section 1.6. Additionally, the pretrained models are available via GitHub at https://github.com/schwallergroup/saturn/tree/master/experimental_reproduction/checkpoint_models. The archived codebase version used in this work is available via Figshare at https://doi.org/10.6084/m9.figshare.30968380 (refs. ^76–79^). Source data are provided with this paper.

## Code availability

The prepared files and instructions to reproduce the experiments are available via GitHub at https://github.com/schwallergroup/saturn/tree/master/experimental_reproduction. The archived codebase version used in this work is available via Figshare at https://doi.org/10.6084/m9.figshare.30968380 (ref. ^79^).
