# Supplementary material for: Sample-efficient generative molecular design using memory manipulation
Source: Nat Mach Intell. 2026 Mar 17;8(3):449–60. doi: 10.1038/s42256-026-01200-4 (PMC13658037; doi:10.1038/s42256-026-01200-4)
Supplement: Supplementary file 1 [file 42256_2026_1200_MOESM1_ESM.pdf]

nature machine intelligence

Supplementary information

https://doi.org/10.1038/s42256-026-01200-4

Sample-efficient generative molecular design using memory manipulation

In the format provided by the authors and unedited

Contents

1 What is Saturn? 1

1.1 Generative Architecture . . . 3

1.2 Mamba Architecture . . . 4

1.3 Oracle Caching . . . 5

1.4 Genetic Algorithm . . . 6

1.5 Full Algorithm Details and Pseudo-code . . . 7

1.6 Data Pre-processing and Pre-training . . . 13

1.7 Computational Requirements . . . 15

2 Part 1: Elucidating the Optimization Dynamics of Augmented Memory 16

2.1 Understanding the Limits of Augmented Memory . . . 17

2.2 Do Architectures Differ in Behavior? . . . 23

2.3 Are Increased Augmentation Rounds Tolerable with Beam Enumeration? . . . 35

2.3.1 Hypothesis 1 . . . 36

2.3.2 Hypothesis 2 . . . 39

2.3.3 Hypothesis 3 . . . 42

2.4 Hallucinated Memory: Is it beneficial to allocate a portion of the oracle budget to hallucination? . . . 46

2.5 Saturn: Final Hyperparameters . . . 48

3 Mechanism of Augmented Memory and Mamba 51

3.1 Is "Hop-and-locally-exploit" *Always* Good? . . . 54

3.2 Genetic Algorithm Loosens "hop-and-locally-exploit Behavior" . . . 54

4 Transferability of Sample Efficiency to Physics-based Oracles 56

4.1 Quantitative Results . . . 56

4.2 Docking Protocol . . . 56

4.3 Additional Results . . . 58

4.4 Compute Time . . . 58

5 Part 2: Benchmarking the Saturn Framework and Demonstrating Enhanced Optimization 61

5.1 Saturn ZINC 250k Pre-training 61
5.2 Reproducing GEAM's Results 62
5.3 GEAM's MPO Objective 62
5.4 Saturn-Tanimoto 63
5.5 Quantitative Supplementary Results 63
5.6 Novelty Metrics 69
5.7 Saturn Framework: Architecture Scaling 69
5.8 Qualitative Supplementary Results 76

6 Part 3: Directly Optimizing Electronic Properties at the DFT Fidelity-level 78

6.1 Details On The DFT Simulations To Compute The HOMO-LUMO Gap 78
6.2 Potential Challenges when Pushing Towards Other High-fidelity Oracles 78
6.3 Saturn Framework: Details Specific to Running DFT 79
6.4 Novelty Metrics 79

1 What is Saturn?

Saturn is a language-based generative molecular design framework which features minimal implementations of Augmented Memory [1] and Beam Enumeration [2]. These two methods were first implemented here: https://github.com/schwallergroup/augmented_memory, which in turn was built on REINVENT version 3.2 [3, 4]: https://github.com/MolecularAI/Reinvent. REINVENT is still under active development and version 4 [5] was recently released, supporting a wide range of generative tasks including small molecule design [3, 4], library design [6], linker design [7], proposing small modifications [8], and sampling nearest neighbors [9].

Saturn (at the moment) focuses only on generative small molecule design and research development is on sample efficiency. It is a much smaller code-base than REINVENT 4 and with focus on minimal implementation. That being said, the new additions to Saturn include: extending small molecule generative architecture from just RNN in REINVENT to Decoder transformer [10, 11] and Mamba [12]. Secondly, allowing oracle caching to track repeated generations and allow pre-screening specified oracles (in an MPO objective, some oracle components may be computationally inexpensive and it can be practical to first screen molecules through these oracles before any expensive components). Thirdly, integration of a genetic algorithm (GraphGA [13]) on the replay buffer such that new molecules can be generated from the replay buffer parent sequences. In the ensuing subsections, we describe in detail these new additions.

Differences to Previous Works. Saturn adapts Augmented Memory [1] but differs in several important ways. Firstly, unlike the original work, we elucidate the mechanism into *why* Augmented Memory can improve sample efficiency and *explicitly* show that it makes generating the replay buffer molecules likely. The Results section shows that high sample efficiency can be achieved by *local sampling*, whereby the modeled distribution is strategically *overfit* on these replay buffer molecules. Precisely, this means making the Agent particularly likely, but *not* deterministic, to generate *any SMILES sequence form* of the replay buffer molecules. By nature of multinomial decoding, stochastic generation means that unique sampled molecules might only differ by a small number of tokens, which translates to the molecules differing by a small number of atoms. Secondly, we show that Mamba enhances this mechanism by nature of being a proficient distribution learner. Exactly because Mamba can overfit the distribution of replay buffer molecules, it displays

the greatest degree of *local sampling*. To accommodate repeat generated molecules, we introduce an oracle cache under the assumption that oracle evaluations are *near deterministic*. If the same SMILES is generated at a later epoch, the reward is retrieved from the cache and does not impose an oracle call. By showing that strategic overfitting can be beneficial, we further demonstrate that scaling up architectures (Supplementary 5.7) can also improve sample efficiency, by increasing the model's distribution learning capacity. This offers discourse into the benefits of architectural differences in the small molecule goal-directed generation regime. We show that there are benefits despite the modeled sequences being relatively short ($<$ 80 tokens). Most generally, the results demonstrate the mechanism in which SMILES augmentation modulates the sample-efficiency-diversity trade-off.

Lastly, we highlight existing work that uses an RNN to sample molecules and stores them in a replay buffer. The RNN itself is from REINVENT [3], which Augmented Memory [1] builds on. GraphGA [13] is used to generate other molecules from the replay buffer to further populate it (truncating the buffer if it exceeds the maximum permitted size). Finally, batches of molecules are sampled from the replay buffer numerous times and used to train the model, which is named Genetic GFlowNet (GFN) [14]. The Saturn framework and Genetic GFN have numerous similar components but the methods differ algorithmically in the following ways: (1) Genetic GFN does not update the model with sampled batches of molecules and only learns from batches drawn from the replay buffer. The Saturn framework learns from both sampled batches and the replay buffer. (2) Both methods learn repeatedly (we denote this as N times here) from the replay buffer's molecules. The difference is that Genetic GFN up-weights the molecules that have higher reward and samples a batch, trains the model using this batch, and repeats N times, while the Saturn framework uses the entire replay buffer (based on Augmented Memory). (3) When using GraphGA, both method apply it on the replay buffer molecules. Genetic GFN again samples using a re-weighting strategy based on reward while the Saturn framework uses the default GraphGA sampling with probability proportional to the reward. The difference is that Genetic GFN performs the genetic process numerous times, while the Saturn framework only performs it once. We note that the rationale to use GraphGA in both works is to improve exploration. (4) Genetic GFN is trained using trajectory balance in GFlowNets [**?**]. By contrast, the Saturn framework uses REINVENT's [3] loss, which updates the agent (model being trained) based on the reward, but prevents the model

from deviating too far from the prior (pre-trained model). (5) The Saturn framework extends the model architecture from RNN to decoder transformer and Mamba and studies the effect of the architecture and how distribution learning capacity affects the model's sampling behavior. (6) Saturn adapts Augmented Memory, which in turn uses diversity filters [16] (described in the Augmented Memory section above) to prevent mode collapse, which can occur when learning repeatedly from the entire replay buffer, as observed in the original Augmented Memory work [1]. Genetic GFN is likely less prone to mode collapse as it samples batches from the replay buffer. We note that this work and the Augmented Memory study show that learning from the entire replay buffer can be especially effective in enhancing sample efficiency. Overall, we explicitly highlight the similarities and differences between these approaches and cross-reference the main text results showing that the Saturn framework outperforms Genetic GFN (both using default parameters).

1.1 Generative Architecture

Many initial language-based molecular generative models were RNN-based [3, 17–19]. Early benchmarks (GuacaMol [20] and MOSES [21]) assessed whether generated molecules were valid (RDKit parsable), unique, and novel (not in the training data). RNNs satisfy these metrics and can learn distributions well [22]. More recently, with the prevalence of the transformer [10, 11] architecture, many works [23–30] have suggested a replacement of RNNs for generative design. However, many performance assessments only focus on validity, uniqueness, novelty, and optimizing for permissive oracles such as logP, QED [31] ("drug-likeness"), and the SA score [32]. Some works show that transformers can learn longer SMILES sequences better than RNNs [25] (such as natural products). However, often, one actually *wants* to limit sequence length to constrain design to small molecules. Furthermore, recent works have swapped the RNN architecture with transformers and optimized for various molecular property objectives using reinforcement learning (RL) [25–29]. However, the performance of the transformer backbone was not necessarily better than RNNs. Consequently, it is unclear whether the benefits of transformers are strictly advantageous for small molecule generation.

In this work, we extend Augmented Memory [1] to decoder transformer [10, 11] and Mamba [12]. Our results show that transformers display similar performance to RNNs for small molecule

generation, in agreement with previous literature findings [29]. We further demonstrate the application of Mamba [12] for goal-directed generation, supplementing recent work investigating S4 models for transfer learning [33].

1.2 Mamba Architecture

In Saturn, we empirically find that the Mamba [12] architecture is the most parameter-efficient in our RL framework when tuning for sample efficiency. Note that in Supplementary Information 5.7, we find that scaling up the decoder transformer (25.3M) [10, 11] to about 5x the size of the Mamba (5.2M) results in similar performance. However, due to less GPU load for smaller models, we chose Mamba as the default architecture in Saturn. Mamba was recently proposed as an alternative to transformers, with linear time training in contrast to the quadratic attention scaling. In decoder transformers, self-attention is the key component:

$$\text{Attention}(Q, K, V) = \text{softmax}\left(\frac{QK^\top}{\sqrt{d_k}}\right)V$$

where embedded input (SMILES [34] sequences in our case), X, is multiplied with the weight matrices, W_Q, W_K, W_V to produce the Query (Q), Key (K), and Value (V) matrices, respectively. QK^T results in an N x N (sequence length) matrix and leads to an overall $O(N^2)$ scaling. However, an advantage of self-attention is that during inference, the entire context is available without any compression.

We now contrast the information flow in Mamba, with all information adapted from, and following the convention in preceding work on state-space models [37], structured S4 [35], the original Mamba [12] work and a technical blog [36]. Mamba builds on state-space models, which propagate information through four learnable matrices: $\mathbf{A}$, $\mathbf{B}$, $\mathbf{C}$, and $\mathbf{D}$:

$$h'(t) = \mathbf{A}h(t) + \mathbf{B}x(t)$$
$$y(t) = \mathbf{C}h(t) + \mathbf{D}x(t)$$

where h is the state, x is the input sequence, and y is the output. Therefore $\mathbf{A}$ and $\mathbf{B}$ dictate how the state changes as a function of the current state and input, respectively. This is similar for $\mathbf{C}$ and $\mathbf{D}$ on the output. As $\mathbf{A}$, $\mathbf{B}$, $\mathbf{C}$, and $\mathbf{D}$ are fixed for all t, this is *time-invariant*. As we

are working with discrete data (SMILES tokens in our case), the continuous form is discretized [35, 37]:

$$h_t = \bar{\mathbf{A}} h_{t-1} + \bar{\mathbf{B}} x_t$$
$$y_t = \bar{\mathbf{C}} h_t + \bar{\mathbf{D}} x_t$$

The original works [35, 37] show that the model can be trained efficiently using a continuous *convolution* while offering efficient inference through a *recurrent* mode. Note that in some references, $\mathbf{D}$ is omitted and does not transform the input, x [38]. In the current formulation which is time-invariant, all states and input are transformed in the same way. However, it would be advantageous to process information differently depending on whether it is *more relevant*, similar to self-attention. Correspondingly, Mamba [12] extends this framework and removes the time-invariance constraint by making the $\mathbf{B}$, $\mathbf{C}$, and $\mathbf{D}$ matrices dependent on the input (the following notation follows [36]):

$$h_t = \bar{\mathbf{A}} h_{t-1} + \bar{\mathbf{B}}(x_t) x_t$$
$$y_t = \bar{\mathbf{C}}(x_t) h_t + \bar{\mathbf{D}} x_t$$

The input-dependent parameters allow Mamba to *selectively* propagate information. Removing time-invariance prevents efficient training with continuous convolution and the Mamba [12] authors propose an efficient recurrent *scan* in place. In Saturn, we use their optimized training and inference by adapting the code from the official Mamba repository: `https://github.com/state-spaces/mamba`.

We end this section by conveying that we were not particularly worried about the training and inference speed, since we are working with small molecules with relatively short sequences (typically < 80 tokens). The bottleneck is the reward computation, especially if the oracle is expensive. We were interested in studying the optimization dynamics and how efficiently each model can be tuned via RL.

1.3 Oracle Caching

In many reinforcement learning (RL) set-ups, the reward is assumed to be *stationary*, i.e., it does not change on repeat evaluation. This is an assumption that is not always true for physics-based or-

acles relevant in drug discovery. For example, docking depends on the initial conformer generated, and even more so for molecular dynamics simulations. However, it is reasonable to assume that the reward is *near deterministic* given a reasonably well behaved protein system (in which preliminary studies were made to verify the oracle stability). In effect, the reward for repeat molecules can be retrieved from a cache, thus not imposing additional oracle evaluations. In this work, we show that under this assumption, Saturn can leverage the Mamba [12] architecture for enhanced sample efficiency. In particular, Mamba displays low uniqueness, but we show this is not detrimental.

As any given molecule can have numerous SMILES representations (via augmentation [39]), it is important to store the *canonical* SMILES in the cache, and also to canonicalize sampled batches when querying the cache. Canonicalization is simply a pre-defined traversal and can differ depending on the method used. As long as all canonicalization operations are performed with the same method, consistency can be guaranteed. In this work, we use RDKit.

1.4 Genetic Algorithm

Genetic algorithms (GAs) by themselves can be sample-efficient molecular optimizers [13, 40, 41]. Previous work has shown that GAs can improve diversity of the generated set [42]. Recently, Lee et al. [43] proposed Goal-aware fragment Extraction, Assembly, and Modification (GEAM) which combines RL with a GraphGA [13] and achieves impressive results on generating diverse hits. In Saturn, we implement GraphGA on the replay buffer itself, treating the highest rewarding molecules generated in the entire run so far, as the parent population. Following GEAM [43], sampling the parents is done with probability proportion to their corresponding rewards. New molecules from crossover and mutation operations are deposited into the Buffer if they are also high rewarding, essentially *refreshing* the buffer, such that Augmented Memory [1] can learn from these new SMILES. The motivation was to leverage the GA to counteract decreases in diversity and potentially improve sample efficiency. In the results in the main text and in the following sections, we show that applying the GA does not lead to improved sample efficiency but does indeed maintain diversity. We believe that this can be a useful modification to the optimization algorithm in cases where relatively expensive oracles are used and diversity is important due to prevalence of false positives. Concretely, higher-fidelity oracles should in principle model physical behavior

more accurately, such that true positives are more common. This can be shown in previous works where using free energy simulations provide better correlations with binding affinity [44, 45]. In such a case, sample efficiency becomes increasingly important, as the goal is to simply generate molecules satisfying this simulation and lower diversity is not detrimental. However, when using lower-fidelity oracles, more false positives means it is beneficial to have more diverse ideas for downstream triaging. Finally, we note that applying the GA and generating new molecules strictly means they were generated off-policy (in the RL context). Therefore, more meaningful updates to the Agent *may* be achieved with importance sampling [46], which we did not explore in the current work.

1.5 Full Algorithm Details and Pseudo-code

In this section, we derive Saturn's loss function with particular focus on showing its equivalency to maximizing the expected reward. The derivation follows previous works [1, 3, 6] but with added discussion around implications of the loss function. Specifically, Saturn adapts the Augmented Memory [1] algorithm which is in turn based on REINVENT [3–5]. The algorithm itself is reinforcement learning based and can be seen as a modified REINFORCE [47] algorithm. However, while **Saturn (using Mamba with batch size 16 and 10 augmentation rounds)** adapts Augmented Memory, the optimization trajectory is quite different from the original Augmented Memory work due to the "hop-and-locally-exploit" sampling behavior. We will focus on highlighting specific points related to this.

Saturn's Loss Function. We begin by presenting *how* Saturn generates SMILES [34], which is the data representation used. SMILES are sequences of alphanumeric characters that can be parsed and mapped to a molecular graph, i.e., a molecule. As SMILES are text-based, it is straightforward to tokenize them, and pre-training Saturn follows next-token prediction. Saturn generates SMILES in an autoregressive manner and thus, SMILES are generated token-by-token from time-step, t to T. This can be viewed from a reinforcement learning perspective by defining S_t as the state space representing all intermediate token sequences during molecular generation. $A_t(s_t)$ is the action space which involves sampling a token from a conditional probability distribution, given a token sequence so far, i.e., the current state. Mathematically, the probability of sampling a SMILES, x is

given by:

$$P(x) = \prod_{t=1}^{T} \pi_{\theta_{\text{Agent}}}(a_t \mid s_t) \tag{1}$$

Just generating SMILES is often not useful because they should satisfy the target objective. Thus, the base pre-trained model needs to be tuned somehow to achieve this. The end goal is to find a **Policy** (in the reinforcement learning perspective) which dictates with *what* probability SMILES should be generated to optimize an objective function. To this end, we define the **Prior** and the **Agent** which share the same architecture (Mamba) and whose weights are exactly the same at the beginning of a generative experiment. The Prior and Agent are general terms to describe the model states but they both are policies as they both induce a probability of sampling SMILES. However, what is different is that the Prior's weights are frozen so it is *never* updated. By contrast, the Agent *is* updated and is the model that is learning how to generate "good" SMILES. We now discuss how this is achieved. We define the Augmented Likelihood [3] of a SMILES, x, which is a linear combination between the Prior and a reward term:

$$\log \pi_{\text{Augmented}}(x) = \log \pi_{\text{Prior}}(x) + \sigma R(x) \tag{2}$$

$\log \pi_{\text{Prior}}(x)$ is the log-probability of generating a given SMILES, x, under the Prior. Since the Prior's weights are fixed, the probability of sampling a given SMILES *never* changes. Models are typically parameterized by its weights, θ. We take care here and omit θ because the Prior, as stated previously, is not updated. Next, R is the reward function which defines the target objective, e.g., minimize docking score. Note that the reward function can contain multiple objectives, in which case, constituting a multi-parameter optimization objective. For example, in Experiment 3 of the main text, R is comprised of minimizing docking score, maximizing QED score [31], and minimizing SA score [32]. R takes as input a SMILES, x, and returns a scalar reward $\in$ [0, 1]. σ is a hyperparameter that scales the contribution of the reward function. Importantly, given a SMILES, x, a low σ means the Augmented Likelihood converges to the Prior likelihood while a high σ means the Augmented Likelihood is dominated by the reward. In this work, σ is never changed and is 128 as this was found to work well in the original REINVENT work [3].

The loss function is defined as the squared difference between the Augmented Likelihood and

the Agent Likelihood:

$$L(\theta) = (\log \pi_{\text{Augmented}}(x) - \log \pi_{\theta_{\text{Agent}}}(x))^2 \tag{3}$$

$\log \pi_{\text{Agent}}(x)$ is the log-probability of generating a given SMILES, x, under the Agent. Importantly, we explicitly include θ here because the Agent *is* updated. We stop here for a moment to discuss the implications of the loss function. The loss function tries to minimize the distance between the Augmented Likelihood and the Agent likelihood. Since the Augmented Likelihood (Eq. 2 is a linear combination of the Prior likelihood and the reward function, if the Agent generates "bad" SMILES, then the reward goes to 0 and the Augmented Likelihood converges to the Prior Likelihood. In this event, the Agent's weights actually regress back towards the Prior. This is because the Prior is pre-trained on a general dataset containing bio-active molecules (such as ChEMBL [48] and ZINC 250k [49]. The implicit assumption during pre-training is that these general datasets might actually already contain "good" molecules. Therefore, in the event that "bad" molecules are generated, the Prior acts as a "fall-back". On the other hand, when the reward is not 0, the Prior still "anchors" the Agent and does not let its weights deviate *too far* from the Prior (this is controlled by σ). The reason for this is also because the Prior is assumed to potentially already contain "good" molecules. In practice, the Agent can deviate quite far from the Prior [5]. We now discuss an important implication of this loss function in Saturn. Saturn heavily leverages SMILES augmentation [39] as a data augmentation method to learn from the same molecular graph multiple times. Alternative SMILES sequences, while mapping to the same molecular graph, can have drastically different likelihoods. This is shown in Figure 2 in the main text where Saturn is trained to make it likely to generate all of these alternative SMILES forms. However, this does not always work. Because alternative SMILES forms have different likelihoods, there is the possibility that with the right combination of terms in the Augmented Likelihood, that it equals the Agent likelihood. In this case, the loss contribution is 0 so the Agent actually is not tuned to generate that particular SMILES form with higher likelihood. This is a contributing factor to Saturn's "hop-and-locally-exploit" behavior. Given a set of augmented SMILES, if some of these SMILES cancel out in the loss function, then there is a smaller set of augmented SMILES that contribute to the loss function. With a smaller set, overfitting becomes more prone but we show that this mechanism actually benefits sample efficiency.

Finally, Saturn does not generate individual SMILES but rather, batches of SMILES. Therefore, the loss function is a batched loss:

$$L(\theta) = \frac{1}{|B|}\left[\sum_{a \in A^*} (\log \pi_{\text{Augmented}} - \log \pi_{\theta_{\text{Agent}}})\right]^2 \tag{4}$$

The loss magnitude is the mean loss for a given batch, B, of sampled SMILES constructed following the actions, $a \in A^*$.

Minimizing the loss function is equivalent to maximizing the expected reward. In reinforcement learning, the general objective is to maximize the expected reward. In this section, we show how maximizing the expected reward is equivalent to minimizing the loss function. We first further define some preliminaries: sampling trajectories means sampling SMILES in our context. While there are often *intermediate* rewards during trajectory sampling, e.g., a drone tasked to fly to a target location might receive various rewards for how balanced it is during the flight, we set all intermediate rewards to 0. This is because rewards are only meaningful if the SMILES is a valid molecule. Technically, since the reward is directly the reward from the full trajectory, it is actually the **Return** in reinforcement learning terminology, but we use the term reward to match existing literature. Mathematically, the cost function (in reinforcement learning, J is used and we follow this convention) describes the expected reward when taking actions from a policy that is parameterized by a neural network (Mamba in our case):

$$J(\theta) = \mathbb{E}_{a_t \sim \pi_{\theta_{\text{Agent}}}}\left[\sum_{t=1}^{T} R(a_t, s_t)\right] \tag{5}$$

Since the expectation is in discrete space (sampling tokens is a discrete action), the cost function can be rewritten by transforming the expectation to a sum:

$$J(\theta) = \sum_{t=1}^{T} \sum_{a \in A_t} R(a_t, s_t) \pi_{\theta_{\text{Agent}}}(a_t|s_t) \tag{6}$$

The double summation is over all time-steps and actions (which token sampled) following the policy, π_θ. Since we want to maximize the cost function, we take the derivative:

$$\nabla_\theta J(\theta) = \sum_{t=1}^{T} \sum_{a \in A_t} R(a_t, s_t) \nabla_\theta \pi_{\theta_{\text{Agent}}}(a_t|s_t) \tag{7}$$

Next, the log-derivative trick:

$$\nabla_\theta J(\theta) = \sum_{t=1}^{T} \sum_{a \in A_t} R(a_t, s_t) \pi_{\theta_{\text{Agent}}}(a_t|s_t) \nabla_\theta \log \pi_\theta(a_t|s_t) \tag{8}$$

Using the definition of expectation for discrete space again, the cost function is rewritten:

$$\nabla_\theta J(\theta) = \mathbb{E}_{a_t \sim \pi_{\theta_{\text{Agent}}}} \left[\sum_{t=1}^{T} R(a_t, s_t) \nabla_\theta \log \pi_{\theta_{\text{Agent}}}(a_t|s_t) \right] \tag{9}$$

Computing the expectation exactly is intractable. This would involve sampling every single SMILES and computing their rewards. Therefore, the expectation is approximated by sampling a batch, B, of SMILES. Next, the set of actions taken in a batch at every time-step, is denoted A^*, which yield the specific SMILES generated:

$$\nabla_\theta J(\theta) = \frac{1}{|B|} \left[\sum_{a \in A^*} R(a_t, s_t) \nabla_\theta \log \pi_{\theta_{\text{Agent}}}(a_t|s_t) \right] \tag{10}$$

The reward, R is defined according to previous works [1, 3, 6]:

$$R(a_t, s_t) = \log \pi_{\text{Augmented}} - \log \pi_{\theta_{\text{Agent}}} \tag{11}$$

Substituting the reward function:

$$\nabla_\theta J(\theta) = \frac{1}{|B|} \left[\sum_{a \in A^*} \log \pi_{\text{Augmented}} - \log \pi_{\theta_{\text{Agent}}} \right] \sum_{a \in A^*} \nabla_\theta \log \pi_{\theta_{\text{Agent}}}(a_t|s_t) \tag{12}$$

Recalling the loss function:

$$L(\theta) = \frac{1}{|B|} \left[\sum_{a \in A^*} (\log \pi_{\text{Augmented}} - \log \pi_{\theta_{\text{Agent}}}) \right]^2 \tag{13}$$

Minimizing the loss function requires taking the derivative with respect to θ:

$$\nabla_\theta L(\theta) = -2 \frac{1}{|B|} \left[\sum_{a \in A^*} \log \pi_{\text{Augmented}} - \log \pi_{\theta_{\text{Agent}}} \right] \sum_{a \in A^*} \nabla_\theta \log \pi_{\theta_{\text{Agent}}} \tag{14}$$

The cost function (Eq. 12) is equivalent to the loss function (Eq. 14) up to a factor. The pseudo-code is presented on the following page.

Algorithm 1 Saturn Goal-directed Generation (Without Genetic Algorithm)

Input: Oracle Budget $Budget$, Prior π_{Prior}, Augmentation Rounds A, Reward Function R, Sigma σ, Replay Buffer Size K

Output: Fine-tuned Agent Policy $\pi_{\theta_{\text{Agent}}}$, Generated Set G

Initialization: Generative Agent: $\pi_{\theta_{\text{Agent}}} = \pi_{\text{Prior}}$, Diversity Filter: DF, Replay Buffer: $RB = \{\}$, Oracle Cache: $Cache = \{\}$, Oracle Calls: $C = 0$

while $C < Budget$ **do**

  Sample batch of SMILES $X = \{x_1, \ldots, x_b\}$ with $x_i \sim \pi_{\theta_{\text{Agent}}}$;

  if X *in* $Cache$ **then**

    Retrieve rewards R_{Cached}

  Compute reward for *new* SMILES $R(X_{\text{New}})$;

  Update Oracle Calls and Cache $C = C + |X_{\text{New}}|$, $Cache = ((X_{\text{New}}, R_{\text{New}}) \cup Cache)$;

  $R(X) = R_{\text{Cached}} \cup R(X_{\text{New}})$;

  Modify rewards based on the Diversity Filter $R(X) = DF(X, R(X))$;

  Update Replay Buffer $RB = TopK(X \cup RB)$;

  Compute Augmented Likelihood $\log \pi_{\text{Aug}}(X) = \log \pi_{\text{Prior}}(X) + \sigma R(X)$;

  Compute loss $J(\theta) = (\log \pi_{\text{Aug}} - \log \pi_{\theta_{\text{Agent}}}(X))^2$;

  Update the Agent $\pi_{\theta_{\text{Agent}}}$;

  Purge Replay Buffer;

  for $i \leftarrow 1$ **to** A **do**

    Augment sampled SMILES **and** Buffer SMILES $X_{\text{Augmented}}$;

    Compute Augmented Likelihood of augmented SMILES (reward is unchanged) $\log \pi_{\text{Aug}_{\text{Augmented}}} = \log \pi_{\text{Prior}}(X_{\text{Augmented}}) + \sigma R(X_{\text{Augmented}})$;

    Compute loss $J(\theta)_{\text{Augmented}} = (\log \pi_{\text{Aug}_{\text{Augmented}}} - \log \pi_{\theta_{\text{Agent}}}(X_{\text{Augmented}}))^2$;

    Update the Agent $\pi_{\theta_{\text{Agent}}}$;

1.6 Data Pre-processing and Pre-training

Before presenting grid-search results, we first describe the full data pre-processing pipeline and design decisions made. The pre-training data for all experiments except **Part 2: Benchmarking Saturn and Demonstrating Enhanced Optimization** in the main text (ZINC 250k [49] instead), was ChEMBL 33 [48]. We first downloaded the raw ChEMBL 33 from: https://ftp.ebi.ac.uk/pub/databases/chembl/ChEMBLdb/releases/chembl_33/. There was no particular reason version 33 was chosen, other than it was the latest version at the time of experiments.

The exact pre-processing steps along with the SMILES remaining after each step are:

1. Raw ChEMBL 33 - 2,372,674
2. Standardization (charge and isotope handling) based on https://github.com/MolecularAI/ReinventCommunity/blob/master/notebooks/Data_Preparation.ipynb. All SMILES that could not be parsed by RDKit were removed - 2,312,459
3. Kept only the unique SMILES - 2,203,884
4. Tokenize all SMILES based on REINVENT's tokenizer: https://github.com/MolecularAI/reinvent-models/blob/main/reinvent_models/reinvent_core/models/vocabulary.py
5. Keep SMILES $\leq$ 80 tokens - 2,065,099
6. 150 $\leq$ molecular weight $\leq$ 600 - 2,016,970
7. Number of heavy atoms $\leq$ 40 - 1,975,282
8. Number of rings $\leq$ 8 - 1,974,522
9. Size of largest ring $\leq$ 8 - 1,961,690
10. Longest aliphatic carbon chain $\leq$ 5 - 1,950,213
11. Removed SMILES containing the following tokens (due to undesired chemistry and low token frequency): [S+], [C-], [s+], [O], [S@+], [S@@+], [S-], [o+], [NH+], [n-], [N@],

[N@@], [N@+], [N@@+], [S@@], [C+], [S@], [c+], [NH2+], [SH], [NH-], [cH-], [O+], [c-], [CH], [SH+], [CH2-], [OH+], [nH+], [SH2] - **1,942,081**

The final vocabulary contained 37 tokens (2 extra tokens were added, indicating <START> and <END>). The mean, median, and interquartile range of the number of tokens was 47.69, 47, and 17, respectively. We note that stereochemistry tokens were kept (this is not the case for REINVENT [4]).

In this work, we investigated LSTM [50] RNN, Decoder transformer [10, 11], and Mamba [12]. Given a vocabulary of 37, the model parameters were as follows:

1. RNN: 5,807,909 (based on REINVENT [4])
2. Decoder: 6,337,061 (based on recent work [27] that applied this model size and used a similar loss function to REINVENT)
3. Mamba: 5,265,920 (based on similar size to RNN)

The exact hyperparameters of each architecture are the default arguments in the codebase. Each training step consisted of a full pass through the dataset. The key pre-training parameters were:

1. Max training steps = 20
2. Seed = 0
3. Batch size = 512
4. Learning rate = 0.0001
5. Randomize [39] every batch of SMILES

The following model checkpoints were used:

1. RNN: Epoch 18, NLL = 34.61, Validity (10k) = 94.48%
2. Decoder: Epoch 20, NLL = 33.38, Validity (10k) = 96.04%
3. Mamba: Epoch 18, NLL = 32.21, Validity (10k) = 95.60%

1.7 Computational Requirements

In this paper all experiments were run on workstations equipped with either an NVIDIA RTX 3090 (24GB memory) or RTX A6000 (48GB memory) GPU and an AMD Ryzen 9 5900X 12-Core CPU. Running Saturn with the default 5.2M parameter Mamba model requires about 2.5GB GPU memory. The model can also be run on CPU, but for Mamba, this will notably increase the time for backpropagation due to the highly optimized GPU implementation we use from the original Mamba authors [12].

In this paper, all oracles used could be run on CPU. The default Saturn Mamba model requires 2.5-3.0 GB GPU memory and multiple parallel runs are feasible on most consumer-grade GPUs.

2 Part 1: Elucidating the Optimization Dynamics of Augmented Memory

In this section, we present results from all hyperparameter investigations for Saturn. In particular, we formulated four questions (each devoted to one subsection) which we answer with empirical results and discussion on the test experiment which has the following multi-parameter optimization (MPO) objective: molecular weight (MW) $<$ 350 Da, number of rings $\geq$ 2, and maximize topological polar surface area (tPSA).

Metrics. Following Guo et al. [2], the sample efficiency metrics are **Yield** and **Oracle Burden** (OB). Yield (Eq. 15) is the number of *unique* generated molecules above a reward threshold, T.

$$Yield = \sum_{g=1}^{G} \mathbb{I}[R(g) > T] \tag{15}$$

Oracle Burden (Eq. 16) is the number of oracle calls (c) required to generate N *unique* molecules above a reward threshold, T.

$$Oracle\ Burden = c \mid \sum_{g=1}^{G} \mathbb{I}[R(g) > T] = N \tag{16}$$

The Yield and OB metrics are used to assess sample efficiency at the 0.7 reward threshold. In all tables, the number after OB parentheses is the number of successful replicates out of 10. All metrics other than IntDiv1 [51] are rounded to the nearest integer. The IntDiv1 metric used Morgan fingerprints with radius 2 and 2048 bits. All individual experiments were run across 10 seeds (0-9 inclusive) and with a 1,000 oracle budget. All experiments were run sequentially on a workstation equipped with an NVIDIA RTX 3090 GPU and AMD Ryzen 9 5900X 12-Core CPU.

2.1 Understanding the Limits of Augmented Memory

Augmented Memory [1] improves sample efficiency by repeated learning on the high reward SMILES stored in the replay buffer (referred to as Buffer from here on). For completeness, we first describe *how* repeated learning can be achieved via data augmentation. SMILES are string representations resulting from performing a depth-first search (DFS) on a molecular graph (as is done in RDKit). Depending on the starting node (atom in the molecule), a different SMILES representation results from DFS. In Augmented Memory, SMILES augmentation is performed by shuffling the atom order and yielding different SMILES representations of the *same* molecular graph. This is useful as data augmentation because all of these SMILES representations map to the same molecule, yet their sequence likelihoods are different. Since they map to the same molecule, the same reward can be assigned to all augmented SMILES.

In the original work, ablation experiments showed that updating the Agent with *only* the Buffer resulted in minimal difference. This suggests that a viable way to exploiting the gains from Augmented Memory is to simply have *new* examples of high reward SMILES being added to the Buffer. In the original work, the number of augmentation rounds was capped at two to mitigate mode collapse. In this work, we assume *near deterministic* rewards and use caching to handle repeated generations. Under this assumption, our hypothesis in this subsection is: as long as unique high reward SMILES are generated, increasing augmentation rounds can further improve sample efficiency. Correspondingly, we perform a grid search using Augmented Memory's default generator architecture (LSTM [50] RNN) and vary the batch size (64, 32, 16, 8) and augmentation rounds (0-20 inclusive except 1) where 0 augmentation rounds is equivalent to REINVENT [3, 4]. The results are shown in Tables S1, S2, S3, and S4.

Increasing augmentation rounds:

1. Decreases diversity, as expected.

2. Increases the number of repeated SMILES.

Decreasing batch size:

1. Monotonically improves sample efficiency (though not always significant at the 95% confidence level via t-test).

2. Benefits Augmented memory more than REINVENT (0 augmentation rounds).

3. Increases the number of repeated SMILES.

4. Increases variance, as expected (since the expected reward is being approximated with a smaller batch size so it is more noisy).

5. Decreases diversity.

Taking these observations together, increasing augmentation rounds and decreasing batch size *can* trade-off diversity for sample efficiency (inconsistently and with higher variance).

Table S1: RNN batch size 64 augmentation rounds sweep.

Model	Aug. Rounds	Yield	IntDiv1	Scaffolds	OB 1	OB 10	OB 100	Repeats
RNN	0	0±0	—	0±0	584±251 (5)	Failed (0)	Failed (0)	1±1
RNN	2	15±9	0.775±0.073	15±9	644±173 (10)	941±58 (8)	Failed (0)	0±0
RNN	3	33±42	0.788±0.043	32±40	613±96 (10)	927±128 (9)	993±0 (1)	0±0
RNN	4	32±16	0.813±0.024	31±16	527±198 (10)	880±90 (10)	Failed (0)	0±0
RNN	5	40±14	0.812±0.023	39±13	459±177 (10)	862±68 (10)	Failed (0)	0±0
RNN	6	41±32	0.805±0.032	39±28	492±184 (10)	852±99 (9)	1041±0 (1)	0±0
RNN	7	47±25	0.814±0.019	46±24	543±188 (10)	842±93 (10)	1055±0 (1)	0±0
RNN	8	28±16	0.801±0.032	27±16	557±173 (10)	912±82 (9)	Failed (0)	0±0
RNN	9	21±13	0.742±0.124	21±13	596±215 (10)	918±61 (8)	Failed (0)	1±2
RNN	10	27±18	0.796±0.046	27±18	511±266 (10)	859±65 (8)	Failed (0)	0±0
RNN	11	20±14	0.749±0.115	20±14	611±235 (10)	938±85 (8)	Failed (0)	1±2
RNN	12	48±18	0.813±0.022	46±18	468±206 (10)	851±55 (10)	Failed (0)	1±1
RNN	13	57±43	0.808±0.027	54±39	446±213 (10)	822±144 (10)	952±0 (1)	1±2
RNN	14	33±13	0.801±0.024	32±13	587±175 (10)	884±79 (10)	Failed (0)	1±1
RNN	15	47±32	0.797±0.037	46±32	532±196 (10)	836±122 (10)	1052±0 (1)	2±2
RNN	16	34±32	0.783±0.026	33±30	647±208 (10)	918±97 (10)	1034±0 (1)	3±4
RNN	17	31±29	0.769±0.06	30±29	645±176 (10)	870±99 (7)	Failed (0)	3±4
RNN	18	35±28	0.774±0.035	32±24	673±125 (10)	898±88 (8)	1053±0 (1)	7±5
RNN	19	43±41	0.781±0.034	40±36	659±183 (10)	875±111 (8)	949±0 (1)	7±9
RNN	20	51±29	0.792±0.03	48±28	583±187 (10)	837±133 (10)	1056±0 (1)	3±2

Table S2: RNN batch size 32 augmentation rounds sweep.

Model	Aug. Rounds	Yield	IntDiv1	Scaffolds	OB 1	OB 10	OB 100	Repeats
RNN	0	0±0	—	0±0	798±101 (5)	Failed (0)	Failed (0)	1±1
RNN	2	43±25	0.825±0.029	42±24	608±151 (10)	844±90 (9)	Failed (0)	0±0
RNN	3	52±34	0.810±0.059	51±32	522±141 (10)	789±100 (9)	1018±0 (2)	0±1
RNN	4	87±33	0.820±0.018	83±31	466±120 (10)	740±77 (10)	987±30 (4)	1±3
RNN	5	98±57	0.817±0.027	89±50	408±184 (10)	714±136 (10)	915±20 (4)	1±2
RNN	6	76±50	0.808±0.028	71±43	476±159 (10)	783±99 (10)	927±30 (2)	1±3
RNN	7	78±40	0.805±0.027	72±40	478±90 (10)	760±70 (10)	942±26 (2)	3±7
RNN	8	89±72	0.798±0.036	78±58	529±165 (10)	767±146 (10)	899±48 (3)	9±13
RNN	9	57±52	0.781±0.046	50±42	608±186 (10)	811±143 (9)	977±36 (3)	5±4
RNN	10	90±65	0.788±0.031	82±55	549±158 (10)	769±142 (10)	977±66 (5)	9±14
RNN	11	60±43	0.755±0.105	57±43	593±207 (10)	781±83 (8)	969±52 (2)	2±2
RNN	12	103±83	0.790±0.021	90±72	534±168 (10)	763±158 (10)	930±105 (4)	10±23
RNN	13	72±57	0.749±0.065	62±52	578±155 (10)	765±134 (8)	958±54 (3)	12±9
RNN	14	95±55	0.779±0.027	83±47	463±173 (10)	758±110 (10)	964±28 (5)	16±15
RNN	15	74±60	0.784±0.036	66±52	554±92 (10)	820±124 (10)	963±54 (4)	22±20
RNN	16	84±60	0.758±0.07	70±44	544±209 (10)	768±105 (9)	957±42 (5)	17±19
RNN	17	112±74	0.765±0.067	96±56	474±131 (10)	729±105 (10)	908±96 (4)	21±21
RNN	18	77±49	0.774±0.039	67±43	533±100 (10)	779±102 (10)	927±12 (2)	35±32
RNN	19	84±56	0.749±0.037	68±50	535±181 (10)	788±127 (10)	951±61 (3)	33±44
RNN	20	76±77	0.717±0.094	64±61	653±200 (10)	810±121 (9)	919±76 (3)	56±64

Table S3: RNN batch size 16 augmentation rounds sweep.

Model	Aug. Rounds	Yield	IntDiv1	Scaffolds	OB 1	OB 10	OB 100	Repeats
RNN	0	8±9	0.700±0.126	8±9	546±263 (8)	837±144 (3)	Failed (0)	1±1
RNN	2	86±40	0.819±0.026	82±38	409±158 (10)	709±86 (10)	907±14 (2)	2±4
RNN	3	103±47	0.831±0.027	100±44	406±157 (10)	706±98 (10)	942±45 (5)	2±3
RNN	4	90±62	0.828±0.017	83±53	440±152 (10)	741±102 (10)	916±76 (3)	1±1
RNN	5	107±58	0.814±0.036	101±54	480±118 (10)	721±109 (10)	916±53 (4)	7±7
RNN	6	121±80	0.791±0.040	107±68	493±214 (10)	713±156 (10)	895±107 (5)	12±11
RNN	7	144±107	0.776±0.026	117±86	467±186 (10)	684±136 (10)	871±116 (6)	38±82
RNN	8	120±95	0.734±0.128	104±85	481±288 (10)	653±145 (8)	854±54 (5)	18±28
RNN	9	141±104	0.783±0.048	112±72	453±211 (10)	654±154 (9)	871±104 (6)	59±95
RNN	10	106±76	0.760±0.056	84±63	510±201 (10)	733±122 (9)	913±64 (5)	43±47
RNN	11	120±105	0.764±0.032	95±81	500±220 (10)	741±199 (10)	829±99 (4)	42±37
RNN	12	171±140	0.769±0.028	124±109	389±209 (10)	662±186 (10)	774±128 (5)	39±30
RNN	13	133±106	0.767±0.038	106±93	510±186 (10)	690±162 (10)	826±131 (4)	83±88
RNN	14	166±130	0.769±0.045	129±93	413±237 (10)	659±195 (10)	777±94 (5)	93±69
RNN	15	154±89	0.732±0.064	127±78	504±162 (10)	647±124 (9)	861±59 (7)	94±75
RNN	16	156±155	0.716±0.094	109±109	517±196 (10)	682±202 (9)	838±182 (6)	143±120
RNN	17	141±82	0.737±0.059	98±49	444±181 (10)	696±128 (10)	894±71 (7)	198±163
RNN	18	189±136	0.727±0.044	152±119	469±212 (10)	657±174 (10)	832±141 (7)	247±210
RNN	19	162±121	0.654±0.165	119±98	507±257 (10)	625±137 (8)	836±109 (7)	210±128
RNN	20	139±110	0.732±0.045	91±67	492±188 (10)	720±157 (10)	847±110 (5)	262±179

Table S4: RNN batch size 8 augmentation rounds sweep.

Model	Aug. Rounds	Yield	IntDiv1	Scaffolds	OB 1	OB 10	OB 100	Repeats
RNN	0	21±21	0.645±0.133	17±18	481±291 (10)	826±95 (6)	Failed (0)	16±15
RNN	2	136±100	0.807±0.028	113±73	428±169 (10)	665±159 (10)	849±113 (5)	8±9
RNN	3	143±97	0.793±0.037	131±85	395±169 (10)	667±126 (10)	863±109 (6)	27±33
RNN	4	152±115	0.785±0.022	129±96	379±212 (10)	680±179 (10)	865±124 (7)	44±47
RNN	5	164±84	0.786±0.038	123±56	350±158 (10)	643±121 (10)	876±81 (8)	40±41
RNN	6	224±104	0.790±0.041	181±79	352±176 (10)	584±159 (10)	782±56 (8)	49±40
RNN	7	185±111	0.751±0.070	151±96	435±224 (10)	608±127 (9)	814±86 (7)	116±119
RNN	8	159±128	0.775±0.050	128±114	460±195 (10)	646±145 (9)	858±140 (7)	105±77
RNN	9	198±164	0.732±0.072	151±121	451±227 (10)	641±158 (9)	782±168 (6)	285±396
RNN	10	139±127	0.728±0.078	100±73	512±212 (8)	702±124 (7)	867±145 (4)	112±61
RNN	11	205±173	0.753±0.062	151±120	444±267 (10)	652±234 (10)	737±167 (6)	254±320
RNN	12	261±165	0.762±0.057	211±135	320±246 (10)	579±210 (10)	775±168 (9)	518±760
RNN	13	231±198	0.753±0.061	155±101	444±184 (9)	601±235 (9)	790±214 (8)	351±289
RNN	14	158±103	0.718±0.091	108±60	526±208 (10)	681±127 (9)	845±80 (6)	374±308
RNN	15	221±128	0.731±0.043	150±129	439±196 (10)	618±168 (10)	826±153 (9)	461±292
RNN	16	196±145	0.725±0.043	136±101	470±228 (10)	683±198 (10)	813±141 (7)	694±495
RNN	17	258±130	0.689±0.119	193±94	467±210 (10)	576±139 (9)	787±115 (9)	796±600
RNN	18	253±114	0.727±0.047	195±98	394±175 (10)	605±124 (10)	764±82 (8)	1112±974
RNN	19	268±159	0.714±0.052	204±132	418±161 (10)	579±167 (10)	745±153 (8)	817±811
RNN	20	292±153	0.713±0.039	220±121	397±205 (10)	574±188 (10)	776±173 (10)	1406±1391

2.2 Do Architectures Differ in Behavior?

RNNs essentially solve the validity, uniqueness, and novelty metrics [20, 21] and can learn molecular distributions well [22] for small molecule design. In this subsection, we extend Augmented Memory to Decoder transformer [10, 11] and Mamba [12] to investigate the RL dynamics and empirically investigate potential benefits. Our hypothesis is that since self-attention [10] and selective scanning [12] *can* capture different structural elements [33] (via focusing on different aspects of the sequence), benefits *may* arise from capturing and focusing on favorable moieties. Our analysis is focused solely on sample efficiency metrics and not validity, uniqueness, and novelty.

Similar to the previous subsection, we perform a grid-search over batch size (64, 32, 16, 8) and augmentation rounds (0-20 inclusive except 1). As the results for RNN were presented in the previous subsection, this subsection only shows Decoder and Mamba results (Tables S5, S6, S7, S8, S9, S10, S11, and S12).

The following observations are similar to RNN. Increasing augmentation rounds:

1. Decreases diversity, as expected.
2. Increases the number of repeated SMILES.

Decreasing batch size:

1. Monotonically improves sample efficiency (though not always significant at the 95% confidence level via t-test).
2. Benefits Augmented memory more than REINVENT (0 augmentation rounds).
3. Increases the number of repeated SMILES.
4. Increases variance, as expected (since the expected reward is being approximated with a smaller batch size so it is more noisy).
5. Decreases diversity.

The following observations contrast RNN with Decoder and Mamba:

1. Mamba > Decoder > RNN in terms of NLL convergence (end of Supplementary Information 1.6).

2. Propensity to generate repeated SMILES follows the same trend and is further supported with the IntDiv1 generally being lower than RNN for the same number of augmentation rounds across all batch sizes.

3. Mamba notably generates many repeated SMILES but sample efficiency improves, thus it is not detrimental under the assumption that the reward is *near deterministic* and oracle evaluations are cached.

4. In general, Decoder does not outperform RNN

Taking these observations together and exactly like RNN results, increasing augmentation rounds and decreasing batch size *can* trade-off diversity for sample efficiency (inconsistently and with higher variance). However, of difference, is that Mamba at lower batch sizes (particularly 16) and relatively high augmentation rounds (10) improves sample efficiency in a statistically significant way (at the 95% confidence level via t-test).

Further Note 1. We have observed that with low batch size and high augmentation rounds, Mamba can temporarily lose generative ability (or generate the same molecules). Specifically, the validity of the generated batch can be 0. Sampling a new batch can recover this validity but we have observed in some cases, that validity can be 0 for an extensive period (no valid molecules were generated in the sampled batch). We observed this scenario a few times during development (over 5,000 individual experiment runs), occurring with a batch size of 8 and augmentation rounds > 15. We speculate the reason is extreme mode collapse to a chemical space where syntax is sensitive. This process often recovers where the model, by chance, generates valid molecules again and steers the model back to "valid molecule space". In practice, with high-fidelity oracles, one would checkpoint models frequently (even every epoch), as each batch of oracle evaluation would be costly. Alternatively, as all high reward SMILES (so far) generated can be pre-emptively saved. It would be feasible to even start a new run with these SMILES seeded in the replay buffer, akin to inception in REINVENT [3] (transfer learning would work too). This would kick-start the optimization and already guide the Agent to this chemical space, preventing optimization progress from being completely "lost". Losing validity during generation is also a previously observed behavior [84] and was handled by resetting the model state to a saved checkpoint. This behavior is likely also highly dependent on the objective function which affects the optimization landscape. We write this note for full transparency into all the behavior we have observed in our development experiments.

Further Note 2. In this work, repeat generated SMILES (already generated in a previous epoch) are not evaluated by the oracle as the reward is retrieved from a cache. This retrieval action is checked by canonicalizing the generated SMILES and querying whether it is present in the reward cache. We have observed that this is sometimes not the case, and the final generated set can contain duplicates. Across over 2,000 individual runs, this occurs mostly for small batch sizes (16 and 8) with high augmentation rounds (> 15). This is unsurprising as these settings lead to greater exploitation and hence propensity to generate the same molecules. Quantitatively amongst these runs, approximately 50% contained no duplicates. 77% of runs contained 1-3 duplicates, 85% of runs contained 1-5 duplicates, 95% of runs contained 1-10 duplicates, and 99.5% of runs contained 1-20 duplicates.

Since the pre-print version of this work, we identified a potential source of this behavior: As an

example, if a sampled batch contains two SMILES forms of a given molecule, and this molecule has not been generated before, both SMILES forms could be considered "new". Consequently, both SMILES copies would be stored in the final generated set.

Table S5: Decoder batch size 64 augmentation rounds sweep.

Model	Aug. Rounds	Yield	IntDiv1	Scaffolds	OB 1	OB 10	OB 100	Repeats
Decoder	0	1±1	0.548±0.129	1±1	691±266 (6)	Failed (0)	Failed (0)	2±1
Decoder	2	26±19	0.800±0.061	26±18	524±128 (10)	868±76 (8)	Failed (0)	0±0
Decoder	3	37±24	0.801±0.031	36±23	629±154 (10)	849±85 (9)	Failed (0)	0±0
Decoder	4	49±38	0.797±0.055	48±37	590±142 (10)	851±89 (9)	984±0 (1)	0±0
Decoder	5	63±35	0.821±0.014	62±35	545±136 (10)	814±84 (10)	997±21 (2)	1±1
Decoder	6	43±34	0.794±0.033	40±32	649±155 (10)	881±127 (10)	1045±0 (1)	2±4
Decoder	7	42±29	0.800±0.039	41±29	585±175 (10)	859±116 (9)	1042±0 (1)	4±3
Decoder	8	22±28	0.719±0.119	21±28	717±157 (10)	939±104 (7)	1051±0 (1)	6±6
Decoder	9	23±22	0.704±0.156	19±16	618±233 (10)	889±92 (7)	Failed (0)	10±5
Decoder	10	43±48	0.768±0.056	41±47	643±110 (10)	788±104 (6)	980±0 (1)	10±7
Decoder	11	36±45	0.756±0.068	34±44	698±116 (10)	881±108 (8)	891±0 (1)	9±7
Decoder	12	47±28	0.795±0.02	43±27	609±101 (9)	862±74 (9)	1046±0 (1)	16±9
Decoder	13	66±66	0.727±0.109	56±54	641±216 (10)	788±148 (8)	975±75 (2)	37±25
Decoder	14	38±37	0.696±0.139	33±34	679±169 (10)	868±104 (7)	1004±0 (1)	46±28
Decoder	15	38±56	0.671±0.100	25±32	668±241 (9)	809±159 (5)	977±9 (2)	56±28
Decoder	16	33±41	0.716±0.084	25±29	572±221 (10)	900±122 (8)	984±0 (1)	78±38
Decoder	17	50±48	0.707±0.091	37±30	595±250 (10)	797±86 (7)	1007±34 (2)	91±42
Decoder	18	30±36	0.732±0.049	26±32	701±135 (8)	886±101 (6)	1025±0 (1)	124±41
Decoder	19	35±31	0.715±0.056	28±21	640±240 (10)	852±155 (8)	1031±0 (1)	159±64
Decoder	20	51±51	0.733±0.047	39±38	585±277 (9)	862±136 (8)	984±49 (2)	172±69

Table S6: Decoder batch size 32 augmentation rounds sweep.

Model	Aug. Rounds	Yield	IntDiv1	Scaffolds	OB 1	OB 10	OB 100	Repeats
Decoder	0	4±4	0.710±0.023	4±4	647±232 (6)	982±39 (2)	Failed (0)	10±13
Decoder	2	45±23	0.813±0.021	43±22	557±174 (10)	844±91 (10)	Failed (0)	1±1
Decoder	3	66±44	0.801±0.033	63±43	515±146 (10)	779±70 (9)	918±0 (1)	1±1
Decoder	4	111±88	0.791±0.017	100±80	476±131 (10)	726±133 (10)	908±81 (5)	3±3
Decoder	5	94±70	0.791±0.043	81±53	489±155 (10)	753±112 (9)	897±63 (3)	3±2
Decoder	6	94±66	0.770±0.075	82±60	476±204 (10)	696±126 (9)	921±52 (4)	11±6
Decoder	7	117±87	0.730±0.084	105±84	473±270 (10)	659±99 (8)	936±93 (6)	54±84
Decoder	8	78±69	0.776±0.032	67±52	519±204 (10)	797±147 (10)	926±94 (3)	35±13
Decoder	9	59±35	0.767±0.032	51±32	575±76 (10)	856±83 (10)	968±0 (1)	44±33
Decoder	10	91±75	0.742±0.065	68±52	492±176 (9)	769±121 (9)	879±66 (2)	77±56
Decoder	11	70±46	0.739±0.059	57±36	559±128 (10)	811±96 (10)	974±6 (3)	84±45
Decoder	12	114±58	0.730±0.041	82±45	559±177 (10)	715±59 (9)	942±48 (6)	124±81
Decoder	13	93±83	0.741±0.064	77±68	598±114 (10)	788±129 (9)	874±34 (3)	146±76
Decoder	14	147±112	0.752±0.064	109±84	486±147 (9)	694±152 (9)	791±37 (4)	257±269
Decoder	15	140±100	0.718±0.085	111±78	516±256 (10)	676±143 (9)	916±106 (7)	222±128
Decoder	16	130±142	0.709±0.045	82±66	552±177 (10)	772±164 (10)	851±173 (4)	405±272
Decoder	17	130±125	0.720±0.075	95±89	624±209 (10)	771±186 (10)	841±137 (4)	444±265
Decoder	18	153±165	0.718±0.055	110±130	565±191 (10)	718±197 (9)	668±81 (3)	544±503
Decoder	19	149±94	0.686±0.055	104±69	547±215 (10)	731±113 (9)	897±83 (7)	594±172
Decoder	20	137±135	0.693±0.046	78±56	555±200 (9)	740±181 (9)	855±145 (5)	514±399

Table S7: Decoder batch size 16 augmentation rounds sweep.

Model	Aug. Rounds	Yield	IntDiv1	Scaffolds	OB 1	OB 10	OB 100	Repeats
Decoder	0	2±3	0.55±0.1	2±2	810±93 (7)	983±0 (1)	Failed (0)	78±25
Decoder	2	66±50	0.796±0.037	59±41	602±158 (10)	799±106 (9)	921±3 (2)	8±7
Decoder	3	84±66	0.77±0.037	64±44	536±170 (10)	769±122 (9)	919±44 (4)	28±24
Decoder	4	71±44	0.74±0.102	62±41	632±118 (10)	780±82 (9)	977±36 (3)	22±12
Decoder	5	154±93	0.748±0.052	122±70	439±151 (10)	679±128 (10)	907±92 (8)	90±90
Decoder	6	116±94	0.748±0.039	86±64	517±165 (10)	728±158 (10)	904±126 (5)	73±42
Decoder	7	108±85	0.747±0.051	71±50	510±222 (10)	740±127 (9)	868±48 (4)	126±63
Decoder	8	108±94	0.708±0.109	72±57	538±164 (10)	742±116 (9)	887±87 (4)	150±72
Decoder	9	78±83	0.687±0.116	51±55	614±244 (10)	790±150 (8)	890±62 (3)	242±139
Decoder	10	120±128	0.691±0.042	74±73	663±170 (9)	768±169 (8)	805±65 (4)	344±218
Decoder	11	146±134	0.727±0.038	110±100	609±169 (9)	725±166 (9)	829±132 (5)	389±199
Decoder	12	119±127	0.704±0.047	76±68	624±185 (9)	779±176 (9)	828±110 (4)	363±256
Decoder	13	183±177	0.696±0.031	97±80	484±227 (9)	671±216 (9)	753±144 (5)	498±412
Decoder	14	146±111	0.673±0.055	88±60	572±240 (10)	737±162 (9)	850±87 (6)	702±387
Decoder	15	146±100	0.64±0.123	108±79	623±141 (10)	772±150 (10)	867±70 (6)	774±414
Decoder	16	209±173	0.688±0.043	155±130	530±124 (9)	654±161 (9)	813±170 (7)	1369±777
Decoder	17	190±168	0.662±0.109	154±149	571±207 (10)	674±179 (9)	746±162 (5)	1096±883
Decoder	18	226±138	0.668±0.052	174±115	550±156 (10)	646±131 (9)	802±118 (8)	1540±986
Decoder	19	232±154	0.648±0.07	168±96	564±152 (10)	681±161 (10)	781±147 (7)	1693±1165
Decoder	20	258±200	0.636±0.077	166±103	448±223 (9)	589±179 (8)	763±177 (8)	1741±1020

Table S8: Decoder batch size 8 augmentation rounds sweep.

Model	Aug. Rounds	Yield	IntDiv1	Scaffolds	OB 1	OB 10	OB 100	Repeats
Decoder	0	57±64	0.621±0.222	37±36	554±137 (9)	766±178 (7)	912±52 (3)	368±164
Decoder	2	120±76	0.745±0.055	97±59	497±207 (10)	667±110 (8)	913±62 (7)	39±22
Decoder	3	93±60	0.73±0.06	74±45	530±166 (10)	759±87 (9)	918±22 (4)	128±82
Decoder	4	111±49	0.741±0.036	79±34	467±170 (10)	737±101 (10)	950±32 (7)	173±81
Decoder	5	79±82	0.724±0.044	59±54	609±123 (8)	805±101 (8)	901±72 (3)	283±179
Decoder	6	138±112	0.72±0.062	96±78	608±162 (10)	737±138 (9)	843±81 (5)	400±222
Decoder	7	197±165	0.688±0.064	149±131	502±287 (10)	684±237 (10)	758±112 (6)	820±1051
Decoder	8	219±179	0.68±0.063	132±120	475±201 (8)	581±127 (7)	763±136 (7)	840±900
Decoder	9	194±144	0.651±0.049	153±118	496±157 (8)	627±149 (8)	791±109 (7)	1059±864
Decoder	10	183±200	0.684±0.055	130±130	571±201 (9)	654±217 (8)	789±205 (6)	944±597
Decoder	11	141±123	0.581±0.166	96±84	617±198 (9)	662±142 (7)	801±97 (5)	1715±1380
Decoder	12	133±196	0.574±0.149	92±135	665±291 (9)	699±268 (7)	664±209 (3)	1604±1130
Decoder	13	331±151	0.664±0.095	271±143	418±230 (10)	503±88 (9)	711±107 (9)	2030±1408
Decoder	14	164±152	0.602±0.06	125±109	620±257 (9)	714±194 (8)	825±133 (6)	2628±1665
Decoder	15	281±242	0.661±0.054	230±185	496±243 (9)	589±251 (9)	663±201 (7)	2482±1515
Decoder	16	213±191	0.58±0.143	180±176	512±245 (9)	596±223 (8)	730±186 (6)	3113±2436
Decoder	17	252±186	0.622±0.072	203±167	614±231 (10)	615±169 (8)	735±139 (7)	3278±1894
Decoder	18	81±113	0.595±0.064	69±97	630±232 (7)	759±209 (7)	862±102 (3)	2811±2415
Decoder	19	136±171	0.611±0.062	119±154	645±195 (7)	708±180 (6)	771±142 (4)	2886±2066
Decoder	20	98±139	0.54±0.075	91±136	736±195 (7)	785±160 (6)	813±140 (3)	3190±2113

Table S9: Mamba batch size 64 augmentation rounds sweep.

Model	Aug. Rounds	Yield	IntDiv1	Scaffolds	OB 1	OB 10	OB 100	Repeats
Mamba	0	0±0	—	0±0	946±41 (2)	Failed (0)	Failed (0)	0±1
Mamba	2	2±1	0.580±0.086	2±1	817±244 (10)	Failed (0)	Failed (0)	0±0
Mamba	3	9±6	0.734±0.068	9±6	659±234 (9)	942±34 (4)	Failed (0)	1±1
Mamba	4	6±3	0.672±0.114	6±3	652±297 (10)	1040±7 (2)	Failed (0)	2±2
Mamba	5	9±5	0.697±0.113	9±5	640±210 (10)	995±30 (5)	Failed (0)	3±3
Mamba	6	17±11	0.770±0.041	17±11	656±119 (10)	960±90 (9)	Failed (0)	6±4
Mamba	7	19±6	0.769±0.027	18±6	623±152 (10)	957±65 (9)	Failed (0)	7±3
Mamba	8	29±15	0.786±0.035	27±15	545±176 (10)	917±82 (10)	Failed (0)	12±8
Mamba	9	21±10	0.755±0.075	20±10	585±192 (10)	938±57 (9)	Failed (0)	26±23
Mamba	10	34±22	0.785±0.028	28±15	486±176 (10)	884±91 (10)	Failed (0)	30±21
Mamba	11	18±8	0.757±0.044	17±7	550±203 (10)	937±31 (8)	Failed (0)	37±21
Mamba	12	22±17	0.727±0.051	20±15	629±234 (10)	876±53 (6)	Failed (0)	72±68
Mamba	13	33±33	0.739±0.090	29±28	561±222 (10)	915±120 (10)	1020±0 (1)	62±28
Mamba	14	47±39	0.701±0.138	30±15	540±242 (10)	839±94 (8)	980±0 (1)	127±56
Mamba	15	60±88	0.725±0.117	31±17	585±225 (10)	866±143 (10)	726±0 (1)	136±112
Mamba	16	46±40	0.661±0.170	29±22	614±193 (10)	865±104 (9)	978±33 (2)	199±89
Mamba	17	43±24	0.727±0.054	30±13	538±185 (10)	866±101 (10)	Failed (0)	174±77
Mamba	18	51±42	0.732±0.056	40±32	621±219 (10)	838±111 (9)	995±34 (2)	262±99
Mamba	19	49±40	0.723±0.048	36±25	633±218 (10)	829±123 (8)	975±0 (1)	241±73
Mamba	20	77±68	0.695±0.088	46±32	549±241 (9)	771±146 (8)	940±76 (3)	385±180

Table S10: Mamba batch size 32 augmentation rounds sweep.

Model	Aug. Rounds	Yield	IntDiv1	Scaffolds	OB 1	OB 10	OB 100	Repeats
Mamba	0	0±0	—	0±0	773±189 (4)	Failed (0)	Failed (0)	4±2
Mamba	2	12±7	0.744±0.060	12±7	644±199 (10)	933±29 (5)	Failed (0)	3±2
Mamba	3	16±9	0.759±0.050	15±9	640±158 (10)	912±45 (6)	Failed (0)	8±7
Mamba	4	30±15	0.797±0.029	29±15	579±140 (10)	879±86 (10)	Failed (0)	11±5
Mamba	5	38±23	0.718±0.151	35±21	695±159 (10)	833±83 (8)	Failed (0)	24±9
Mamba	6	44±37	0.770±0.044	41±34	564±145 (10)	861±110 (9)	1000±3 (2)	42±17
Mamba	7	52±43	0.750±0.047	46±37	539±174 (10)	848±123 (10)	996±11 (2)	68±28
Mamba	8	76±51	0.775±0.025	67±45	515±108 (10)	794±85 (10)	923±30 (2)	90±49
Mamba	9	64±47	0.755±0.083	53±38	546±143 (10)	808±116 (10)	959±45 (2)	140±106
Mamba	10	96±76	0.768±0.028	75±54	553±186 (10)	782±161 (10)	949±84 (5)	165±63
Mamba	11	87±60	0.732±0.045	62±40	592±218 (10)	741±105 (8)	936±31 (3)	303±152
Mamba	12	118±60	0.680±0.130	67±21	500±159 (10)	730±132 (10)	932±61 (6)	280±151
Mamba	13	92±60	0.742±0.082	74±43	578±226 (10)	771±98 (9)	940±39 (4)	353±104
Mamba	14	166±75	0.748±0.041	121±54	458±97 (10)	659±64 (10)	901±78 (8)	483±202
Mamba	15	139±94	0.755±0.033	106±72	456±141 (10)	740±127 (10)	847±54 (5)	488±167
Mamba	16	136±75	0.740±0.039	97±54	571±131 (10)	742±119 (10)	899±50 (6)	769±354
Mamba	17	186±88	0.696±0.058	138±83	510±103 (10)	683±88 (10)	871±76 (8)	937±677
Mamba	18	214±87	0.723±0.059	169±81	540±113 (10)	672±88 (10)	862±84 (9)	1027±554
Mamba	19	242±109	0.686±0.041	184±104	493±133 (10)	661±116 (10)	819±109 (9)	1376±596
Mamba	20	187±78	0.706±0.038	152±67	557±101 (10)	714±80 (10)	892±79 (9)	1183±413

Table S11: Mamba batch size 16 augmentation rounds sweep.

Model	Aug. Rounds	Yield	IntDiv1	Scaffolds	OB 1	OB 10	OB 100	Repeats
Mamba	0	3±4	0.417±0.161	2±2	545±232 (7)	982±0 (1)	Failed (0)	91±32
Mamba	2	39±29	0.761±0.047	34±23	609±165 (10)	829±117 (9)	Failed (0)	46±31
Mamba	3	61±51	0.771±0.051	50±39	498±193 (10)	797±118 (9)	953±15 (3)	71±28
Mamba	4	52±33	0.779±0.031	42±23	581±102 (10)	817±112 (10)	970±0 (1)	139±59
Mamba	5	69±38	0.764±0.052	54±28	542±93 (10)	807±76 (10)	988±17 (3)	178±90
Mamba	6	138±46	0.759±0.039	110±42	456±89 (10)	693±75 (10)	919±36 (7)	286±137
Mamba	7	174±95	0.737±0.059	127±83	427±177 (10)	643±102 (10)	858±77 (7)	395±147
Mamba	8	209±95	0.751±0.030	137±60	461±151 (10)	617±135 (10)	817±71 (8)	482±214
Mamba	9	202±98	0.735±0.032	137±80	389±112 (10)	631±102 (10)	841±92 (8)	518±237
Mamba	10	306±57	0.714±0.035	206±34	387±148 (10)	555±66 (10)	761±58 (10)	1110±636
Mamba	11	306±92	0.716±0.039	237±85	403±136 (10)	554±93 (10)	761±100 (10)	1341±596
Mamba	12	266±100	0.723±0.041	199±83	392±126 (10)	590±100 (10)	806±111 (10)	1312±666
Mamba	13	327±108	0.722±0.043	258±101	428±111 (10)	549±111 (10)	741±116 (10)	1508±780
Mamba	14	318±109	0.695±0.061	246±117	416±164 (10)	535±148 (10)	736±123 (10)	1776±912
Mamba	15	284±74	0.691±0.052	219±42	442±67 (10)	584±87 (10)	785±82 (10)	2629±939
Mamba	16	293±112	0.672±0.053	209±77	483±145 (10)	570±136 (10)	767±130 (10)	2284±1011
Mamba	17	344±115	0.656±0.047	278±92	462±113 (10)	563±98 (10)	725±121 (10)	3512±1227
Mamba	18	281±155	0.640±0.082	216±125	464±174 (9)	595±155 (9)	730±93 (8)	2885±1344
Mamba	19	307±115	0.624±0.084	238±102	491±146 (10)	579±133 (10)	750±119 (10)	3318±1347
Mamba	20	352±69	0.673±0.046	294±61	403±102 (10)	525±81 (10)	714±79 (10)	3331±1454

Table S12: Mamba batch size 8 augmentation rounds sweep.

Model	Aug. Rounds	Yield	IntDiv1	Scaffolds	OB 1	OB 10	OB 100	Repeats
Mamba	0	3±2	0.43±0.133	2±1	498±322 (8)	Failed (0)	Failed (0)	940±234
Mamba	2	69±32	0.755±0.059	56±28	453±176 (10)	780±78 (10)	992±8 (2)	214±72
Mamba	3	156±113	0.745±0.035	109±70	452±221 (10)	659±143 (9)	792±83 (5)	282±120
Mamba	4	200±117	0.748±0.046	125±64	402±208 (10)	602±150 (10)	859±145 (9)	425±160
Mamba	5	240±102	0.719±0.062	195±102	429±191 (10)	596±136 (10)	805±108 (9)	1195±687
Mamba	6	298±167	0.706±0.052	212±122	405±190 (10)	557±197 (10)	736±170 (9)	1420±632
Mamba	7	328±116	0.662±0.107	246±112	332±142 (10)	489±131 (10)	727±124 (10)	1657±947
Mamba	8	356±142	0.671±0.029	304±119	380±158 (10)	514±144 (10)	699±167 (10)	2340±806
Mamba	9	359±135	0.682±0.054	298±115	439±140 (10)	536±161 (10)	663±102 (9)	2974±1394
Mamba	10	368±164	0.692±0.032	305±154	391±234 (10)	485±99 (9)	658±125 (9)	2829±1290
Mamba	11	321±148	0.636±0.048	280±137	415±154 (10)	561±153 (10)	720±145 (9)	3515±1592
Mamba	12	335±148	0.637±0.055	285±148	425±162 (10)	564±178 (10)	687±135 (9)	4060±1694
Mamba	13	260±158	0.579±0.121	213±139	505±168 (10)	602±141 (9)	744±130 (8)	3691±1790
Mamba	14	290±120	0.608±0.047	235±89	463±213 (10)	583±150 (10)	765±127 (10)	4505±1968
Mamba	15	343±157	0.621±0.069	317±149	367±140 (10)	534±159 (10)	706±166 (10)	4196±1064
Mamba	16	320±214	0.61±0.095	293±199	450±210 (10)	560±241 (9)	602±141 (7)	5035±1995
Mamba	17	233±131	0.611±0.059	219±131	552±165 (10)	665±147 (10)	806±130 (9)	3728±1946
Mamba	18	270±205	0.617±0.061	256±200	516±155 (10)	628±191 (10)	705±201 (7)	5378±2020
Mamba	19	168±164	0.632±0.070	139±121	468±221 (8)	604±233 (8)	805±193 (6)	4740±2181
Mamba	20	256±196	0.539±0.190	245±192	462±225 (9)	531±233 (8)	642±156 (7)	4476±2383

2.3 Are Increased Augmentation Rounds Tolerable with Beam Enumeration?

Beam Enumeration [2] extracts the most probable substructures for self-conditioned generation and has been shown to be synergistic with Augmented Memory [1] such that the Yield and OB improve. In the original work, the oracle budget in the experiments was 5,000. In this work, we are interested in minimizing the oracle budget and all experiments thus far use a 1,000 oracle budget. Beam Enumeration has a *Patience* criterion which controls when substructures are extracted: only when the average reward improves for *Patience* number of successive epochs. Since we are operating at a much lower oracle budget, it is especially unclear whether Beam Enumeration can still benefit sample efficiency (we note that the explainability aspect is still applicable). In the original work, a batch size of 64 was used and a Patience of 5. Under these parameters, the earliest that Beam Enumeration can execute is 320/1000 oracle calls, which is almost 1/3 the budget already. Moreover, Beam Enumeration decreases diversity and decreasing batch size and increasing augmentation rounds also decreases diversity. *Too much* decrease in diversity may be detrimental even with oracle caching. In this subsection, we systematically study the effect of Beam Enumeration when used in conjunction with decreasing batch size and augmentation rounds in a series of hypotheses.

Based on observations from batch size and augmentation rounds grid-searches, the following design decisions were made in this subsection:

1. Augmentation rounds capped at 5 as diversity generally decreases more substantially past this point. Beam Enumeration itself will decrease diversity, so this is a preemptive measure against detrimental diversity-induced mode collapse.

2. Investigate batch sizes of 64 and 32. Since Beam Enumeration executes on improved reward over successive epochs, lower batch sizes would likely increase performance variance too much.

3. Focus only on RNN model as experiments will be the fastest (less repeated SMILES). If benefits are observed, move to Decoder and Mamba models. For clarity, repeated SMILES are not detrimental, as we have shown in the previous subsections but they add some wall

time (this is insignificant when compared to expensive oracles).

4. Beam Enumeration can pool improbable substructures. There is a Patience Limit denoting the number epochs permitted where the entire generated batch is filtered. This limit was 100,000 in this work. This does not add that much wall time and surpassing the limit is not indicative of the experiment failing. However, we enforce this upper bound in case it occurs (seldom) to manage wall times since we are performing grid searches.

5. Use Minimum Structure Size = 15, unless otherwise stated. Enforcing larger substructure extraction was found to improve sample efficiency in the original work [2]

2.3.1 Hypothesis 1

Beam Enumeration's Patience parameter is dependent on the mean reward of the sampled batch. With lower batch sizes, variance increases, such that executing Beam Enumeration may be *too variable*.

Proposed solution. Increase Beam Enumeration's default Patience (5) to mitigate lower batch size variance. We note that increasing Patience means that more of the oracle budget needs to be consumed before Beam Enumeration executes for the first time. First explore Batch sizes = [64, 32].

Observations. Across batch sizes = [64, 32] and all Patience = [5, 6, 7, 8, 9, 10], sample efficiency does not improve over just using Augmented Memory and IntDiv1 (diversity) also decreases by at least 0.100 (Tables S13 and S14). Adding Beam Enumeration neither increases Yield nor diversity. In the batch size 32 setting, even when the Yield can match just using Augmented Memory, the diversity continues to decreases (Table S14).

Table S13: Beam Enumeration batch size 64 with Structure and Minimum Size 15. Filter Limit is the number of times that no SMILES contained the pool substructure in 100,000 generation epochs. Patience N/A indicates just Augmented Memory and no Beam Enumeration.

Patience	Aug. Rounds	Yield	IntDiv1	Scaffolds	OB 1	OB 10	OB 100	Repeats	Filter Limit
N/A	0	0±0	—	0±0	584±251 (5)	Failed	Failed	1±1	N/A
N/A	2	15±9	0.775±0.073	15±9	644±173 (10)	941±58 (8)	Failed	0±0	N/A
N/A	3	33±42	0.788±0.043	32±40	613±96 (10)	927±128 (9)	993±0 (1)	0±0	N/A
N/A	4	32±16	0.813±0.024	31±16	527±198 (10)	880±90 (10)	Failed	0±0	N/A
N/A	5	40±14	0.812±0.023	39±13	459±177 (10)	862±68 (10)	Failed	0±0	N/A
5	0	2±2	—	2±2	687±232 (7)	Failed	Failed	17±21	0
5	2	29±68	0.688±0.044	22±48	555±185 (8)	887±182 (4)	866±0 (1)	15±27	1
5	3	110±75	0.754±0.024	81±52	488±79 (10)	711±99 (10)	902±79 (4)	20±21	0
5	4	86±82	0.702±0.045	58±53	504±205 (10)	739±193 (9)	912±76 (3)	14±15	0
5	5	94±41	0.745±0.027	68±30	436±167 (10)	739±88 (10)	970±30 (4)	15±17	0
6	0	2±3	—	2±2	581±205 (7)	958±0 (1)	Failed	25±29	0
6	2	20±20	0.619±0.168	16±15	659±226 (10)	809±27 (4)	Failed	9±10	0
6	3	82±84	0.730±0.039	52±44	520±84 (10)	777±134 (10)	863±131	19±26	0
6	4	83±91	0.723±0.074	62±62	508±233 (9)	737±130 (8)	874±93	19±21	0
6	5	84±52	0.693±0.049	54±30	449±169 (10)	771±131 (10)	973±44	38±56	0
7	0	2±2	—	2±2	599±238 (6)	Failed	Failed	15±17	0
7	2	40±43	0.661±0.161	32±34	579±137 (10)	836±112 (8)	1000±28 (2)	9±10	0
7	3	121±120	0.719±0.038	80±69	546±66 (10)	735±131 (10)	803±75 (3)	27±30	0
7	4	69±64	0.701±0.098	45±39	560±249 (10)	726±84 (7)	941±55 (2)	12±18	0
7	5	61±34	0.735±0.055	43±21	467±188 (10)	796±77 (10)	1026±4 (2)	11±15	0
8	0	1±2	—	1±1	556±225 (5)	1010±0 (1)	Failed	24±32	0
8	2	80±90	0.697±0.074	51±60	604±153 (10)	775±119 (8)	882±94 (3)	8±11	0
8	3	79±86	0.714±0.028	58±67	579±88 (10)	769±131 (9)	920±139 (3)	7±6	0
8	4	68±85	0.671±0.044	45±55	537±202 (10)	786±115 (6)	902±49 (3)	20±23	0
8	5	88±61	0.711±0.098	64±45	459±184 (10)	757±118 (9)	960±33 (4)	15±27	0
9	0	1±1	—	1±1	564±226 (5)	Failed	Failed	11±11	0
9	2	49±53	0.700±0.119	36±34	620±171 (10)	826±115 (8)	953±12 (2)	5±8	0
9	3	87±80	0.740±0.034	53±38	599±92 (10)	787±100 (10)	935±122 (3)	11±10	0
9	4	65±49	0.689±0.079	48±41	518±187 (10)	798±88 (10)	910±0 (1)	11±17	
9	5	98±82	0.695±0.097	60±51	459±180 (10)	774±80 (10)	907±93 (3)	21±26	0
10	0	2±5	—	1±3	471±160 (4)	955±0 (1)	N/A	14±15	0
10	2	23±20	0.690±0.100	19±16	594±139 (9)	884±77 (6)	N/A	8±14	0
10	3	54±71	0.702±0.047	42±53	609±106 (9)	822±118 (7)	848±0 (1)	7±11	0
10	4	61±34	0.744±0.038	47±26	510±181 (10)	829±69 (10)	993±11 (2)	5±6	0
10	5	122±95	0.739±0.032	85±65	455±171 (10)	770±81 (10)	877±47 (4)	31±42	0

Table S14: Beam Enumeration batch size 32 with Structure and Minimum Size 15. Filter Limit is the number of times that no SMILES contained the pool substructure in 100,000 generation epochs. Patience N/A indicates just Augmented Memory and no Beam Enumeration.

Patience	Aug. Rounds	Yield	IntDiv1	Scaffolds	OB 1	OB 10	OB 100	Repeats	Filter Limit
N/A	0	0±0	—	0±0	798±101 (5)	Failed	Failed	1±1	N/A
N/A	2	43±25	0.825±0.029	42±24	608±151 (10)	844±90 (9)	Failed	0±0	N/A
N/A	3	52±34	0.81±0.059	51±32	522±141 (10)	789±100 (9)	1018±0 (2)	0±1	N/A
N/A	4	87±33	0.82±0.018	83±31	466±120 (10)	740±77 (10)	987±30 (4)	1±3	N/A
N/A	5	98±57	0.817±0.027	89±50	408±184 (10)	714±136 (10)	915±20 (4)	1±2	N/A
5	0	2±4	0.611±0.074	2±3	776±155 (4)	983±0 (1)	Failed	43±30	0
5	2	18±27	0.666±0.077	15±19	705±173 (8)	857±104 (4)	Failed	9±9	0
5	3	26±20	0.652±0.076	19±11	618±88 (10)	850±108 (7)	Failed	16±18	0
5	4	65±64	0.695±0.092	54±53	604±214 (10)	742±124 (6)	936±55 (3)	65±90	0
5	5	99±110	0.713±0.046	66±61	452±216 (10)	741±173 (9)	870±146 (4)	64±56	0
6	0	2±5	0.655±0.051	2±4	614±213 (4)	836±0 (1)	Failed	39±27	0
6	2	36±49	0.691±0.096	32±47	625±188 (9)	834±139 (7)	943±31 (2)	9±9	0
6	3	60±58	0.662±0.124	47±53	574±148 (10)	811±146 (10)	895±81 (2)	93±220	0
6	4	67±52	0.654±0.185	54±43	592±214 (10)	740±133 (8)	934±50 (3)	114±154	0
6	5	66±70	0.68±0.059	50±44	530±209 (10)	822±141 (9)	933±69 (3)	65±70	0
7	0	1±2	—	1±2	686±161 (6)	Failed	Failed	83±78	0
7	2	49±60	0.699±0.101	41±56	601±156 (10)	821±152 (8)	923±93 (2)	18±20	0
7	3	47±46	0.670±0.107	37±36	623±198 (9)	810±161 (8)	994±16 (3)	20±21	0
7	4	41±45	0.686±0.058	33±42	588±81 (9)	838±94 (9)	905±0 (1)	53±43	0
7	5	76±76	0.698±0.111	66±74	531±210 (10)	776±128 (8)	866±69 (2)	126±325	0
8	0	16±37	—	14±33	749±210 (8)	668±194 (2)	949±0 (1)	109±163	0
8	2	33±48	0.691±0.049	24±33	692±144 (9)	856±142 (6)	974±35 (2)	15±18	0
8	3	50±30	0.675±0.068	40±22	636±109 (10)	803±84 (8)	Failed	39±49	0
8	4	104±104	0.73±0.056	84±96	406±128 (10)	696±149 (9)	879±141 (4)	30±36	0
8	5	42±30	0.7±0.051	32±18	506±186 (10)	848±95 (10)	974±0 (1)	30±45	0
9	0	7±12	—	6±10	713±201 (7)	848±1 (2)	Failed	68±50	0
9	2	36±34	0.686±0.052	28±28	559±138 (10)	812±96 (7)	1015±0 (1)	29±28	0
9	3	81±89	0.668±0.102	52±52	598±186 (10)	732±159 (7)	826±49 (3)	23±19	0
9	4	158±103	0.723±0.041	104±63	432±104 (10)	639±115 (10)	868±106 (7)	60±78	0
9	5	91±66	0.707±0.036	57±35	453±194 (10)	763±131 (10)	928±65 (4)	40±29	0
10	0	2±3	—	2±3	768±107 (5)	1003±0 (1)	Failed	93±97	0
10	2	55±54	0.722±0.027	44±40	559±156 (10)	807±149 (10)	836±0 (1)	26±39	0
10	3	86±46	0.705±0.063	67±36	478±143 (10)	678±114 (9)	962±33 (4)	41±50	0
10	4	99±77	0.705±0.048	63±43	474±162 (10)	693±91 (9)	944±113 (4)	58±86	0
10	5	110±100	0.715±0.039	80±78	430±164 (10)	750±142 (10)	881±107 (4)	57±55	0

2.3.2 Hypothesis 2

The use of “Structure” substructure is too biased when operating in an already biased environment: increasing augmentation rounds and under a low oracle budget.

Proposed solution. Investigate “Scaffold” substructure which is less biased.

Observations. Across batch sizes = [64, 32] and all Patience = [5, 6, 7, 8, 9, 10], sample efficiency does not improve over just using Augmented Memory and IntDiv1 (diversity) also decreases (sometimes by > 0.100) (Tables S15 and S16). Similar to the “Structure” setting, adding Beam Enumeration with the "Scaffold" setting neither increases Yield nor diversity.

Table S15: Beam Enumeration batch size 64 with Scaffold and Minimum Size 15. Filter Limit is the number of times that no SMILES contained the pool substructure in 100,000 generation epochs. Patience N/A indicates just Augmented Memory and no Beam Enumeration.

Patience	Aug. Rounds	Yield	IntDiv1	Scaffolds	OB 1	OB 10	OB 100	Repeats	Filter Limit
N/A	0	0±0	—	0±0	584±251 (5)	Failed	Failed	1±1	N/A
N/A	2	15±9	0.775±0.073	15±9	644±173 (10)	941±58 (8)	Failed	0±0	N/A
N/A	3	33±42	0.788±0.043	32±40	613±96 (10)	927±128 (9)	993±0 (1)	0±0	N/A
N/A	4	32±16	0.813±0.024	31±16	527±198 (10)	880±90 (10)	Failed	0±0	N/A
N/A	5	40±14	0.812±0.023	39±13	459±177 (10)	862±68 (10)	Failed	0±0	N/A
5	0	5±17	0.726±0.0	5±15	653±275 (3)	819±0 (1)	Failed	48±31	0
5	2	14±22	0.616±0.182	13±21	635±226 (7)	850±131 (3)	Failed	36±29	0
5	3	21±26	0.675±0.116	18±22	647±198 (8)	852±88 (5)	Failed	19±26	0
5	4	20±30	0.6±0.122	18±26	592±262 (9)	869±108 (4)	1038±0 (1)	28±19	0
5	5	33±27	0.692±0.082	29±25	506±208 (10)	875±101 (8)	Failed	33±37	0
6	0	0±1	0.399±0.0	0±0	433±98 (4)	Failed	Failed	98±99	0
6	2	9±16	0.656±0.072	7±13	713±237 (8)	864±82 (2)	Failed	30±25	0
6	3	16±19	0.645±0.072	14±18	662±152 (8)	905±103 (5)	Failed	27±30	0
6	4	15±23	0.644±0.069	14±22	466±185 (8)	884±137 (4)	Failed	23±16	0
6	5	24±28	0.599±0.139	21±22	583±293 (10)	849±83 (5)	1014±0 (1)	35±38	0
7	0	0±1	—	0±1	459±139 (4)	Failed	Failed	82±47	0
7	2	10±10	0.64±0.072	9±10	666±180 (9)	911±76 (3)	Failed	37±59	0
7	3	27±31	0.659±0.119	23±23	648±153 (9)	880±122 (7)	1041±0 (1)	11±8	0
7	4	20±19	0.634±0.125	19±18	575±249 (10)	853±72 (5)	Failed	46±59	0
7	5	14±13	0.676±0.096	12±10	519±267 (10)	932±75 (6)	Failed	24±32	0
8	0	0±0	—	0±0	383±53 (3)	Failed	Failed	36±23	0
8	2	10±13	0.665±0.131	10±12	654±201 (8)	910±85 (4)	Failed	15±19	0
8	3	30±48	0.693±0.031	29±46	624±164 (9)	863±129 (6)	901±0 (1)	24±21	0
8	4	29±43	0.667±0.095	23±30	571±268 (9)	745±98 (4)	981±0 (1)	20±26	0
8	5	40±47	0.665±0.093	35±45	450±168 (10)	879±95 (9)	920±0 (1)	43±74	0
9	0	0±0	—	0±0	500±207 (4)	Failed	Failed	31±29	0
9	2	20±36	0.683±0.055	19±36	683±226 (9)	825±84 (3)	1005±0 (1)	8±9	0
9	3	41±34	0.675±0.08	34±28	654±155 (10)	849±134 (8)	Failed	25±22	0
9	4	16±14	0.647±0.093	13±11	573±240 (10)	917±39 (5)	Failed	10±11	0
9	5	39±24	0.707±0.083	34±22	456±172 (10)	829±67 (9)	Failed	8±9	0
10	0	3±8	—	3±7	519±171 (5)	851±0 (1)	Failed	16±26	0
10	2	16±19	0.674±0.07	13±15	599±144 (9)	905±95 (5)	Failed	17±20	0
10	3	32±38	0.703±0.074	26±27	621±107 (10)	861±129 (8)	961±0 (1)	5±7	0
10	4	18±15	0.682±0.087	16±15	529±202 (10)	876±81 (7)	Failed	5±8	0
10	5	37±31	0.711±0.057	30±20	456±172 (10)	829±68 (8)	996±0 (1)	23±42	0

Table S16: Beam Enumeration batch size 32 with Scaffold and Minimum Size 15. Filter Limit is the number of times that no SMILES contained the pool substructure in 100,000 generation epochs. Patience N/A indicates just Augmented Memory and no Beam Enumeration.

Patience	Aug. Rounds	Yield	IntDiv1	Scaffolds	OB 1	OB 10	OB 100	Repeats	Filter Limit
N/A	0	0±0	—	0±0	798±101 (5)	Failed	Failed	1±1	N/A
N/A	2	43±25	0.825±0.029	42±24	608±151 (10)	844±90 (9)	Failed	0±0	N/A
N/A	3	52±34	0.81±0.059	51±32	522±141 (10)	789±100 (9)	1018±0 (2)	0±1	N/A
N/A	4	87±33	0.82±0.018	83±31	466±120 (10)	740±77 (10)	987±30 (4)	1±3	N/A
N/A	5	98±57	0.817±0.027	89±50	408±184 (10)	714±136 (10)	915±20 (4)	1±2	N/A
5	0	0±0	—	0±0	852±141 (2)	Failed	Failed	119±78	0
5	2	25±38	0.65±0.109	23±35	698±191 (8)	779±127 (4)	959±0 (1)	57±67	0
5	3	33±59	0.629±0.073	26±44	636±148 (8)	867±133 (6)	871±0 (1)	88±123	1
5	4	57±68	0.666±0.032	44±51	648±163 (9)	834±128 (7)	952±70 (3)	118±104	0
5	5	50±69	0.649±0.038	33±39	498±268 (9)	855±170 (8)	890±3 (2)	89±46	0
6	0	2±6	—	2±6	788±161 (3)	840±0 (1)	Failed	174±112	0
6	2	25±59	0.618±0.148	16±36	672±240 (7)	694±238 (3)	706±0 (1)	53±55	1
6	3	35±47	0.667±0.119	27±35	702±189 (8)	789±93 (5)	974±0 (2)	52±43	0
6	4	46±66	0.653±0.068	39±56	656±127 (9)	831±144 (6)	945±67 (2)	135±206	0
6	5	57±76	0.584±0.157	45±59	571±274 (8)	668±83 (4)	907±7 (3)	101±113	0
7	0	14±27	0.551±0.116	10±17	663±109 (5)	814±130 (3)	Failed	106±58	0
7	2	19±41	0.657±0.121	12±24	660±127 (6)	894±136 (5)	929±0 (1)	34±23	0
7	3	38±51	0.636±0.115	28±30	650±161 (10)	812±131 (6)	863±0 (1)	45±33	0
7	4	36±36	0.652±0.109	26±21	700±151 (10)	811±76 (7)	981±0 (1)	67±49	0
7	5	46±45	0.608±0.108	39±40	485±204 (9)	810±50 (6)	991±5 (2)	237±244	0
8	0	0±0	—	0±0	794±302 (4)	Failed	Failed	149±100	0
8	2	34±45	0.625±0.105	30±39	696±175 (9)	777±105 (5)	901±0 (1)	57±46	0
8	3	53±77	0.543±0.174	42±61	652±213 (9)	715±141 (5)	836±6 (2)	57±87	1
8	4	30±53	0.631±0.092	24±39	684±235 (9)	781±165 (3)	957±51 (2)	54±43	0
8	5	90±101	0.632±0.124	70±74	556±248 (9)	706±127 (6)	879±78 (4)	179±158	0
9	0	0±0	—	0±0	733±157 (3)	Failed	Failed	175±142	0
9	2	20±37	0.61±0.124	15±25	643±237 (8)	849±152 (4)	967±0 (1)	61±69	0
9	3	28±25	0.639±0.09	23±20	661±121 (10)	819±78 (6)	Failed	53±60	0
9	4	67±63	0.66±0.105	55±56	605±203 (9)	783±126 (8)	906±58 (2)	92±65	0
9	5	55±73	0.618±0.13	36±41	513±225 (9)	779±149 (6)	877±74 (2)	150±206	0
10	0	2±5	—	1±3	835±154 (4)	890±0 (1)	Failed	93±68	0
10	2	5±4	—	4±3	680±196 (8)	960±0 (1)	Failed	58±52	0
10	3	32±48	0.636±0.143	31±47	572±171 (10)	880±130 (7)	900±0 (1)	30±36	0
10	4	44±32	0.693±0.059	34±26	503±195 (10)	811±126 (9)	965±0 (1)	107±125	0
10	5	51±55	0.581±0.206	36±37	584±317 (9)	712±88 (5)	949±34 (2)	156±239	1

2.3.3 Hypothesis 3

In the original Beam Enumeration [2] work, enforcing a Structure Minimum Size for extracted substructures improves sample efficiency across all hyperparameter combinations (and is statistically significant). The results so far suggest that this observation does not hold when optimizing under a particularly low oracle budget (1000 calls). Thus far, experiments were aimed at mitigating the Beam Enumeration bias either by tuning the Patience parameter or by changing the Substructure Type. Another method to mitigate bias is by not enforcing a Structure Minimum Size. In this scenario, Scaffold substructure should be used as Structure substructure tends to extract small functional groups (as observed in the original work).

Proposed solution. Investigate "Scaffold" substructure without enforcing Structure Minimum Size.

Observations. Across batch sizes = [64, 32] and all Patience = [5, 6, 7, 8, 9, 10], sample efficiency *sometimes* improves (Tables S17 and S18). Variance is also manageable but the performance improvements, when observed, is much less than with lower batch size and higher augmentation rounds (for instance Mamba batch size 16 and augmentation rounds 10).

Table S17: Beam Enumeration batch size 64 with Scaffold and no Minimum Size enforced. Filter Limit is the number of times that no SMILES contained the pool substructure in 100,000 generation epochs. Patience N/A indicates just Augmented Memory and no Beam Enumeration.

Patience	Aug. Rounds	Yield	IntDiv1	Scaffolds	OB 1	OB 10	OB 100	Repeats	Filter Limit
N/A	0	0±0	—	0±0	584±251 (5)	Failed	Failed	1±1	0
N/A	2	15±9	0.775±0.073	15±9	644±173 (10)	941±58 (8)	Failed	0±0	0
N/A	3	33±42	0.788±0.043	32±40	613±96 (10)	927±128 (9)	993±0 (1)	0±0	0
N/A	4	32±16	0.813±0.024	31±16	527±198 (10)	880±90 (10)	Failed	0±0	0
N/A	5	40±14	0.812±0.023	39±13	459±177 (10)	862±68 (10)	Failed	0±0	0
5	0	0±0	—	0±0	307±0 (1)	Failed	Failed	0±0	0
5	2	15±12	0.744±0.068	14±11	678±227 (10)	930±70 (5)	Failed	0±0	0
5	3	38±14	0.791±0.026	37±14	552±70 (10)	824±44 (9)	Failed	0±0	0
5	4	43±45	0.791±0.021	42±43	516±230 (10)	839±132 (9)	918±0 (1)	0±0	0
5	5	55±33	0.77±0.073	50±30	467±197 (10)	811±81 (9)	961±0 (1)	0±1	0
6	0	0±0	—	0±0	594±268 (5)	Failed	Failed	0±0	0
6	2	28±23	0.752±0.053	26±21	671±190 (10)	880±72 (6)	Failed	0±0	0
6	3	44±28	0.782±0.032	42±24	584±120 (10)	832±64 (9)	1006±0 (1)	0±0	0
6	4	41±37	0.778±0.028	39±36	571±241 (10)	874±118 (9)	959±0 (1)	0±0	0
6	5	54±21	0.794±0.025	49±17	453±169 (10)	827±72 (10)	Failed	0±0	0
7	0	0±0	—	0±0	567±234 (5)	Failed	Failed	0±1	0
7	2	27±13	0.778±0.072	27±13	603±148 (10)	880±80 (9)	Failed	0±0	0
7	3	47±33	0.797±0.027	44±30	586±73 (10)	859±113 (10)	1035±1 (2)	0±0	0
7	4	48±23	0.799±0.017	45±20	498±176 (10)	828±87 (10)	Failed	0±0	0
7	5	51±23	0.793±0.023	48±21	463±190 (10)	854±72 (10)	Failed	0±0	0
8	0	0±0	—	0±0	383±53 (3)	Failed	Failed	0±0	0
8	2	20±12	0.755±0.072	20±12	637±153 (10)	929±62 (8)	Failed	0±0	0
8	3	39±32	0.793±0.021	38±31	593±85 (10)	882±111 (10)	962±0 (1)	0±0	0
8	4	47±30	0.793±0.024	45±29	544±208 (10)	873±75 (10)	1013±0 (1)	0±0	0
8	5	69±28	0.803±0.019	64±22	446±162 (10)	789±73 (10)	991±0 (1)	0±0	0
9	0	0±0	—	0±0	656±281 (6)	Failed	Failed	0±0	0
9	2	16±10	0.761±0.041	16±10	640±166 (10)	946±48 (6)	Failed	0±0	0
9	3	52±60	0.798±0.021	49±55	619±106 (10)	847±107 (10)	847±0 (1)	0±0	0
9	4	50±25	0.802±0.01	48±22	505±177 (10)	846±79 (10)	1004±0 (1)	0±0	0
9	5	54±26	0.792±0.024	50±24	450±165 (10)	809±55 (9)	Failed	0±0	0
10	0	0±0	—	0±0	636±260 (6)	Failed	Failed	0±0	0
10	2	21±17	0.739±0.091	21±17	643±178 (10)	920±78 (8)	Failed	0±0	0
10	3	46±48	0.791±0.024	43±43	613±99 (10)	853±115 (9)	899±0 (1)	0±0	0
10	4	44±35	0.783±0.041	42±33	541±222 (10)	858±89 (9)	990±0 (1)	0±0	0
10	5	48±18	0.792±0.024	45±15	456±173 (10)	853±50 (10)	Failed	0±0	0

Conclusions. Based on the grid-search results, Beam Enumeration can *sometimes* improve sample efficiency when using "Scaffold" structure and without enforcing Structure Minimum Size. However, the improvements are minor, such that it would be better to use small batch sizes with high augmentation rounds. Thus, we do not further experiment with Beam Enumeration in this work.

Table S18: Beam Enumeration batch size 32 with Scaffold and no Minimum Size enforced. Filter Limit is the number of times that no SMILES contained the pool substructure in 100,000 generation epochs. Patience N/A indicates just Augmented Memory and no Beam Enumeration.

Patience	Aug. Rounds	Yield	IntDiv1	Scaffolds	OB 1	OB 10	OB 100	Repeats	Filter Limit
N/A	0	0±0	—	0±0	798±101 (5)	Failed	Failed	1±1	0
N/A	2	43±25	0.825±0.029	42±24	608±151 (10)	844±90 (9)	Failed	0±0	0
N/A	3	52±34	0.81±0.059	51±32	522±141 (10)	789±100 (9)	1018±0 (2)	0±1	0
N/A	4	87±33	0.82±0.018	83±31	466±120 (10)	740±77 (10)	987±30 (4)	1±3	0
N/A	5	98±57	0.817±0.027	89±50	408±184 (10)	714±136 (10)	915±20 (4)	1±2	0
5	0	0±1	—	0±1	783±134 (3)	Failed	Failed	0±1	0
5	2	38±28	0.796±0.03	35±25	504±111 (9)	828±115 (9)	Failed	1±1	0
5	3	63±44	0.762±0.073	57±38	593±170 (10)	763±82 (8)	988±29 (3)	1±2	0
5	4	87±57	0.779±0.038	72±43	540±145 (10)	764±139 (10)	958±48 (5)	2±4	0
5	5	106±61	0.784±0.031	84±41	467±187 (10)	718±109 (10)	960±41 (6)	1±2	0
6	0	1±3	—	1±3	837±135 (3)	998±0 (1)	Failed	2±2	0
6	2	40±33	0.761±0.078	36±29	609±149 (9)	811±64 (7)	1014±0 (1)	1±2	0
6	3	49±23	0.796±0.03	46±21	585±104 (10)	839±101 (10)	Failed	1±2	0
6	4	57±41	0.783±0.031	53±37	557±187 (10)	771±82 (8)	987±10 (3)	1±2	0
6	5	106±85	0.776±0.05	85±55	508±241 (10)	718±151 (9)	927±94 (5)	3±6	0
7	0	0±0	—	0±0	741±222 (5)	Failed	Failed	1±1	0
7	2	43±27	0.79±0.037	41±26	631±182 (10)	799±77 (8)	Failed	0±0	0
7	3	84±67	0.79±0.021	73±56	578±188 (10)	781±117 (9)	937±42 (4)	0±1	0
7	4	74±43	0.785±0.041	69±37	574±149 (10)	789±111 (10)	948±39 (2)	1±3	0
7	5	121±52	0.786±0.033	105±39	422±155 (10)	673±90 (10)	898±52 (5)	4±9	0
8	0	3±5	—	3±5	683±213 (5)	882±0 (1)	Failed	2±3	0
8	2	44±39	0.713±0.166	40±30	629±177 (10)	778±97 (7)	995±0 (1)	1±4	0
8	3	69±43	0.794±0.039	65±40	530±183 (10)	778±104 (9)	975±8 (3)	0±2	0
8	4	75±39	0.795±0.033	66±30	547±142 (10)	770±118 (10)	981±29 (3)	1±1	0
8	5	103±55	0.761±0.091	90±49	488±221 (10)	693±142 (9)	961±39 (7)	4±5	0
9	0	2±4	—	2±4	805±127 (4)	915±0 (1)	Failed	1±1	0
9	2	41±23	0.79±0.022	40±22	572±132 (10)	839±95 (10)	Failed	0±0	0
9	3	59±34	0.81±0.021	54±31	520±110 (9)	778±68 (9)	993±0 (1)	0±1	0
9	4	101±60	0.799±0.025	89±45	515±142 (10)	725±104 (10)	944±91 (4)	1±1	0
9	5	128±61	0.792±0.022	102±41	425±179 (10)	684±93 (10)	919±51 (6)	2±2	0
10	0	0±1	—	0±1	822±160 (4)	Failed	Failed	1±1	0
10	2	53±45	0.795±0.025	49±44	515±129 (9)	793±106 (9)	973±30 (2)	2±5	0
10	3	86±63	0.759±0.119	73±46	553±179 (10)	720±62 (8)	956±69 (4)	0±1	0
10	4	89±35	0.794±0.034	77±26	464±132 (10)	743±51 (10)	984±27 (4)	3±5	0
10	5	123±58	0.795±0.031	105±44	434±177 (10)	704±102 (10)	949±59 (8)	2±2	0

2.4 Hallucinated Memory: Is it beneficial to allocate a portion of the oracle budget to hallucination?

In this section, we investigate coupling GraphGA [13] to Saturn. GraphGA in itself a sample-efficient generative algorithm [40] and was recently used in the GEAM model proposed by Lee et al. [43] which achieves impressive MPO performance. Previously work [42] found that coupling a GA in RL can encourage diverse sampling. In the previous sections, we have identified Mamba with batch size 16 and 10 augmentation rounds as the best hyperparameters so far. The improved sample efficiency comes at a trade-off in diversity. The objective in the experiments to follow is to investigate whether allocating a portion of the oracle budget to GraphGA generation (which we call "hallucinating") is beneficial in recovering diversity while maintaining sample efficiency.

Before presenting the grid-search results, we describe the GraphGA integration further. GraphGA is only activated when the replay buffer is full (100 SMILES). Once full, at every epoch thereafter, the replay buffer itself is treated as the parent population to generate new SMILES. These new SMILES are then concatenated with the sampled batch (16 SMILES) and used to update the Agent. Importantly, these hallucinated SMILES are also deposited into the replay buffer (if they possess higher reward). Finally, 100 SMILES are hallucinated and either 5 or 10 are selected. The selection criteria are **Random** or **Tanimoto Distance**. Random selects at random while Tanimoto Distance selects via maximum fingerprint *dissimilarity* to the replay buffer. Our rationale is that dissimilar new SMILES will help encourage diversity since Augmented Memory heavily biases towards the replay buffer SMILES.

The grid-search investigated the following hyperparameter settings:

1. Fix Mamba with batch size 16
2. Augmentation Rounds = [5,20]
3. GA with Random and Tanimoto Distance selection criterion
4. Select 5 or 10 hallucinations at every epoch

The reason we increased the augmentation rounds back to 20 in our grid-search is because if indeed the GA recovers diversity, then the "augmentation tolerability" of Saturn would probably be

increased. Higher augmentation rounds lead to more repeated SMILES precisely due to overfitting. If new high reward SMILES *refresh* the replay buffer, Saturn may be able to improve with even higher augmentation rounds (than 10) to potentially further improve sample efficiency. The results of the grid-search are presented in Tables S19 and S20.

Observations. The results show that coupling a GA to the replay buffer does not improve sample efficiency. However, we make several interesting observations. Firstly, the number of repeated SMILES *notably* drops and IntDiv1 [51] recovers. Secondly, hallucinating SMILES does indeed lead to some replacement of the replay buffer, and hence, these SMILES are necessarily high reward (see note below), but rarely are they better than the best in the buffer. Finally, we note that hallucinated SMILES are generated off-policy and Agent updates may be more meaningful with importance sampling [46], which we did not explore this this work.

Note. Since the pre-print version of this work, we identified a bug in the tracking code for the number of times a GA-generated SMILES is deposited in the replay buffer. For this reason, we have removed these values from Tables S19 and S20. We have since implemented a fix and re-ran the following configurations for 100 replicates each: GA with Random selection (5 or 10) with augmentation rounds (10 or 20) for a total of 4 unique experiments (400 replicates). We made the decision to run these 4 unique experiments instead of the same hyperparameter sweep for the following reasons: (1) GA random selection generally outperforms the Tanimoto distance selection criterion as shown in Tables S19 and S20 so we focus on better understanding the random selection criterion. (2) Previously, Mamba with batch size 16 (fixed here) and 10 augmentation rounds was the optimal hyperparameter combination so we wanted to focus on 10 augmentation rounds. (3) One of our hypotheses is that the GA can recover diversity and thus increase the "augmentation tolerability", where the augmentation rounds can increase without being prone to mode collapse. Therefore, we run 20 augmentation rounds since this is the upper-bound we have been testing. As expected, 20 augmentation rounds with the GA activated outperforms 10 augmentation rounds (Tables S19 and S20).

The **Buffer Replace** metric: The number of times a hallucinated SMILES replaced another SMILES in the buffer will help us better understand whether this performance difference can at least be partly attributed to depositing more GA-generated SMILES in the buffer. Since we focus on 4 unique experiments, we run 100 replicates each. The results are as follows (mean and standard

deviation across the 100 replicates):

- GA-Random-5 with 10 Augmentation Rounds: 90±11
- GA-Random-5 with 20 Augmentation Rounds: 89±17
- GA-Random-10 with 10 Augmentation Rounds: 134±17
- GA-Random-10 with 20 Augmentation Rounds: 132±18

The results confirm that GA-generated SMILES do refresh the buffer. Selecting more GA-generated SMILES leads to more molecules refreshing the buffer. Interestingly and empirically, the number of augmentation rounds has minimal effect on this Buffer Replace metric. The results support our hypothesis that activating the GA increases the "augmentation tolerability": Since GA-generated SMILES refresh the buffer, it causes the model to be less prone to mode collapse. Cross-referencing Tables S19 and S20, the IntDiv1 is maintained even at higher augmentation rounds and suggests that the increased diversity of the GA-generated molecules relaxes the exploitation behavior of Augmented Memory.

2.5 Saturn: Final Hyperparameters

The most sample-efficient hyperparameter settings, on average, are: **Mamba with batch size 16 and 10 augmentation rounds**. The results in the immediate previous section shows that the GA can better maintain sample diversity, which can be a useful component that can easily be activated on and off depending on the oracle setting. However, by maintaining diversity, the model is less sample-efficient (in our test setting). This observation further illustrates the efficiency-diversity trade-off.

Table S19: Mamba batch size 16 with GraphGA [13] applied on the replay buffer. The hallucinated SMILES were selected at *Random*. **Hall. Yield** is the yield from GraphGA. **Buffer Best** is the number of times at least one of the SMILES in a given hallucinated set was better than the top-1 in the buffer.

GA Random	Aug. Rounds	Hall. Yield	Total Yield	Buffer Best	IntDiv1	Scaffolds	OB 1	OB 10	OB 100	Sampled Repeats	Hall. Repeats
5	5	9±7	54±43	2±1	0.756±0.043	45±33	538±212 (10)	812±114 (9)	989±27 (3)	58±39	5±3
5	6	21±10	88±56	3±1	0.773±0.046	68±41	457±122 (10)	729±103 (10)	936±83 (3)	57±29	6±3
5	7	11±9	57±42	3±2	0.73±0.063	49±37	619±125 (10)	795±116 (9)	988±13 (3)	122±50	6±3
5	8	14±11	63±42	3±2	0.758±0.044	49±25	574±166 (10)	793±96 (10)	916±0 (1)	177±80	6±3
5	9	20±15	106±75	2±1	0.767±0.03	86±55	531±128 (10)	733±121 (10)	833±57 (3)	207±101	9±5
5	10	21±11	113±61	2±1	0.742±0.04	83±38	496±158 (10)	690±118 (10)	910±59 (5)	257±143	7±3
5	11	15±11	102±69	3±2	0.739±0.031	69±43	552±141 (10)	730±116 (10)	887±62 (4)	308±116	7±3
5	12	29±17	139±83	3±1	0.781±0.025	101±55	488±104 (10)	666±92 (10)	856±76 (5)	339±153	9±4
5	13	25±14	144±97	3±1	0.727±0.048	94±50	463±209 (10)	658±155 (10)	843±99 (6)	511±226	10±4
5	14	36±22	176±82	3±2	0.742±0.038	133±56	475±121 (10)	640±110 (10)	863±92 (8)	691±333	13±7
5	15	42±17	208±65	4±2	0.746±0.06	167±58	401±115 (10)	595±89 (10)	844±91 (10)	693±319	13±8
5	16	34±9	187±77	5±2	0.744±0.055	150±59	421±119 (10)	624±106 (10)	829±83 (8)	789±465	10±5
5	17	33±25	181±95	3±1	0.750±0.042	127±64	469±142 (10)	664±132 (10)	838±86 (8)	830±417	10±6
5	18	35±18	164±57	4±2	0.727±0.038	133±54	459±105 (10)	637±76 (10)	872±66 (8)	881±389	16±16
5	19	30±16	190±76	3±1	0.744±0.046	145±51	467±123 (10)	630±113 (10)	822±59 (8)	1072±465	12±9
5	20	44±18	247±83	3±1	0.748±0.034	185±60	380±144 (10)	566±115 (10)	761±59 (9)	1310±512	14±6
10	5	12±10	44±44	3±1	0.77±0.066	35±29	478±206 (10)	802±133 (9)	888±0 (1)	24±14	8±5
10	6	16±13	44±34	4±2	0.784±0.023	37±29	534±139 (10)	812±87 (9)	936±0 (1)	38±19	8±4
10	7	14±9	43±27	4±2	0.739±0.109	37±23	594±117 (10)	800±54 (9)	Failed	61±34	9±4
10	8	20±16	55±41	4±2	0.771±0.026	46±30	520±114 (10)	805±129 (10)	924±0 (1)	71±30	9±4
10	9	22±18	70±51	4±2	0.753±0.04	57±42	520±174 (10)	788±149 (10)	952±44 (3)	113±58	11±7
10	10	17±16	65±63	4±2	0.714±0.104	48±37	539±183 (10)	758±141 (9)	773±0 (1)	138±69	11±6
10	11	18±11	57±47	5±1	0.761±0.031	42±29	605±139 (10)	789±104 (9)	931±38 (2)	192±90	10±7
10	12	37±37	88±79	4±1	0.734±0.092	70±59	591±142 (10)	716±119 (9)	882±110 (3)	222±106	17±14
10	13	29±25	84±84	3±1	0.727±0.078	61±51	502±195 (10)	737±169 (9)	842±52 (3)	260±134	13±7
10	14	29±16	97±64	5±2	0.756±0.046	72±44	456±217 (10)	733±164 (10)	908±9 (5)	271±116	9±6
10	15	37±24	102±64	4±1	0.759±0.03	85±48	480±184 (10)	688±162 (10)	913±77 (5)	336±182	19±10
10	16	40±22	110±60	5±3	0.754±0.028	91±50	432±200 (10)	691±149 (10)	913±55 (6)	361±185	15±10
10	17	34±22	103±62	5±2	0.75±0.048	80±47	529±154 (10)	704±117 (9)	916±45 (6)	467±214	15±8
10	18	25±15	91±52	5±1	0.745±0.03	64±31	562±102 (10)	750±88 (10)	927±42 (4)	572±322	17±10
10	19	25±14	88±46	6±2	0.750±0.036	71±39	563±127 (10)	751±114 (10)	948±33 (5)	603±236	16±9
10	20	38±24	136±80	6±1	0.748±0.059	95±48	444±150 (10)	626±117 (9)	867±90 (6)	781±360	13±5

Table S20: Mamba batch size 16 with GraphGA [13] applied on the replay buffer. The hallucinated SMILES were selected by highest *Tanimoto Distance (TanDist)*. **Hall. Yield** is the yield from GraphGA. **Buffer Best** is the number of times at least one of the SMILES in a given hallucinated set was better than the top-1 in the buffer.

GA TanDist	Aug. Rounds	Hall. Yield	Total Yield	Buffer Best	IntDiv1	Scaffolds	OB 1	OB 10	OB 100	Sampled Repeats	Hall. Repeats
5	5	12±11	68±60	2±1	0.770±0.050	57±46	532±244 (10)	752±125 (8)	913±51 (3)	50±35	17±7
5	6	8±8	61±73	1±1	0.763±0.041	51±57	602±171 (10)	834±151 (10)	890±110 (2)	62±36	17±11
5	7	15±8	68±460	4±2	0.776±0.035	60±38	610±62 (10)	797±86 (10)	855±0 (1)	122±59	17±8
5	8	11±8	89±61	2±1	0.765±0.031	72±45	473±120 (10)	753±116 (10)	888±42 (3)	156±84	14±8
5	9	22±17	123±86	2±1	0.757±0.049	97±66	471±187 (10)	712±164 (10)	872±96 5)	309±150	16±7
5	10	18±15	97±79	2±1	0.758±0.045	78±57	544±183 (10)	748±158 (10)	901±107 (4)	317±133	16±9
5	11	18±14	92±60	2±2	0.785±0.031	78±49	560±130 (10)	749±97 (10)	846±42 (2)	314±126	20±9
5	12	26±17	146±101	2±1	0.772±0.043	109±70	491±165 (10)	684±184 (10)	838±124 (6)	418±220	22±15
5	13	21±15	114±77	2±1	0.74±0.053	97±62	494±200 (10)	706±134 (9)	912±71 (6)	494±218	19±13
5	14	28±24	158±95	2±1	0.756±0.042	131±82	505±152 (10)	681±152 (10)	846±85 (7)	682±355	27±20
5	15	39±20	189±98	3±1	0.752±0.074	151±76	415±159 (10)	600±176 (10)	818±103 (8)	698±382	28±14
5	16	45±30	189±110	2±2	0.788±0.042	152±91	456±171 (10)	630±168 (10)	784±98 (7)	771±329	33±16
5	17	29±22	166±89	3±1	0.760±0.053	124±58	506±145 (10)	652±130 (10)	874±102 (8)	733±343	26±15
5	18	17±12	114±75	3±2	0.686±0.104	87±50	549±154 (10)	668±86 (8)	913±65 (6)	911±412	30±20
5	19	16±14	117±86	2±2	0.708±0.101	94±70	559±169 (10)	706±153 (9)	862±117 (5)	1287±520	24±23
5	20	32±16	183±72	3±2	0.752±0.072	151±60	417±161 (10)	628±111 (10)	878±102 (10)	1241±508	22±13
10	5	13±13	39±39	3±2	0.768±0.065	35±34	551±214 (9)	765±155 (7)	942±0 (1)	34±15	19±8
10	6	11±10	43±34	2±1	0.76±0.064	41±32	556±156 (10)	777±99 (7)	Failed	34±20	16±8
10	7	13±8	41±28	3±2	0.767±0.066	38±27	550±140 (10)	835±106 (9)	997±0 (1)	62±43	19±9
10	8	12±9	41±26	2±2	0.751±0.093	36±22	575±156 (10)	786±123 (9)	Failed	75±41	21±9
10	9	18±12	56±35	3±2	0.764±0.072	48±30	527±156 (10)	732±79 (8)	991±0 (1)	117±78	19±9
10	10	10±12	42±46	3±2	0.775±0.055	32±31	660±225 (10)	797±127 (7)	870±0 (1)	158±80	15±7
10	11	10±8	39±39	3±1	0.713±0.109	32±30	626±173 (10)	828±124 (7)	964±0 (1)	181±93	30±23
10	12	16±19	63±64	3±1	0.733±0.123	53±56	534±207 (10)	731±113 (8)	897±107 (2)	236±106	29±23
10	13	20±19	67±63	3±2	0.732±0.117	50±41	542±228 (9)	746±139 (8)	902±38 (3)	300±150	30±19
10	14	15±13	61±50	2±1	0.714±0.114	49±41	589±175 (10)	770±102 (8)	924±22 (2)	365±210	26±15
10	15	28±25	80±71	5±1	0.762±0.033	68±58	599±160 (10)	741±129 (8)	925±100 (4)	366±228	32±19
10	16	30±28	89±77	5±2	0.765±0.07	74±63	563±186 (10)	719±167 (9)	832±34 (3)	376±188	35±24
10	17	30±25	101±80	3±1	0.787±0.028	77±58	532±182 (9)	719±173 (9)	880±45 (5)	503±237	42±25
10	18	16±13	54±39	3±2	0.721±0.071	43±31	543±152 (10)	811±112 (9)	926±0 (1)	609±309	48±59
10	19	21±12	83±54	3±2	0.761±0.034	64±41	495±135 (9)	738±121 (9)	920±40 (4)	620±259	30±17
10	20	16±17	54±44	2±1	0.761±0.044	46±34	524±206 (9)	796±86 (8)	925±0 (1)	747±416	32±17

3 Mechanism of Augmented Memory and Mamba

In this subsection, we show additional results supporting our statement on Augmented Memory's [1] mechanism: Augmented Memory squeezes the likelihood of generating the Buffer *molecules* such that it becomes probable to generate *some* SMILES representation of them. In the main text, the experiment to show likelihood squeezing was as follows: starting from the pre-trained Mamba model, generate molecules until the Buffer is full and then save the Agent state before and after Augmented Memory. Every augmented Buffer SMILES was also saved. This experiment isolates the effect of Augmented Memory starting from the *initial state* of the pre-trained model.

The main text results showed that Mamba (batch size 16, augmentation rounds 10) exhibits "hop-and-locally-exploit" behavior but what about RNN (batch size 16, augmentation rounds 10)? We show that the RNN model also begins to exhibit this behavior but to a lesser extent (Figure S1), in agreement with the enhanced likelihood convergence observed for Mamba (Supplementary Information 1.6).

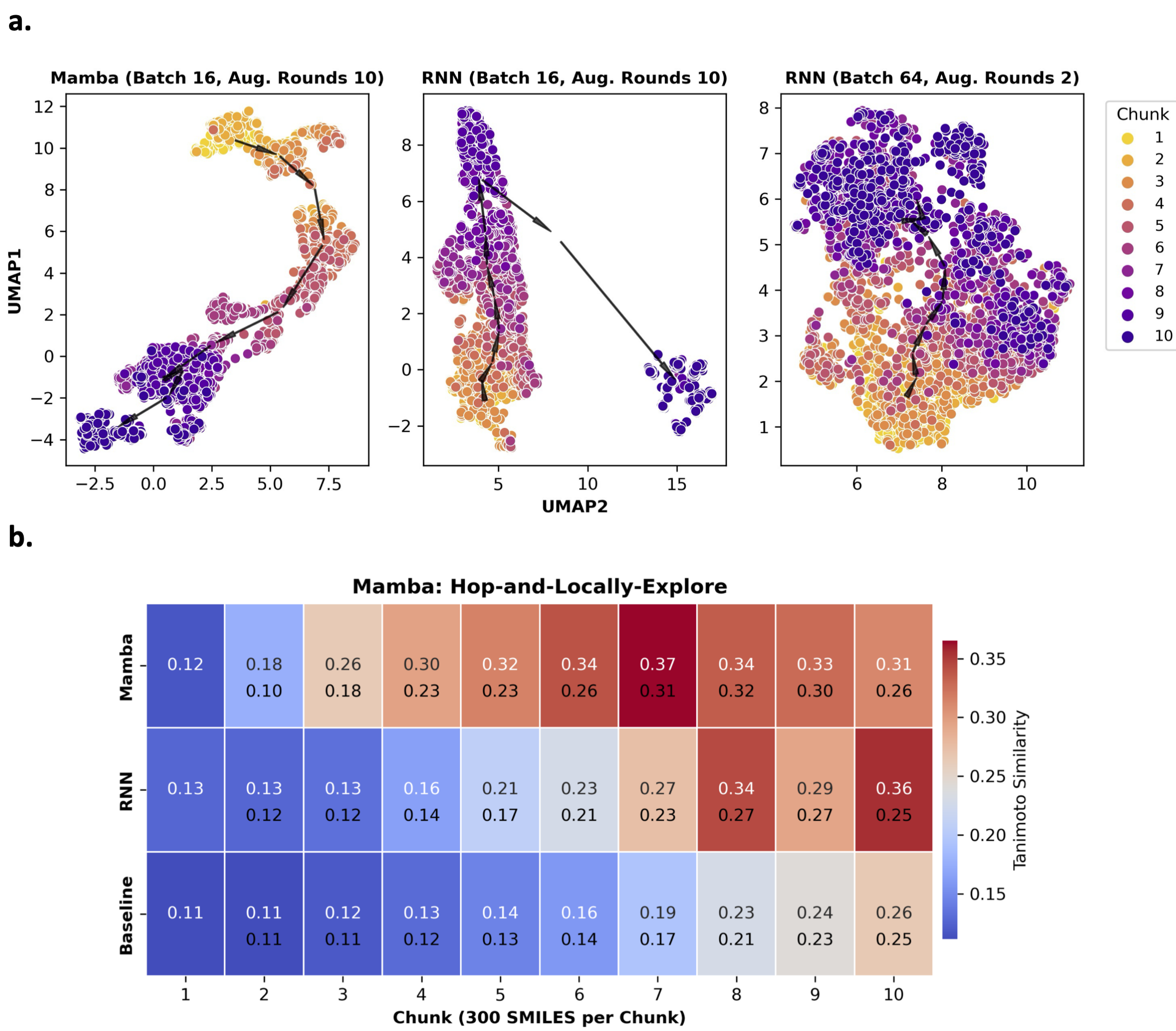

Figure S1: Mamba and RNN (both batch size 16, augmentation rounds 10) and baseline Augmented Memory (batch size 64, augmentation rounds 2). **a.** 3,000 oracle budget test experiment chunked into 300 SMILES. UMAP embedding of the Agent chemical space traversal (arrows are the centroid of each chunk). **b.** Mamba exhibits a "hop-and-locally-exploit" behavior where the intra-chunk Tanimoto similarity (top values) are higher than RNN. The bottom value is the inter-chunk similarity.

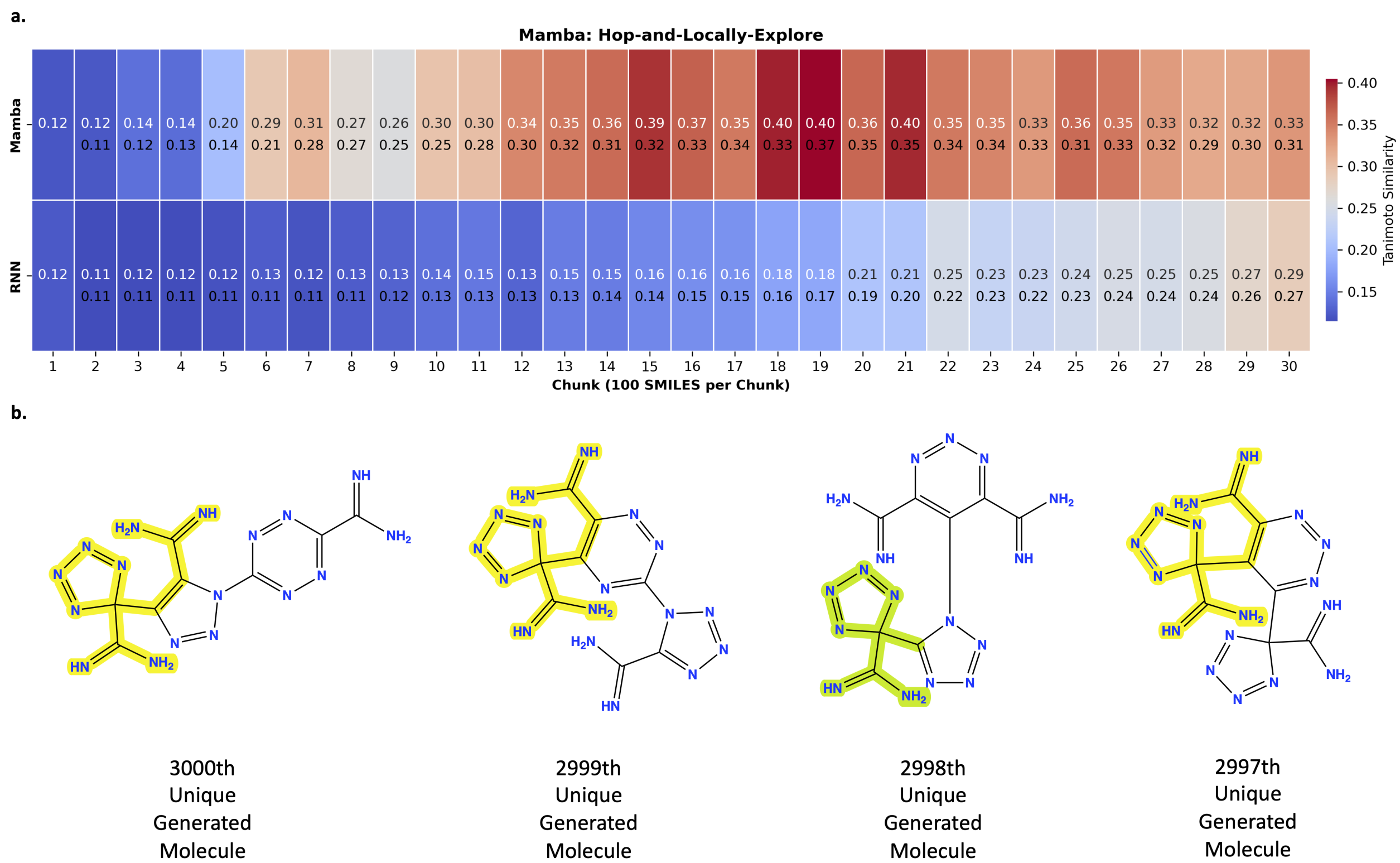

Figure S2: Mamba (batch size 16, augmentation rounds 10) and baseline Augmented Memory (batch size 64, augmentation rounds 2) which is labelled as **RNN**. **a.** 3,000 oracle budget test experiment **chunked into 100 SMILES**. Mamba exhibits a "hop-and-locally-exploit" behavior where the intra-chunk Tanimoto similarity (top values) are higher than RNN. The bottom value is the inter-chunk similarity. **b.** Qualitative examples of unique molecules generated at adjacent epochs. Many substructures are shared and the model generates in the local neighborhood. Yellow highlights are exact substructures shared while green indicates a portion.

We now focus on Mamba (batch size 16, augmentation rounds 10) and present additional results to qualitatively and quantitatively demonstrate "hop-and-locally-exploit" behavior. Firstly, we supplement the main text Figure 2e. The figure shows the intra- and inter-chunk similarities across chunks of generated molecules. Specifically, the test experiment was run with an oracle budget of 3,000 and this generated set is chunked. To provide a more granular inspection into the generative behavior, we chunk this set into 30 chunks (each 100 SMILES) instead of 10 chunks (each 300 SMILES) in the main text. Mamba (batch size 16, augmentation rounds) exhibits notably higher intra-chunk similarity and even inter-chunk similarity at this more granular chunking level (Fig-

ure S2a). We further supplement these quantitative results with a qualitative inspection. Looking at **unique** molecules generated at adjacent epochs, common substructures are shared (Figure S2b highlights), displaying a "neighborhood-like" exploration.

3.1 Is "Hop-and-locally-exploit" *Always* Good?

The results in the main text and this section so far provide evidence that Mamba with batch size 16 and 10 augmentation rounds exhibits local exploration behavior. We hypothesize that sample efficiency improves because "similar molecules, on average, exhibit similar properties". But is this always true? In the test experiment, it is straightforward to see that this indeed holds true. Cross-referencing Figure S2b, small changes to the molecular graphs should still display high polar surface area which is the objective. However, oracles we care about are physics-based simulations. In the main text results and later in this Supplementary Information, we show that this behavior is beneficial for sample efficiency. The physics-based oracles used in this work are AutoDock Vina [52] and QuickVina 2 [53] which run molecular docking. The question we pose is: are these oracles *too permissive*? Such that the optimization landscape is smooth [54]. As we push towards higher-fidelity oracles such as QM/MM and free energy simulations [45], it is expected that they will be more stringent and demand more specificity. This means that the current hypothesis of "similar molecules, on average, exhibit similar properties" may be loosened. Whether this turns out to be detrimental or not in high-fidelity oracle settings remains to be empirically tested which we leave for future work. By characterizing the behavior of Saturn and understanding what *exactly* Augmented Memory is doing, it is possible to adapt the current model accordingly. For example, decreasing augmentation rounds relaxes the "hop-and-locally-exploit" behavior, which *could* be advantageous for high-fidelity oracles.

3.2 Genetic Algorithm Loosens "hop-and-locally-exploit Behavior"

In our investigations to applying a GA on the replay buffer, we show that while sample efficiency does not improve, diversity recovers. To quantitatively show why, we plot the chunk similarity for an experiment from Part 2 on the parp1 target with and without the GA activated (Figure S3). The Mamba model in both cases uses batch size 16 and 10 augmentation rounds. With the GA

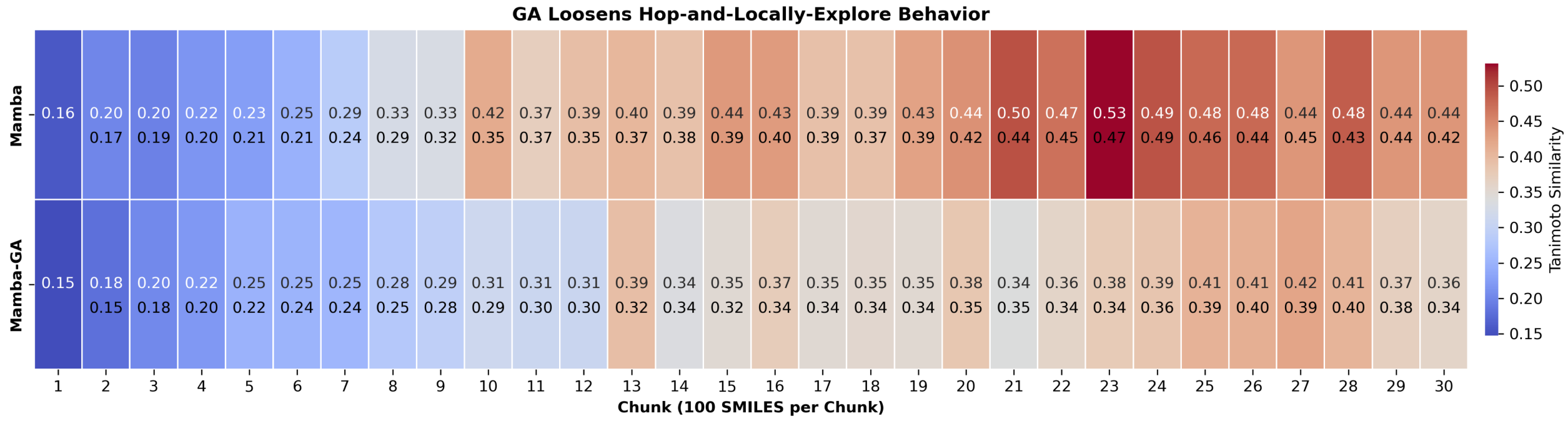

Figure S3: Mamba (batch size 16, augmentation rounds 10) with and without GA [13] activated. The experiment is the Part 2 MPO objective (docking against parp1).

activated, the intra-chunk similarities decrease, thus loosening the local exploitation behavior and is the reason why diversity recovers.

4 Transferability of Sample Efficiency to Physics-based Oracles

In this section, we demonstrate that Saturn's sample efficiency transfers to an MPO objective involving docking against targets related to neurodegeneration (**DRD2** [55] and **AChE** [56]) and inflammation (**MK2 kinase** [57]). The optimization objective is to constrain MW $<$ 500 Da, maximize the quantitative estimate of drug-likeness (QED) [31], and minimize AutoDock Vina [52] docking score (see Supplementary Information 4.2 for details on the docking protocol). All experiments were run across 10 seeds (0-9 inclusive) and with a 1,000 oracle budget (number of unique molecules assessed).

4.1 Quantitative Results

We compare Saturn (with and without GA) to baseline Augmented Memory [1] using the Yield and OB metrics. Saturn generates more high reward molecules and faster, given the fixed oracle budget (Table S21). This holds even for the more challenging MK2 kinase target where all methods have diminished performance. Furthermore, in agreement with the results from the test experiments, adding a GA on the buffer does not improve sample efficiency but recovers diversity, which can be useful in certain cases.

4.2 Docking Protocol

All protein receptor structures were pre-processed from the raw PDB. The following were removed: Duplicate protein chains and duplicate ligands, co-factors, ions, and all waters. Next, Schrödinger's Protein Preparation Wizard [59, 60] with default parameters was used to pre-process the structure. PROPKA hydrogen-bond network optimization was performed at pH 7.4 and energy minimization with OPLS3e force-field [61]. Below are details on the docking grids generated from the pre-processed PDBs.

DRD2 - Dopamine Type 2 Receptor. The PDB ID is 6CM4[55] and the docking grid was centered at (x, y, z) = (9.93, 5.85, -9.58).

MK2 - MK2 Kinase. The PDB ID is 3KC3[57] and the docking grid for the extracted

monomer was centered at (x, y, z) = (-61.62, 30.31, -21.9).

AChE - Acetylcholinesterase. The PDB ID is 1EVE[56] and the docking grid was centered at (x, y, z) = (2.78, 64.38, 67.97).

Docking. The search box for all grids was 15Å x 15Å x 15Å and docking was executed through DockStream [62]. All generated molecules were first embedded using the RDKit Universal Force Field (UFF) [63] with the maximum convergence set to 600 iterations. Docking was parallelized over 16 CPU cores (since the generative model's batch size was 16). The cores were Intel(R) Xeon(R) Platinum 8360Y processors.

Table S21: Docking MPO with 1,000 oracle budget. Baseline is vanilla Augmented Memory [1]. IntDiv1 [51] is the internal diversity, Scaffolds is the number of unique Bemis-Murcko [58] scaffolds, OB is Oracle Burden (oracle calls required to generate *N* unique molecules). All metrics are computed at the 0.8 reward threshold. A 'Preliminary Check' was used in these experiments, such that if a generated molecule was larger than 500 MW, then it was discarded and not sent to the docking oracle. The number in parentheses in the OB statistics represents how many runs out of 10 were successful. The mean and standard deviation across 10 seeds (0-9 inclusive) is reported. Comparing Augmented Memory and Saturn 'Yield' results, the p-values at the 95% confidence level are (rounded to 4 decimal points): DRD2 = 0.0000 and AChE = 0.0000 using a two-sided Welch's t-test. The MK2 Yield distributions are not normal and therefore, we use a two-sided Mann-Whitney U test to probe whether the distributions are different. The p-value at the 95% confidence level (also rounded to 4 decimal points) is: MK2 = 0.0007.

Target	Model	Yield (↑)	IntDiv1 (↑)	Scaffolds (↑)	OB 1 (↓)	OB 10 (↓)	OB 100 (↓)
DRD2	Augmented Memory	22 ± 7	0.774 ± 0.019	22 ± 7	$143 \pm 75(10)$	$733 \pm 120(10)$	Failed
	**Saturn-Mamba**	369 ± 62	0.671 ± 0.050	310 ± 70	$93 \pm 53(10)$	$391 \pm 56(10)$	$663 \pm 55(10)$
	Saturn-Mamba-GA	209 ± 55	0.745 ± 0.041	189 ± 57	$96 \pm 56(10)$	$403 \pm 75(10)$	$806 \pm 84(10)$
AChE	Augmented Memory	173 ± 19	0.843 ± 0.009	170 ± 18	$57 \pm 2(10)$	$189 \pm 52(10)$	$776 \pm 58(10)$
	**Saturn-Mamba**	480 ± 79	0.757 ± 0.020	400 ± 96	$32 \pm 24(10)$	$185 \pm 82(10)$	$508 \pm 80(10)$
	Saturn-Mamba-GA	343 ± 57	0.809 ± 0.013	287 ± 50	$32 \pm 25(10)$	$187 \pm 80(10)$	$565 \pm 80(10)$
MK2	Augmented Memory	0.2 ± 0.4	—	0.2 ± 0.4	$836 \pm 186(2)$	Failed	Failed
	**Saturn-Mamba**	14.9 ± 14.1	0.454 ± 0.212	14.1 ± 13.2	$677 \pm 186(9)$	$861 \pm 108(6)$	Failed
	Saturn-Mamba-GA	6.1 ± 6.5	0.415 ± 0.202	5.5 ± 5.5	$678 \pm 140(9)$	$911 \pm 11(2)$	Failed

4.3 Additional Results

In Table S21 at the start of this section, results were shown at the 0.8 reward threshold. In this subsection, we also show results for Saturn-RNN (batch size 16, augmentation rounds 10) and for the 0.7 reward threshold (Tables S22 and S23). At the 0.7 reward threshold, Saturn-RNN's performance is almost identical to Saturn. However, at the 0.8 reward threshold, Saturn-Mamba is more performant. We highlight that although at times, the difference may be small, it can be highly practically relevant when using expensive oracles, e.g., 50 docking calls may be inconsequential but 50 molecular dynamics simulations can be costly. Both Saturn-RNN and Saturn outperform baseline Augmented Memory. Finally, adding a GA on top of Saturn recovers diversity but sample efficiency decreases.

4.4 Compute Time

Due to insufficient GPU resources, we ran all experiments in this section on CPU. Averaged across all targets and across all 10 replicates, the wall time were as follows: 172 minutes (approximately 3 hours) for Augmented Memory, 246 minutes (approximately 4 hours) for Saturn-RNN, 1,426 minutes (approximately 24 hours) for Saturn, and 1,111 minutes (approximately 18.5 hours) for Saturn-GA. There is such a large discrepancy in run time due to repeated SMILES (which do not impose additional oracle calls) that still require backpropagation. Moreover, the runs with Mamba take so much longer because the GPU implementation is highly optimized. When run on GPU, the difference in wall time between Saturn-RNN and Saturn (Mamba) are not significant.

Table S22: Docking MPO with 1,000 oracle budget. Baseline is vanilla Augmented Memory [1]. All metrics are computed at the 0.7 reward threshold. IntDiv1 is the internal diversity, scaffolds is the number of unique Bemis-Murcko scaffolds, OB is Oracle Burden (oracle calls required to generate *N* unique molecules). The number in parentheses in the OB statistics represent how many runs out of 10 were successful. The mean and standard deviation across 10 seeds (0-9 inclusive) is reported. Saturn-RNN is RNN with batch size 16 and augmentation rounds 10.

Model	Yield (↑)	IntDiv1 (↑)	Scaffolds (↑)	OB 1 (↓)	OB 10 (↓)	OB 100 (↓)
DRD2						
Baseline	630 ± 45	0.858 ± 0.006	585 ± 43	$57 \pm 2(10)$	$57 \pm 2(10)$	$279 \pm 32(10)$
Saturn-RNN	818 ± 22	0.821 ± 0.011	671 ± 56	$14 \pm 1(10)$	$31 \pm 6(10)$	$219 \pm 16(10)$
Saturn-Mamba	850 ± 23	0.784 ± 0.015	677 ± 51	$14 \pm 1(10)$	$35 \pm 7(10)$	$199 \pm 20(10)$
Saturn-Mamba-GA	804 ± 26	0.817 ± 0.022	685 ± 56	$14 \pm 1(10)$	$35 \pm 7(10)$	$199 \pm 19(10)$
MK2 Kinase						
Baseline	431 ± 32	0.863 ± 0.005	406 ± 26	$57 \pm 2(10)$	$74 \pm 26(10)$	$396 \pm 37(10)$
Saturn-RNN	704 ± 25	0.833 ± 0.013	525 ± 32	$14 \pm 1(10)$	$43 \pm 9(10)$	$282 \pm 19(10)$
Saturn-Mamba	702 ± 43	0.811 ± 0.022	519 ± 69	$17 \pm 6(10)$	$52 \pm 12(10)$	$282 \pm 31(10)$
Saturn-Mamba-GA	636 ± 29	0.827 ± 0.019	506 ± 68	$17 \pm 6(10)$	$52 \pm 12(10)$	$291 \pm 31(10)$
AChE						
Baseline	801 ± 27	0.867 ± 0.006	759 ± 30	$57 \pm 2(10)$	$57 \pm 2(10)$	$201 \pm 29(10)$
Saturn-RNN	909 ± 21	0.842 ± 0.006	772 ± 73	$14 \pm 1(10)$	$25 \pm 6(10)$	$163 \pm 19(10)$
Saturn-Mamba	906 ± 15	0.816 ± 0.014	742 ± 76	$14 \pm 1(10)$	$27 \pm 4(10)$	$158 \pm 13(10)$
Saturn-Mamba-GA	874 ± 21	0.841 ± 0.008	732 ± 48	$14 \pm 1(10)$	$27 \pm 4(10)$	$158 \pm 14(10)$

Table S23: Docking MPO with 1,000 oracle budget. Baseline is vanilla Augmented Memory [1]. All metrics are computed at the 0.8 reward threshold. IntDiv1 is the internal diversity, scaffolds is the number of unique Bemis-Murcko scaffolds, OB is Oracle Burden (oracle calls required to generate *N* unique molecules). The number in parentheses in the OB statistics represent how many runs out of 10 were successful. The mean and standard deviation across 10 seeds (0-9 inclusive) is reported. Saturn-RNN is RNN with batch size 16 and augmentation rounds 10.

Model	Yield (↑)	IntDiv1 (↑)	Scaffolds (↑)	OB 1 (↓)	OB 10 (↓)	OB 100 (↓)
DRD2						
Baseline	22 ± 7	0.774 ± 0.019	22 ± 7	$143 \pm 75(10)$	$733 \pm 120(10)$	Failed
Saturn-RNN	185 ± 40	0.745 ± 0.022	148 ± 47	$128 \pm 94(10)$	$440 \pm 72(10)$	$854 \pm 63(10)$
Saturn-Mamba	369 ± 62	0.671 ± 0.050	310 ± 70	$93 \pm 53(10)$	$391 \pm 56(10)$	$663 \pm 55(10)$
Saturn-Mamba-GA	209 ± 55	0.745 ± 0.041	189 ± 57	$96 \pm 56(10)$	$403 \pm 75(10)$	$806 \pm 84(10)$
MK2 Kinase						
Baseline	0.2 ± 0.4	—	0.2 ± 0.4	$836 \pm 186(2)$	Failed	Failed
Saturn-RNN	2.5 ± 3.4	0.414 ± 0.213	2.5 ± 3.4	$642 \pm 91(6)$	$999 \pm 0(1)$	Failed
Saturn-Mamba	14.9 ± 14.1	0.454 ± 0.212	14.1 ± 13.2	$677 \pm 186(9)$	$861 \pm 108(6)$	Failed
Saturn-Mamba-GA	6.1 ± 6.5	0.415 ± 0.202	5.5 ± 5.5	$678 \pm 140(9)$	$911 \pm 11(2)$	Failed
AChE						
Baseline	173 ± 19	0.843 ± 0.009	170 ± 18	$57 \pm 2(10)$	$189 \pm 52(10)$	$776 \pm 58(10)$
Saturn-RNN	419 ± 38	0.804 ± 0.019	338 ± 55	$21 \pm 11(10)$	$165 \pm 60(10)$	$531 \pm 36(10)$
Saturn-Mamba	480 ± 79	0.757 ± 0.020	400 ± 96	$32 \pm 24(10)$	$185 \pm 82(10)$	$508 \pm 80(10)$
Saturn-Mamba-GA	343 ± 57	0.809 ± 0.013	287 ± 50	$32 \pm 25(10)$	$187 \pm 80(10)$	$565 \pm 80(10)$

5 Part 2: Benchmarking the Saturn Framework and Demonstrating Enhanced Optimization

In this section, we detail how Saturn was pre-trained for benchmarking, the procedure we followed to reproduce GEAM [43], and additional results. We ensured exact reproducibility by using GEAM's official code: https://anonymous.4open.science/r/GEAM-45EF (since the pre-print version of the Saturn work, this GEAM repository, originally used for conference anonymous submission has expired. The GEAM codebase is now available here: https://github.com/SeulLee05/GEAM). For running Saturn with GEAM's objective function, all the oracle code was taken, without modification, from the same repository. Additionally, we follow GEAM and use Morgan fingerprints with radius 2 and 1024 bits for the diversity metric calculations (IntDiv1 [51] and #Circles [64])

5.1 Saturn ZINC 250k Pre-training

GEAM pre-trained on ZINC 250k [49] and provide the dataset in their repository. We used this dataset as is for Saturn pre-training (Mamba model).

The pre-training parameters were:

1. Training steps = 50 (each training step entails a full pass through the dataset)
2. Seed = 0
3. Batch size = 512
4. Learning rate = 0.0001
5. Train with SMILES randomization [39] (all SMILES in each batch was randomized)

Mamba model:

1. Vocabulary size = 66 (including the 2 added tokens for <START> and <END>)
2. 5,272,832 parameters

3. Used checkpoint from epoch 50 (NLL = 28.10, Validity (10k) = 95.2%)

All Saturn experiments were run on a single workstation equipped with an NVIDIA RTX A6000 GPU and AMD Ryzen 9 5900X 12-Core CPU. The total run time for Saturn across all targets was 41.5 hours (total of 50 runs: 5 targets, 10 seeds each).

5.2 Reproducing GEAM's Results

We followed the instructions directly in GEAM's README: https://anonymous.4open.science/r/GEAM-45EF/README.md. We trained the FGIB with seed 0. Everything else was run with their default parameters. In the original work, 3 replicates were run but the seeds were not specified. In our comparisons, we run GEAM across 10 seeds (0-9 inclusive) using an NVIDIA V100 GPU with a Xeon-Gold processor (2.1 GHz and 20 cores) CPU. The reason why a different GPU was used in GEAM experiments compared to Saturn is due to CUDA compatibility in GEAM's code. For GEAM, the wall times were:

1. **parp1**: 3.02±0.19 hours
2. **fa7**: 3.38±0.04 hours
3. **5ht1b**: 3.17±0.08 hours
4. **braf**: 3.02±0.19 hours
5. **jak2**: 3.28±0.04 hours

Except for **parp1**, the wall times are the mean and standard deviation across 10 seeds. For **parp1**, the wall times are across 7 seeds (3-9 inclusive). Seeds 0-2 inclusive were run on CPU due to insufficient GPU resources. CPU runs take much longer so we only report GPU times.

5.3 GEAM's MPO Objective

GEAM optimized for the following objective:

$$R(x) = \widehat{DS}(x) \times QED(x) \times \widehat{SA}(x) \in [0, 1] \tag{17}$$

$\widehat{DS}$ is the normalized QuickVina 2 [53] docking score (Eq. 18), QED [31] is the quantitative estimate of drug-likeness, and $\widehat{SA}$ is the normalized synthetic accessibility score [32] (Eq. 19).

$$\widehat{DS} = -\frac{\text{DS}}{20} \tag{18}$$

$$\widehat{SA} = \frac{10 - \text{SA}}{9} \tag{19}$$

5.4 Saturn-Tanimoto

In GEAM [43], the "Novel" in **Novel Hit Ratio** enforces molecules to possess $<$ 0.4 Tanimoto similarity to ZINC 250k [49]. GEAM achieves this by use of their fragment assembly *and* genetic algorithm which directly uses GraphGA [13]. The crossover and mutation operations promote diversity[42]. Otherwise, generative models are pre-trained to model the training data distribution. This means that generated molecules would not necessarily be particularly dissimilar to the training data, especially if the training data actually possesses "good" molecules already. By virtue of pre-training on a selected dataset, we implicitly assume that the pre-training dataset is "good" for our task, otherwise, we probably should not pre-train on this data. This is the rationale on why ChEMBL [48] and ZINC 250k [49] are popular pre-training datasets: they contain bio-active molecules. To satisfy GEAM's "Novel" criterion, we take the base Saturn model and first teach it to generate molecules that are dissimilar to the ZINC 250k dataset which was used for pre-training. The objective function is then defined as minimizing the max Tanimoto similarity to any molecule in ZINC 250k. This experiment was run with an oracle budget of 1,500 and took about 10 minutes. The resulting **Saturn-Tanimoto** model generates molecules with low Tanimoto similarity to ZINC 250k. Starting from this model, we run GEAM's case study and the results from this are reported in the main text and here in the Supplementary Information. We finally note that this criterion is somewhat arbitrary and we do it so we can exactly match GEAM's experiments.

5.5 Quantitative Supplementary Results

In this section, we present supplementary benchmarking results and show additional results for Saturn-GA.

Table S24: Hit Ratio (%). Results are from Lee et al. [65] except GEAM, datasets, Genetic GFN, and the Saturn framework which we ran across 10 seeds (0-9 inclusive). The mean and standard deviation are reported. Best results (statistically significant at the 95% confidence level using a two-sided Welch's t-test) are bolded. Comparing GEAM and Saturn (Framework) results, the p-values are (rounded to 4 decimal points): parp1 = 0.0687, fa7 = 0.1078, 5ht1b = 0.0000, braf = 0.03236, jak2 = 0.0039.

Method	Target Protein				
	parp1	fa7	5ht1b	braf	jak2
**Datasets**					
ZINC 250k [49]	3.993 ± 0.355	1.097 ± 0.192	24.260 ± 0.622	1.020 ± 0.193	6.183 ± 0.344
ChEMBL 33 [48]	6.077 ± 0.453	1.830 ± 0.240	24.163 ± 0.715	2.073 ± 0.181	9.013 ± 0.562
**Generative Models**					
REINVENT [3]	4.693 ± 1.776	1.967 ± 0.661	26.047 ± 2.497	2.207 ± 0.800	5.667 ± 1.067
JT-VAE [66]	3.200 ± 0.348	0.933 ± 0.152	18.044 ± 0.747	0.644 ± 0.157	5.856 ± 0.204
GraphAF [67]	0.822 ± 0.113	0.011 ± 0.016	6.978 ± 0.952	1.422 ± 0.556	1.233 ± 0.284
MORLD [68]	0.047 ± 0.050	0.007 ± 0.013	0.893 ± 0.758	0.047 ± 0.040	0.227 ± 0.118
HierVAE [69]	1.180 ± 0.182	0.033 ± 0.030	0.740 ± 0.371	0.367 ± 0.187	0.487 ± 0.183
GraphDF [70]	0.044 ± 0.031	0.000 ± 0.000	0.000 ± 0.000	0.011 ± 0.016	0.011 ± 0.016
FREED [71]	4.860 ± 1.415	1.487 ± 0.242	14.227 ± 5.116	2.707 ± 0.721	6.067 ± 0.790
FREED-QS [71]	5.960 ± 0.902	1.687 ± 0.177	23.140 ± 2.422	3.880 ± 0.623	7.653 ± 1.373
LIMO [72]	0.456 ± 0.057	0.044 ± 0.016	1.200 ± 0.178	0.278 ± 0.134	0.711 ± 0.329
GDSS [73]	2.367 ± 0.316	0.467 ± 0.112	6.267 ± 0.287	0.300 ± 0.198	1.367 ± 0.258
MOOD [65]	7.260 ± 0.764	0.787 ± 0.128	21.427 ± 0.502	5.913 ± 0.311	10.367 ± 0.616
Augmented Memory [1]	16.966 ± 3.224	2.637 ± 0.860	52.016 ± 2.302	8.307 ± 1.714	21.548 ± 4.938
Genetic GFN [14]	8.420 ± 1.794	1.532 ± 0.272	36.122 ± 2.568	3.375 ± 0.634	10.818 ± 1.636
GEAM [43]	$\mathbf{45.158 \pm 2.408}$	$\mathbf{20.552 \pm 2.357}$	47.664 ± 1.198	30.444 ± 1.610	46.129 ± 2.073
**Ours**					
Saturn (Framework)	$\mathbf{57.981 \pm 18.537}$	$\mathbf{14.527 \pm 9.961}$	$\mathbf{68.185 \pm 3.400}$	$\mathbf{38.999 \pm 10.114}$	$\mathbf{60.827 \pm 11.502}$
Saturn-GA	55.597 ± 5.617	16.711 ± 6.761	63.112 ± 4.316	34.284 ± 10.345	58.625 ± 6.982
Saturn-Tanimoto	77.674 ± 7.127	23.119 ± 6.852	78.433 ± 1.029	30.258 ± 12.315	83.012 ± 6.768

Hit Ratio (%). Table S24 shows the Hit Ratio (%) results. Random sampling of 3,000 molecules from common datasets (ZINC 250k [49] and ChEMBL 33 [48]) are included as baselines. The results show that only GEAM [43] and Saturn outperform these baselines with both

Table S25: Strict Hit Ratio (%) (QED > 0.7 and SA < 3) additional results. GEAM and Saturn results are across 10 seeds (0-9 inclusive). OB is Oracle Burden (oracle calls required to generate *N* unique molecules). The number in parentheses in the OB statistics represent how many runs out of 10 were successful. The mean and standard deviation are reported.

Method	Target Protein				
	parp1	fa7	5ht1b	braf	jak2
**GEAM [43] - Presented in Main Text**					
Strict Hit Ratio (↑)	6.510 ± 1.087	2.106 ± 0.958	8.719 ± 0.903	3.685 ± 0.524	7.944 ± 1.157
IntDiv1 (↑)	0.760 ± 0.016	0.702 ± 0.042	0.792 ± 0.016	0.745 ± 0.023	0.758 ± 0.021
#Circles (↑)	14 ± 1	6 ± 1	23 ± 2	11 ± 1	17 ± 2
OB (1) (↓)	$250 \pm 157(10)$	$433 \pm 209(10)$	$114 \pm 112(10)$	$355 \pm 96(10)$	$230 \pm 117(10)$
OB (10) (↓)	$743 \pm 52(10)$	$1446 \pm 404(10)$	$531 \pm 38(10)$	$892 \pm 144(10)$	$537 \pm 70(10)$
OB (100) (↓)	$2106 \pm 202(10)$	$2927 \pm 0(1)$	$1527 \pm 110(10)$	$2674 \pm 163(6)$	$1606 \pm 218(10)$
**Saturn (Framework) - Presented in Main Text**					
Strict Hit Ratio	55.102 ± 18.027	13.887 ± 9.723	64.730 ± 3.717	37.250 ± 9.615	55.903 ± 13.613
IntDiv1 (↑)	0.585 ± 0.049	0.583 ± 0.067	0.675 ± 0.021	0.587 ± 0.042	0.628 ± 0.036
#Circles (↑)	5 ± 1	3 ± 1	17 ± 3	4 ± 1	8 ± 2
OB (1) (↓)	$139 \pm 96(10)$	$352 \pm 206(10)$	$21 \pm 7(10)$	$291 \pm 143(10)$	$88 \pm 56(10)$
OB (10) (↓)	$518 \pm 92(10)$	$924 \pm 247(10)$	$105 \pm 23(10)$	$581 \pm 123(10)$	$348 \pm 96(10)$
OB (100) (↓)	$956 \pm 259(10)$	$1776 \pm 551(10)$	$441 \pm 44(10)$	$1057 \pm 187(10)$	$785 \pm 191(10)$
**Saturn-GA (ours) - Newly presented here**					
Strict Hit Ratio	47.146 ± 4.952	13.187 ± 6.340	53.055 ± 3.764	28.377 ± 9.703	49.528 ± 5.463
IntDiv1 (↑)	0.649 ± 0.024	0.627 ± 0.040	0.715 ± 0.023	0.618 ± 0.048	0.666 ± 0.038
#Circles (↑)	8 ± 1	5 ± 1	21 ± 4	6 ± 1	11 ± 3
OB (1) (↓)	$121 \pm 71(10)$	$350 \pm 203(10)$	$20 \pm 6(10)$	$242 \pm 194(10)$	$91 \pm 43(10)$
OB (10) (↓)	$467 \pm 114(10)$	$912 \pm 168(10)$	$110 \pm 36(10)$	$582 \pm 177(10)$	$375 \pm 120(10)$
OB (100) (↓)	$937 \pm 136(10)$	$1852 \pm 349(10)$	$499 \pm 85(10)$	$1266 \pm 486(10)$	$861 \pm 123(10)$

methods performing similarly overall. With the exception of a few targets where performance differs (significant at the 95% confidence level using a two-sided Welch's t-test), Saturn notably exhibits higher variance which is expected given the small batch size (16). One way to mitigate high variance is to use a larger batch size, as this makes the approximation for the expected reward less noisy. Next, we show that the Saturn-Tanimoto Agent displays notably high Hit Ratios but do not present this in the main results as the purpose of the Tanimoto Agent is to generate hits that have less than 0.4 Tanimoto similarity to the ZINC 250k [49] training dataset. It is difficult to predict *a priori* a favorable chemical space to move the Agent. However, this result is interesting as it suggests that this simple additional pre-training which took minutes via curriculum

learning (CL), makes the Agent more suited for the docking tasks. Finally, we show that using the GA (Saturn-GA) is a straightforward solution to maintain diversity. From Part 1 and Part 2 experiments, activating the GA comes at the expense of some sample efficiency but interestingly, this is not the case here (Table S25). Moreover, Saturn-GA also decreases variance in this case study (Table S24). Based on these results, it would actually be beneficial to activate the GA in this case, but it is difficult to know *a priori* the best configuration, thus we report the out-of-the-box hyperparameters (without GA) in the main text based on tuning on the test experiment in Part 1.

Strict Novel Hit Ratio (%). Table S26 shows the Strict Novel Hit Ratio (%) results with all additional metrics, mirroring the main text table. Similar to the main text results, Saturn-Tanimoto Agent generates significantly more molecules passing the strict filter and also much faster (fewer oracle calls). However, the diversity notably drops (much more than the Mamba Agent without Tanimoto distance training presented in the main text). However, diversity is particularly low. We first not that when moving to high-fidelity oracles where satisfying the objective function equates to higher true positive hit rates, low diversity need not be detrimental. We additionally run an experiment with the GA activated and we see diversity recovers, but is still notably lower than GEAM. Moreover, the sample efficiency drops notably here compared to without GA, but is still much more performant than GEAM in finding hits faster. Finally, to recover more diversity, one could make the Diversity Filter [16] more stringent. In this work, a bucket size of 10 was used (allow 10 of the same scaffold to be generated before truncating the reward to 0). Decreasing the bucket size to 5 or even lower, may recover more diversity.

Novel Hit Ratio (%) Across Tanimoto Thresholds. In the analyses thus far, we follow Lee et al. [43] and define generated molecules as "Novel" if their maximum Tanimoto similarity to the ZINC 250k [49] pre-training data is less than 0.4. In this section, we perform the same analysis through a grid of Tanimoto similarity thresholds: 0.1, 0.2, 0.3, 0.4, 0.5, 0.6, 0.7, 0.8, 0.9, 1.0. Figure S4 shows that Saturn-Tanimoto generally has a higher novel hit ratio across all Tanimoto thresholds and across both the default and strict filters.

ZINC 250k Dataset vs. GEAM vs. Saturn Reward Distributions. Figure S5 supplements the main text results and shows that the reward of molecules randomly sampled from the ZINC 250k [49] pre-training data is similar to GEAM generated molecules. The reason for this is that the ZINC sampled molecules possess more favorable QED and SA scores, on average. Since the

Table S26: Strict Novel Hit Ratio (%) (QED > 0.7 and SA < 3). GEAM and Saturn results are across 10 seeds (0-9 inclusive). OB is Oracle Burden (oracle calls required to generate *N* unique molecules). The number in parentheses in the OB statistics represent how many runs out of 10 were successful. The mean and standard deviation are reported.

Method	Target Protein				
	parp1	fa7	5ht1b	braf	jak2
**GEAM** [43]					
Strict Hit Ratio (↑)	4.018 ± 0.849	1.676 ± 0.836	5.338 ± 0.789	2.621 ± 0.464	5.930 ± 1.151
IntDiv1 (↑)	0.762 ± 0.018	0.702 ± 0.046	0.786 ± 0.019	0.746 ± 0.026	0.758 ± 0.025
#Circles (↑)	12 ± 2	6 ± 1	21 ± 2	10 ± 2	15 ± 1
OB (1) (↓)	$319 \pm 175(10)$	$502 \pm 209(10)$	$253 \pm 159(10)$	$419 \pm 102(10)$	$242 \pm 124(10)$
OB (10) (↓)	$857 \pm 86(10)$	$1625 \pm 380(10)$	$689 \pm 77(10)$	$1047 \pm 136(10)$	$616 \pm 83(10)$
OB (100) (↓)	$2633 \pm 202(9)$	Failed	$2221 \pm 224(10)$	$2942 \pm 0(1)$	$2005 \pm 268(10)$
**Saturn-Tanimoto (ours)**					
Strict Novel Hit Rate	47.405 ± 8.593	17.130 ± 5.538	50.445 ± 6.334	18.228 ± 9.438	45.185 ± 13.321
IntDiv1 (↑)	0.591 ± 0.029	0.596 ± 0.030	0.554 ± 0.032	0.515 ± 0.039	0.562 ± 0.040
#Circles (↑)	2 ± 1	1 ± 0	2 ± 1	1 ± 0	1 ± 0
OB (1) (↓)	$26 \pm 17(10)$	$98 \pm 53(10)$	$15 \pm 0(10)$	$164 \pm 137(10)$	$18 \pm 7(10)$
OB (10) (↓)	$177 \pm 38(10)$	$320 \pm 69(10)$	$31 \pm 5(10)$	$388 \pm 156(10)$	$70 \pm 13(10)$
OB (100) (↓)	$562 \pm 94(10)$	$1051 \pm 251(10)$	$223 \pm 50(10)$	$1041 \pm 585(9)$	$402 \pm 196(10)$
**Saturn-Tanimoto-GA (ours)**					
Strict Novel Hit Ratio	29.801 ± 11.603	10.915 ± 5.740	40.261 ± 8.168	17.845 ± 7.943	37.498 ± 11.200
IntDiv1 (↑)	0.615 ± 0.040	0.582 ± 0.039	0.608 ± 0.041	0.634 ± 0.041	0.601 ± 0.034
#Circles (↑)	2 ± 0	2 ± 1	4 ± 1	3 ± 1	3 ± 0
OB (1) (↓)	$36 \pm 38(10)$	$216 \pm 232(10)$	$15 \pm 0(10)$	$181 \pm 122(10)$	$17 \pm 5(10)$
OB (10) (↓)	$205 \pm 65(10)$	$545 \pm 275(10)$	$27 \pm 5(10)$	$472 \pm 135(10)$	$96 \pm 13(10)$
OB (100) (↓)	$703 \pm 113(10)$	$1490 \pm 460(9)$	$272 \pm 39(10)$	$1367 \pm 561(10)$	$480 \pm 84(10)$

reward function (Eq. 17) places equal weighting on these properties, GEAM generated molecules possess similar reward values despite having better docking scores.

We next quantitatively show that a reason GEAM generates molecules with slightly lower docking scores than Saturn (though the distributions are similar) is because the generated molecules are larger with lower ligand efficiency (docking score / number of heavy atoms). This is in agree-

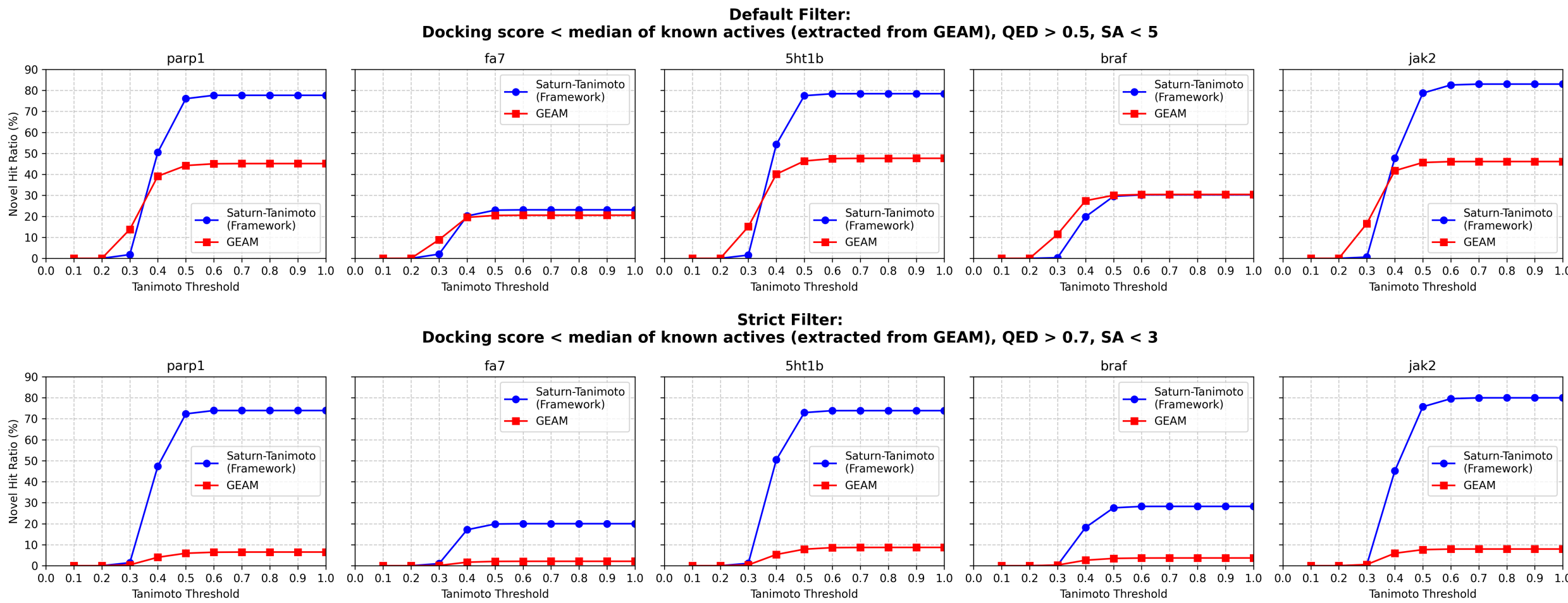

Figure S4: Novel hit ratios across Tanimoto thresholds comparing GEAM and Saturn-Tanimoto.

ment with GEAM's QED distribution being notably lower than Saturn's, as QED constrains the molecular weight.

Table S27: Comparison of Hit Ratio (%), Ligand Efficiency, and Molecular Weight between GEAM and Saturn across five target proteins. Mean and standard deviation are reported.

Target	Hit Ratio (%)		Ligand Efficiency		Molecular Weight	
	GEAM	Saturn	GEAM	Saturn	GEAM	Saturn
parp1	45.158 ± 2.408	57.981 ± 18.537	0.374 ± 0.005	0.405 ± 0.011	416.81 ± 4.94	371.21 ± 7.85
fa7	20.552 ± 2.357	68.185 ± 3.400	0.302 ± 0.006	0.408 ± 0.013	417.98 ± 7.73	353.17 ± 13.71
5ht1b	47.664 ± 1.198	38.999 ± 10.114	0.380 ± 0.006	0.406 ± 0.017	394.92 ± 3.63	345.21 ± 11.49
braf	30.444 ± 1.610	14.527 ± 9.961	0.384 ± 0.004	0.343 ± 0.011	404.93 ± 3.15	365.96 ± 17.41
jak2	46.129 ± 2.073	60.827 ± 11.502	0.363 ± 0.005	0.389 ± 0.017	395.08 ± 3.59	347.08 ± 12.79

Table S27 summarizes the ligand efficiency and molecular weight results comparing GEAM and Saturn. On average, larger molecules will possess a more favourable docking score. Saturn generates smaller molecules than GEAM and generally has a higher ligand efficiency (except for braf).

Table S28: Molecule and Bemis-Murcko scaffold novelty metrics across 5 targets for the Saturn and Saturn Tanimoto frameworks. The pre-training data was ZINC 250k.

	parp1	fa7	5ht1b	braf	jak2
**Saturn Framework**					
Molecule Novelty	1.000 ± 0.0	0.999 ± 0.001	0.999 ± 0.000	0.999 ± 0.000	0.999 ± 0.001
Scaffold Novelty	0.880 ± 0.026	0.853 ± 0.017	0.873 ± 0.032	0.883 ± 0.026	0.865 ± 0.028
**Saturn Tanimoto Framework**					
Molecule Novelty	1.000 ± 0.000	1.000 ± 0.000	1.0 ± 0.000	1.000 ± 0.000	1.000 ± 0.000
Scaffold Novelty	0.999 ± 0.001	0.999 ± 0.0	0.999 ± 0.0	0.999 ± 0.001	0.999 ± 0.001

5.6 Novelty Metrics

In this subsection, we report the molecule novelty: how many of the generated molecules are in the ZINC 250k [49] pre-training data and the scaffold novelty: how many of the generated Bemis-Murcko scaffolds are in the ZINC 250k pre-training data. Table S28 reports the metrics for the Saturn configurations with and without the Tanimoto objective, averaged across all 10 seeds.

5.7 Saturn Framework: Architecture Scaling

In the main text Part 1, we investigated *why* Mamba (5.2M) outperforms LSTM [50] RNN (5.8M) and Decoder transformer [10, 11] (6.3M). Augmented Memory [1] squeezes the likelihood of generating augmented forms of *any* replay buffer *molecules*. Increased capacity to match this distribution directly leads to the "hop-and-locally-exploit" behavior which improves sample efficiency. We note that our observations are for optimization landscapes that are not *too rough* [54, 74]. It is difficult to know *a priori* the roughness of optimization and also whether the benefits of "hop-and-locally-exploit" behavior is beneficial in higher-fidelity oracle settings. We leave this for future work.

Based on these observations, we investigate scaling benefits for the LSTM RNN and Decoder transformer models. Increasing model size can lead to lower loss convergence, which in this case, means modeling the conditional token distribution of the SMILES [34]. One may argue that this is simply a hyperparameter tuning which we missed. However, the purpose of this work is in

the goal-directed learning setting where we want to *tune* the model's distribution towards desirable molecules. If desirable molecules are already in the training data, minimal optimization is required. Moreover, it is difficult to know *a priori* whether matching the training distribution *very closely* is strictly advantageous for an arbitrary MPO objective, unless we have an enormous amount of data, by the law of large numbers. Therefore, all pre-trained models (priors) in this work were trained until loss flattens out and Validity (fraction of valid SMILES generated) is high.

In this section, we scale up the LSTM RNN and Decoder transformer models to around 25M to make the *distribution learning capability* approach Mamba (5.2M). We use the training loss for this, where similar loss convergence is taken as the proxy. We first present the exact model parameter counts, hyperparameters, and training details.

LSTM RNN 24.7M:

1. Seed = 0
2. Parameters = 24,741,442
3. Vocabulary Size = 66
4. Embedding Dimension = 256
5. Hidden Dimension = 512
6. Number of Layers = 12
7. Dropout = 0.0
8. Layer Normalization = False
9. Train Epochs = 300
10. Batch Size = 512
11. Learning Rate = 0.0001
12. Final NLL Loss at Epoch 300 = 29.318

Decoder 25.3M:

1. Seed = 0
2. Parameters = 25,306,178
3. Vocabulary Size = 66
4. Embedding Dimension = 256
5. Hidden Dimension = 1024
6. Number of Layers = 32
7. Number of Heads = 16
8. Dropout = 0.0
9. Train Epochs = 100
10. Batch Size = 512
11. Learning Rate = 0.0001
12. Final NLL Loss at Epoch 100 = 26.963

In addition, we scale up Mamba to 16M and 21M and also present the exact model parameter counts, hyperparameters, and training details. For these two models, we intentionally train until the loss is at similar values (NLL = 26) which suggests both models have learned the training distribution to a similar extent. Optimization then starts from a similar distribution.

Mamba 15.8M:

1. Seed = 0
2. Parameters = 15,785,728
3. Vocabulary Size = 66
4. Embedding Dimension = 256
5. **Number of Layers = 36**

6. Use RMSNorm = True
7. Residual in fp32 = True
8. Fused AddNorm = True
9. Train Epochs = 100
10. Batch Size = 512
11. Learning Rate = 0.0001
12. Final NLL Loss at Epoch 92 = 26.003

Mamba 21.0M:

1. Seed = 0
2. Parameters = 21,041,920
3. Vocabulary Size = 66
4. Embedding Dimension = 256
5. **Number of Layers = 48**
6. Use RMSNorm = True
7. Residual in fp32 = True
8. Fused AddNorm = True
9. Train Epochs = 100
10. Batch Size = 512
11. Learning Rate = 0.0001
12. Final NLL Loss at Epoch 75 = 25.993

Table S29: Architecture scaling experiments: Hit Ratio (%) metrics. GEAM [43] and Saturn results are across 10 seeds (0-9 inclusive). The mean and standard deviation are reported.

Method	Target Protein				
	parp1	fa7	5ht1b	braf	jak2
**Datasets**					
ZINC 250k [49]	3.993 ± 0.355	1.097 ± 0.192	24.26 ± 0.622	1.020 ± 0.193	6.183 ± 0.344
ChEMBL 33 [48]	6.077 ± 0.453	1.830 ± 0.240	24.163 ± 0.715	2.073 ± 0.181	9.013 ± 0.562
**Generative Models**					
Augmented Memory [1]	16.983 ± 3.221	2.641 ± 0.868	52.046 ± 2.327	8.354 ± 1.727	21.604 ± 4.958
GEAM [43]	49.597 ± 3.078	21.988 ± 2.968	51.765 ± 1.463	33.086 ± 1.673	51.228 ± 3.132
**Ours**					
Saturn-Mamba 5.2M	57.981 ± 18.537	14.527 ± 9.961	68.185 ± 3.400	38.999 ± 10.114	60.827 ± 11.502
Saturn-Mamba 15.8M	56.088 ± 9.899	18.804 ± 13.980	68.322 ± 3.885	38.699 ± 19.841	61.320 ± 18.673
Saturn-Mamba 21.0M	56.299 ± 16.583	23.764 ± 19.280	65.015 ± 6.060	32.018 ± 12.584	59.175 ± 20.689
Saturn-Decoder 25.3M	61.732 ± 16.032	21.058 ± 13.940	68.340 ± 5.094	37.399 ± 12.632	65.470 ± 12.628
Saturn-RNN 24.7M	52.914 ± 9.955	13.254 ± 7.276	63.799 ± 3.249	33.805 ± 8.694	54.165 ± 7.445

Hit Ratios (%). Table S29 shows the Hit Ratios of compared models. Saturn outperforms baseline Augmented Memory and GEAM. In terms of architecture scaling, we show decoder transformer and RNN approach Mamba performance. Scaling up Mamba does not necessarily lead to better results, as there is notably even higher variance.

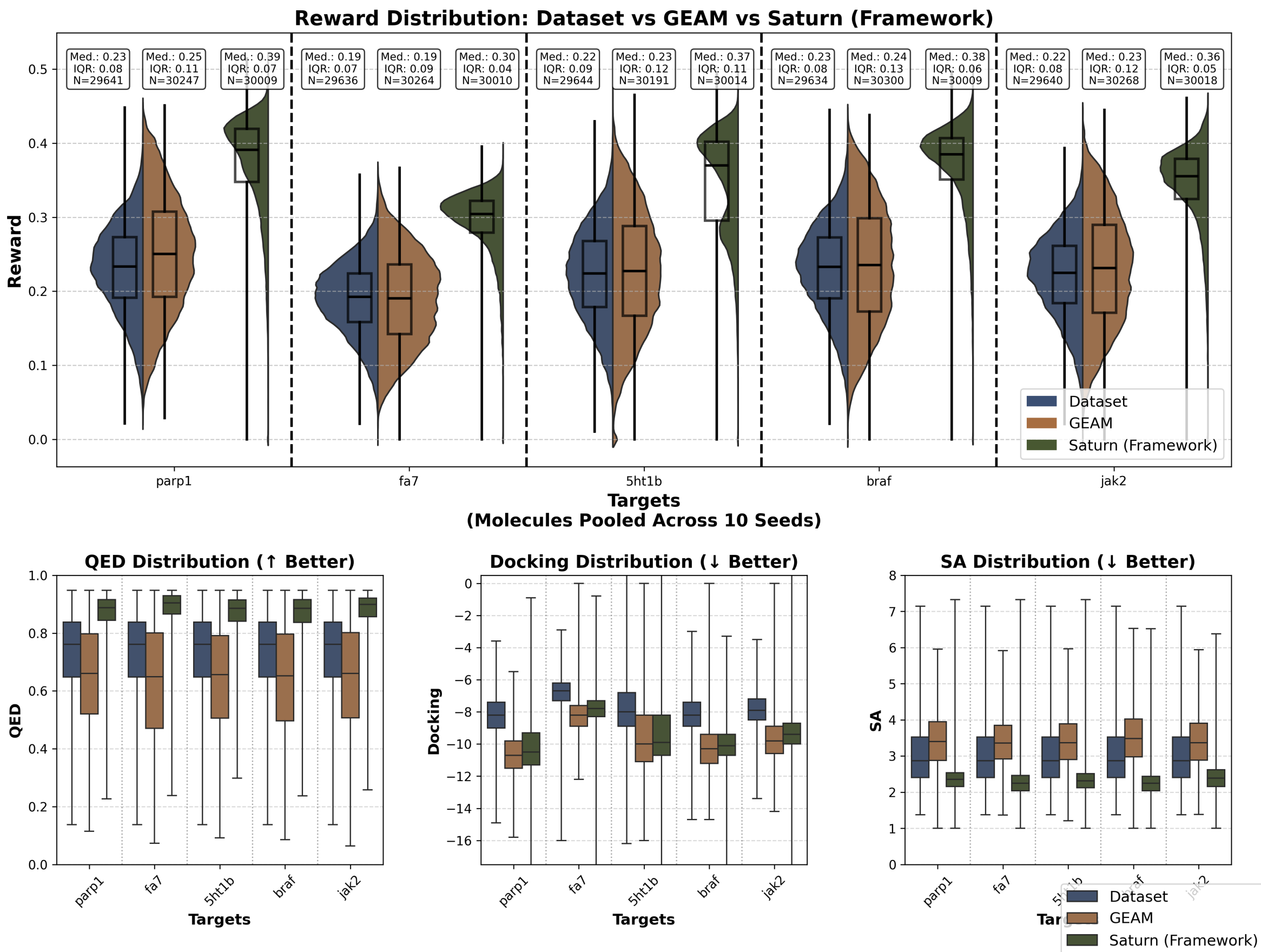

Figure S5: Reward distribution across all 5 protein targets for molecules randomly sampled from ZINC 250k, GEAM generated, and Saturn generated. The molecules are pooled across 10 seeds (0-9 inclusive) and molecules that failed to dock were removed. The below sub-plots show the distributions of the individual oracle components. The box-plot whiskers cover the full data range. The middle line is the median, and upper and lower lines denote the inter-quartile range.

Table S30: Architecture scaling experiments: Strict Hit Ratio (%) (QED > 0.7 and SA < 3). Saturn (Framework) results are across 10 seeds (0-9 inclusive). OB is Oracle Burden (oracle calls required to generate *N* unique molecules). The number in parentheses in the OB statistics represent how many runs out of 10 were successful. The mean and standard deviation are reported.

Method	Target Protein				
	parp1	fa7	5ht1b	braf	jak2
**Saturn-Mamba 5.2M**					
Strict Hit Ratio	55.102 ± 18.027	13.887 ± 9.723	64.730 ± 3.717	37.250 ± 9.615	55.903 ± 13.613
IntDiv1 (↑)	0.585 ± 0.049	0.583 ± 0.067	0.675 ± 0.021	0.587 ± 0.042	0.628 ± 0.036
#Circles (↑)	5 ± 1	3 ± 1	17 ± 3	4 ± 1	8 ± 2
OB (1) (↓)	$139 \pm 96(10)$	$352 \pm 206(10)$	$21 \pm 7(10)$	$291 \pm 143(10)$	$88 \pm 56(10)$
OB (10) (↓)	$518 \pm 92(10)$	$924 \pm 247(10)$	$105 \pm 23(10)$	$581 \pm 123(10)$	$348 \pm 96(10)$
OB (100) (↓)	$956 \pm 259(10)$	$1776 \pm 551(10)$	$441 \pm 44(10)$	$1057 \pm 187(10)$	$785 \pm 191(10)$
**Saturn-Mamba 15.8M**					
Strict Hit Ratio	52.093 ± 12.503	18.064 ± 13.932	63.740 ± 5.623	37.350 ± 19.173	59.372 ± 18.465
IntDiv1 (↑)	0.576 ± 0.033	0.577 ± 0.070	0.652 ± 0.044	0.557 ± 0.066	0.621 ± 0.035
#Circles (↑)	6 ± 2	4 ± 1	18 ± 3	4 ± 1	8 ± 2
OB (1) (↓)	$157 \pm 112(10)$	$223 \pm 167(10)$	$25 \pm 10(10)$	$204 \pm 115(10)$	$54 \pm 43(10)$
OB (10) (↓)	$406 \pm 111(10)$	$691 \pm 151(10)$	$108 \pm 31(10)$	$634 \pm 180(10)$	$266 \pm 50(10)$
OB (100) (↓)	$905 \pm 204(10)$	$1491 \pm 389(8)$	$421 \pm 61(10)$	$1220 \pm 410(10)$	$786 \pm 254(10)$
**Saturn-Mamba 21.0M**					
Strict Hit Ratio	54.297 ± 16.480	23.021 ± 19.064	61.307 ± 5.991	30.972 ± 12.605	57.013 ± 20.601
IntDiv1 (↑)	0.580 ± 0.041	0.523 ± 0.059	0.646 ± 0.044	0.548 ± 0.060	0.592 ± 0.047
#Circles (↑)	6 ± 1	3 ± 0	17 ± 3	4 ± 1	8 ± 2
OB (1) (↓)	$167 \pm 73(10)$	$316 \pm 236(10)$	$28 \pm 13(10)$	$235 \pm 138(10)$	$68 \pm 78(10)$
OB (10) (↓)	$425 \pm 91(10)$	$710 \pm 314(10)$	$115 \pm 44(10)$	$556 \pm 147(10)$	$335 \pm 118(10)$
OB (100) (↓)	$831 \pm 147(10)$	$1446 \pm 629(9)$	$432 \pm 69(10)$	$1134 \pm 282(10)$	$798 \pm 340(10)$
**Saturn-Decoder 25.3M**					
Strict Hit Ratio	59.560 ± 15.480	20.195 ± 13.394	65.202 ± 5.847	35.857 ± 12.228	62.874 ± 11.810
IntDiv1 (↑)	0.605 ± 0.034	0.565 ± 0.081	0.646 ± 0.032	0.605 ± 0.045	0.578 ± 0.062
#Circles (↑)	5 ± 1	3 ± 1	12 ± 4	3 ± 0	7 ± 1
OB (1) (↓)	$98 \pm 81(10)$	$242 \pm 160(10)$	$18 \pm 5(10)$	$248 \pm 81(10)$	$52 \pm 37(10)$
OB (10) (↓)	$375 \pm 131(10)$	$797 \pm 227(10)$	$92 \pm 29(10)$	$515 \pm 98(10)$	$320 \pm 63(10)$
OB (100) (↓)	$769 \pm 165(10)$	$1698 \pm 507(10)$	$378 \pm 43(10)$	$1101 \pm 216(10)$	$722 \pm 140(10)$
**Saturn-RNN 24.7M**					
Strict Hit Ratio	50.586 ± 9.574	12.731 ± 7.211	60.331 ± 3.294	32.380 ± 8.503	51.819 ± 7.247
IntDiv1 (↑)	0.644 ± 0.023	0.634 ± 0.040	0.711 ± 0.018	0.628 ± 0.030	0.681 ± 0.026
#Circles (↑)	8 ± 2	4 ± 1	25 ± 5	6 ± 1	13 ± 2
OB (1) (↓)	$126 \pm 99(10)$	$384 \pm 289(10)$	$27 \pm 19(10)$	$186 \pm 170(10)$	$50 \pm 52(10)$
OB (10) (↓)	$465 \pm 71(10)$	$1243 \pm 273(10)$	$111 \pm 41(10)$	$714 \pm 214(10)$	$305 \pm 100(10)$
OB (100) (↓)	$1045 \pm 148(10)$	$2150 \pm 311(10)$	$487 \pm 61(10)$	$1404 \pm 269(10)$	$935 \pm 130(10)$

Sample Efficiency Metrics. Table S30 presents the Strict Hit Ratios for compared models. While GEAM outperforms baseline Augmented Memory for the Hit Ratio, the results here show that the optimization capability of baseline Augmented Memory exceeds that of GEAM. Saturn outperforms both Augmented Memory and GEAM to generate more hits and also finds them faster (lower OB). Next, we investigate architecture scaling again, but this time, under the strict filter. Decoder transformer (25.3M) approaches Mamba (5.2M) performance and outperforms it in many tasks (Figure S30), trading off even more diversity. Variance is also higher. However, we believe this is an interesting observation as Augmented Memory's mechanism is squeezing the likelihood of augmented sequences. By simply scaling up the architecture and enabling the model to converge to this distribution, sample efficiency improves. Finally, while scaling up the architecture to the parameter counts we have investigated adds negligible generation time, Mamba (5.2M) is *parameter-efficient* in its ability to intensify the behavior of Augmented Memory.

5.8 Qualitative Supplementary Results

In this section, we show random generated molecules from Saturn that pass the Strict Filter (Figure S6). All molecules possess QuickVina 2 [53] docking scores better than the median of known actives [65] while possessing QED [31] > 0.7 and SA score [32] < 3. We further highlight two points: firstly, there may be some particularly large rings that are undesirable from a chemistry perspective, even though QED and SA score permits them. Saturn is an optimization engine and if specific chemistry is desired, including it into the MPO objective will steer the Agent away from this chemical space. In this work, a concrete example of this is in the main text Part 2 experiments where the Saturn pre-trained model was additionally pre-trained via curriculum learning [74] to generate molecules dissimilar to the ZINC 250k [49] training data to satisfy the *Novel* metric defined Lee et al [43, 65]. This example shows the flexibility of Saturn. Secondly, as stereochemistry was not purged from the vocabulary, Saturn can generate stereoisomers.

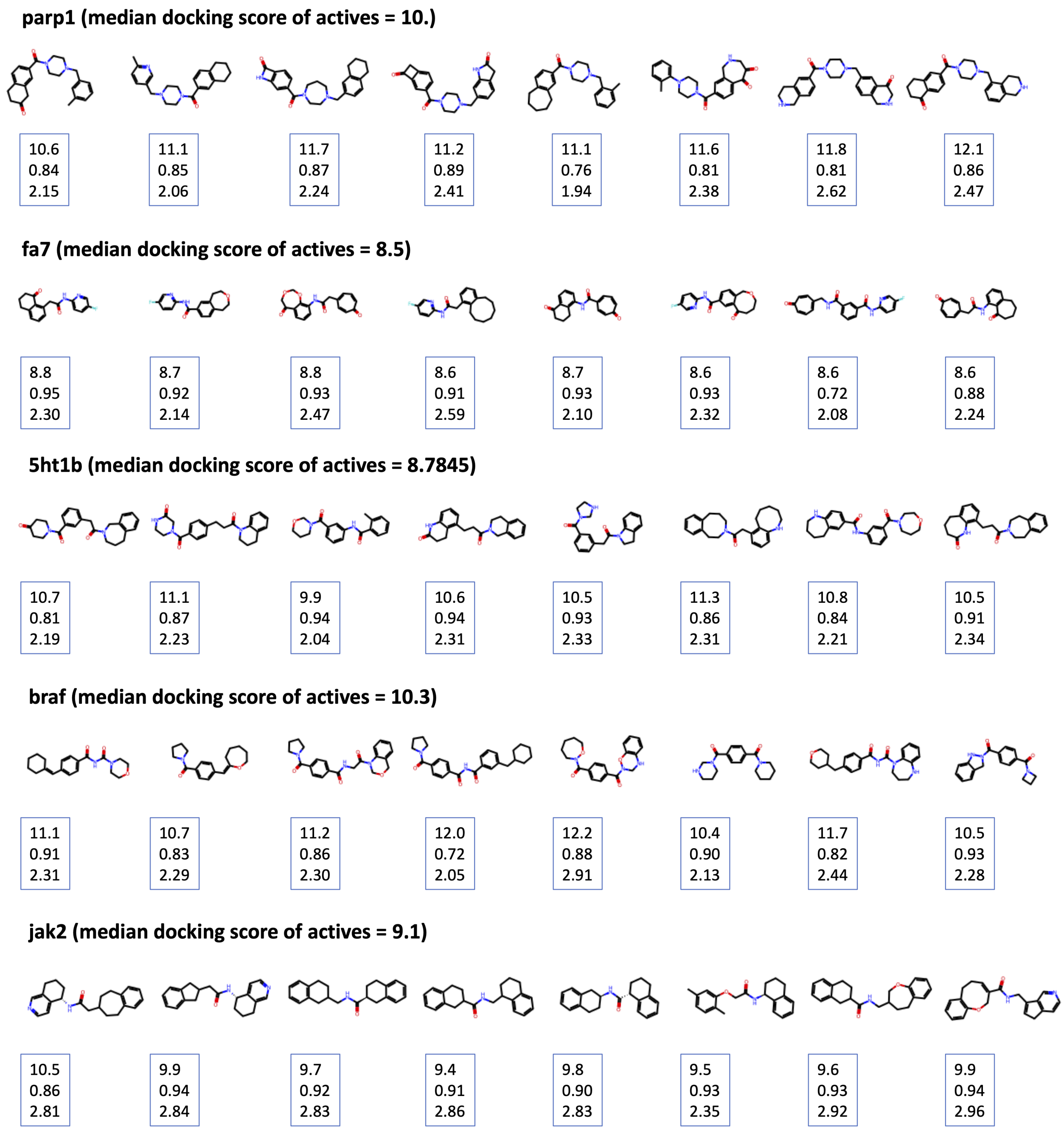

Figure S6: Example Saturn generated molecules passing the Strict Filter for all 5 targets: parp1, fa7, 5ht1b, braf, and jak2. The scores are annotated from top to bottom, QuickVina 2 [53] docking score, QED [31], and SA score [32].

6 Part 3: Directly Optimizing Electronic Properties at the DFT Fidelity-level

In this section, we provide details on the density function theory (DFT) simulations used for the main text Part 3 experiments and additional details on Saturn.

6.1 Details On The DFT Simulations To Compute The HOMO-LUMO Gap

The workflow for calculating the HOMO–LUMO gap from the SMILES representation of generated molecules consists of four main steps: (1) generating a set of initial molecular conformations, (2) performing preliminary geometry optimization using a semi-empirical quantum mechanical method and keeping the lowest energy one, (3) optimizing the molecular geometry with density functional theory (DFT), and (4) conducting single-point energy calculations with DFT to obtain the HOMO–LUMO gap. Specifically, RDKit [75] is employed to randomly generate 25 initial 3D conformations from each SMILES string. Subsequently, all conformations are optimized using the MOPAC (version 22.1.1) software [76] with the PM6-D3H4 method [77], and the lowest-energy structure is selected. Finally, DFT is applied for both geometry optimization and single-point energy calculations. DFT simulations were carried out with Orca versions 6.0.0 [78] using B3LYP functional [79–81], def2-TZVP [82] basis set and default TightSCF and geometry convergence criteria. The resolution of identity (RI) approximation was used for pure functionals and Orca's RIJCOSX for hybrid functionals. Dispersion corrections were added via Grimme's D3 model with Becke and Johnson damping [83].

6.2 Potential Challenges when Pushing Towards Other High-fidelity Oracles

Throughout the main text and Supplementary Information, we have made an effort to demonstrate Saturn's broad applicability. However, it remains to be seen whether performance will carry over to other high-fidelity oracles with rougher optimization landscapes [54], where the "hop-and-locally-exploit" behavior may be disadvantageous. However, as we have identified *why* this behavior manifests, we can tailor the sampling behavior for the optimization landscape, if required. For

example, activating the genetic algorithm and lowering augmentation rounds loosens the local sampling behavior, as shown in Supplementary Information 3.2.

6.3 Saturn Framework: Details Specific to Running DFT

Saturn continues to use the default hyperparameters in this work: batch size 16 and 10 augmentation rounds. DFT was performed by parallelizing each generated molecule on 72 CPU cores using 2 Intel(R) Xeon(R) Platinum 8360Y processors with a maximum time limit per molecule to 1 hour. If a molecule went over this time limit, a default, near-zero reward is assigned. Since the original release of Saturn's code, we noticed that repeated zero-reward molecules sometimes led to Agent drift such that no valid SMILES are generated. This has been previously observed [84] and could be rectified by resetting to a previous checkpoint. We also observed that the default (in this work) AdamW [**?**] optimizer is more prone to this validity drift than Adam [86]. For this reason, we used Adam for the DFT experiments. Lastly, due to the syntax of how SMILES are generated, radical molecules *can* be generated, even though the pre-training data contains no radicals. Since DFT can be extremely sensitive to radicals, we added a check to remove radicals should they ever be generated. Note that generating radicals is a rare occurrence and we show an example of how the SMILES syntax can cause this: "C1CCCCC1" encodes cyclohexane. By chance, if one of the carbons before closing the ring were a "stereocenter carbon" such as "C1CCC[C@@H]C1", this will map to a radical carbon. Note that a stereo-carbon cannot actually exist at that position because the carbon is not bonded to four different groups.

6.4 Novelty Metrics

Table S31: Molecule and Bemis-Murcko scaffold novelty metrics across 3 seeds in the DFT experiments. The pre-training data was ChEMBL 33.

	Without SA	With SA
Molecule Novelty	0.975 ± 0.010	0.958 ± 0.019
Scaffold Novelty	0.646 ± 0.025	0.533 ± 0.104

In this subsection, we report the molecule novelty: how many of the generated molecules are

in the ChEMBL 33 [48] pre-training data and the scaffold novelty: how many of the generated Bemis-Murcko scaffolds are in the ChEMBL 33 pre-training data. Table S31 reports the metrics for the optimization objectives with and without SA score, averaged across all 3 seeds.

References

[1] Guo, J. & Schwaller, P. Augmented Memory: Sample-Efficient Generative Molecular Design with Reinforcement Learning. *JACS Au* **4**, 2160–2172 (2024).

[2] Guo, J. & Schwaller, P. Beam Enumeration: Probabilistic Explainability For Sample Efficient Self-conditioned Molecular Design. *Proc. 12th International Conference on Learning Representations* (2024).

[3] Olivecrona, M. *et al.* Molecular de-novo design through deep reinforcement learning. *Journal of Cheminformatics* **9**, (2017).

[4] Blaschke, T. *et al.* REINVENT 2.0: An AI Tool for De Novo Drug Design. *Journal of Chemical Information and Modeling* **60**, 5918–5922 (2020).

[5] Loeffler, H. H *et al.* Reinvent 4: Modern AI–driven generative molecule design. *Journal of Cheminformatics* **16**, (2024).

[6] Fialková, V. *et al.* LibINVENT: reaction-based generative scaffold decoration for in silico library design. *Journal of Chemical Information and Modeling* **62**, 2046–2063 (2021).

[7] Guo, J., Knuth, F. *et al.* Link-INVENT: generative linker design with reinforcement learning. *Digital Discovery* **2**, 392–408 (2023).

[8] He, J. *et al.* Molecular optimization by capturing chemist's intuition using deep neural networks. *Journal of Cheminformatics* **13**, (2021).

[9] Tibo, A. *et al.* Exhaustive local chemical space exploration using a transformer model. *Nature Communications* **15**, (2024).

[10] Vaswani, A. & Shazeer, N. & Parmar, N. & Uszkoreit, J. & Jones, L. & Gomez, A. N & Kaiser, L. & Polosukhin, I. Attention Is All You Need. *Proc. 31st Neural Information Processing Systems* (2017).

[11] Radford, A. & Wu, J. *et al.* Language Models are Unsupervised Multitask Learners. *OpenAI Technical Report* (2019).

[12] Gu, A. & Dao, T. Mamba: Linear-Time Sequence Modeling with Selective State Spaces. *Proc. 1st Conference on Language Modeling* (2024).

[13] Jensen, J. H A graph-based genetic algorithm and generative model/Monte Carlo tree search for the exploration of chemical space. *Chemical Science* **10**, 3567–3572 (2019).

[14] Kim, H. *et al.* Genetic-guided GFlowNets for Sample Efficient Molecular Optimization. *Proc. 38th Neural Information Processing Systems* (2024).

[15] Malkin, N. *et al.* Trajectory balance: Improved credit assignment in gflownets. *Proc. 36th Neural Information Processing Systems* (2022).

[16] Blaschke, T. *et al.* Memory-assisted reinforcement learning for diverse molecular de novo design. *Journal of Cheminformatics* **12**, (2020).

[17] Bjerrum, E. J. & Threlfall, R. Molecular Generation with Recurrent Neural Networks (RNNs). *arXiv preprint arXiv:1705.04612* (2017).

[18] Segler, M. H. *et al.* Generating focused molecule libraries for drug discovery with recurrent neural networks. *ACS Central Science* **4**, 120–131 (2017).

[19] Popova, M. *et al.* Deep reinforcement learning for de novo drug design. *Science Advances* **4**, (2018).

[20] Brown, N. *et al.* GuacaMol: Benchmarking Models for de Novo Molecular Design. *Journal of Chemical Information and Modeling* **59**, 1096–1108 (2019).

[21] Polykovskiy, D. *et al.* Molecular Sets (MOSES): A Benchmarking Platform for Molecular Generation Models. *Frontiers in Pharmacology* **11**, (2020).

[22] Flam-Shepherd, A. *et al.* Language models can learn complex molecular distributions. *Nature Communications* **13**, (2022).

[23] Bagal, V. *et al.* MolGPT: Molecular Generation Using a Transformer-Decoder Model. *Journal of Chemical Information and Modeling* **62**, 2064–2076 (2021).

[24] Wang, Y. *et al.* cMolGPT: A Conditional Generative Pre-Trained Transformer for Target-Specific De Novo Molecular Generation. *Molecules* **28**, (2023).

[25] Xu, P., Feng, T. *et al.* Molecular De Novo Design through Transformer-based Reinforcement Learning. *arXiv preprint arXiv:2310.05365* (2024).

[26] Mazuz, E. *et al.* Molecule generation using transformers and policy gradient reinforcement learning. *Scientific Reports* **13**, (2023).

[27] Yang, S. *et al.* De novo Drug Design using Reinforcement Learning with Multiple GPT Agents. *Proc. 37th Neural Information Processing Systems* (2023).

[28] He, J. *et al.* Evaluation of reinforcement learning in transformer-based molecular design. *Journal of Cheminformatics* **16**, (2024).

[29] Thomas, M. *et al.* Augmented Hill-Climb increases reinforcement learning efficiency for language-based de novo molecule generation. *Journal of Cheminformatics* **14**, (2022).

[30] Yang, Y., Zheng, S. *et al.* SyntaLinker: automatic fragment linking with deep conditional transformer neural networks. *Chemical Science* **11**, 8312–8322 (2020).

[31] Bickerton, G. R. *et al.* Quantifying the chemical beauty of drugs. *Nature chemistry* **4**, 90–98 (2012).

[32] Ertl, P. & Schuffenhauer, A. Estimation of synthetic accessibility score of drug-like molecules based on molecular complexity and fragment contributions. *Journal of Cheminformatics* **1**, (2009).

[33] Özçelik, R. *et al.* Chemical language modeling with structured state space sequence models. *Nature Communications* **15**, (2024).

[34] Weininger, D. SMILES, a chemical language and information system. 1. Introduction to methodology and encoding rules. *Journal of Chemical Information and Modeling* **28**, 31–36 (1988).

[35] Gu, A. *et al.* Efficiently modeling long sequences with structured state spaces. *Proc. 10th International Conference on Learning Representations* (2022).

[36] Chen, J. Mamba No. 5 (A Little Bit Of...). (2024).

[37] Gu, A. *et al.* Combining recurrent, convolutional, and continuous-time models with linear state space layers. *Proc. 35th Neural Information Processing Systems* (2021).

[38] Gu, A. Modeling sequences with structured state spaces. (2023).

[39] Bjerrum, E. J. SMILES enumeration as data augmentation for neural network modeling of molecules. *arXiv preprint arXiv:1703.07076* (2017).

[40] Gao, W., Fu, T. *et al.* Sample Efficiency Matters: A Benchmark for Practical Molecular Optimization. *Proc. 36th Neural Information Processing Systems Track on Datasets and Benchmarks* (2022).

[41] Tripp, A. & Hernández-Lobato, J. M. Genetic algorithms are strong baselines for molecule generation. *arXiv preprint arXiv:1703.07076* (2023).

[42] Liu, X. *et al.* DrugEx v2: de novo design of drug molecules by Pareto-based multi-objective reinforcement learning in polypharmacology. *Journal of Cheminformatics* **13**, (2021).

[43] Lee, S. *et al.* Drug Discovery with Dynamic Goal-aware Fragments. *Proc. 41st International Conference on Machine Learning* (2024).

[44] Eckmann, P. *et al.* MF-LAL: Drug Compound Generation Using Multi-Fidelity Latent Space Active Learning. *Proc. 42st International Conference on Machine Learning*, (2025).

[45] Crivelli-Decker, J. E. *et al.* Machine Learning Guided AQFEP: A Fast and Efficient Absolute Free Energy Perturbation Solution for Virtual Screening. *Journal of Chemical Theory and Computation* **20**, 7188–7198 (2024).

[46] Schlegel, M. *et al.* Importance resampling for off-policy prediction. *Proc. 33rd Neural Information Processing Systems* (2019).

[47] Williams, R. J. Simple statistical gradient-following algorithms for connectionist reinforcement learning. *Machine Learning* **8**, 229–256 (1992).

[48] Gaulton, A. *et al.* ChEMBL: a large-scale bioactivity database for drug discovery. *Nucleic Acids Research* **40**, D1100–D1107 (2012).

[49] Sterling, T. & Irwin, J. J. Zinc 15–ligand discovery for everyone. *Journal of Chemical Information and Modeling* **55**, 2324–2337 (2015).

[50] Hochreiter, S. & Schmidhuber, J. Long short-term memory. *Neural Computation* **9**, 1735–1780 (1997).

[51] Benhenda, M. ChemGAN challenge for drug discovery: can AI reproduce natural chemical diversity?. *arXiv* (2017).

[52] Trott, O. & Olson, A. J. AutoDock Vina: improving the speed and accuracy of docking with a new scoring function, efficient optimization, and multithreading. *Journal of Computational Chemistry* **31**, 455–461 (2010).

[53] Alhossary, A. *et al.* Fast, accurate, and reliable molecular docking with quickvina 2. *Bioinformatics* **31**, 2214–2216 (2015).

[54] Aldeghi, M. *et al.* Zinc 15–ligand discovery for everyone. *Journal of Chemical Information and Modeling* **62**, 4660–4671 (2022).

[55] Wang, S. *et al.* Structure of the D2 dopamine receptor bound to the atypical antipsychotic drug risperidone. *Nature* **555**, 269–273 (2018).

[56] Kryger, G. *et al.* Structure of acetylcholinesterase complexed with E2020 (Aricept®): implications for the design of new anti-Alzheimer drugs. *Structure* **7**, 297–307 (1999).

[57] Argiriadi, M. A. *et al.* 2, 4-Diaminopyrimidine MK2 inhibitors. Part I: observation of an unexpected inhibitor binding mode. *Bioorganic & Medicinal Chemistry Letters* **20**, 330–333 (2010).

[58] Bemis, G. W. & Murcko, M. A. The Properties of Known Drugs. 1. Molecular Frameworks. *Journal of Medicinal Chemistry* **39**, 2887–2893 (1996).

[59] Sastry, G. M. *et al.* Protein and ligand preparation: parameters, protocols, and influence on virtual screening enrichments. *Journal of Computer-aided Molecular Design* **27**, 221–234 (2013).

[60] Schrödinger Release 2019-4: Protein Preparation Wizard; Epik, Schrödinger, LLC, New York, NY, 2019; Impact, Schrödinger, LLC, New York, NY; Prime, Schrödinger, LLC, New York, NY, 2019.

[61] Roos, K. *et al.* OPLS3e: Extending force field coverage for drug-like small molecules. *Journal of Chemical Theory and Computation* **15**, 1863–1874 (2019).

[62] Guo, J. *et al.* DockStream: a docking wrapper to enhance de novo molecular design. *Journal of Cheminformatics* **13**, (2021).

[63] Rappé, A. K. *et al.* UFF, a full periodic table force field for molecular mechanics and molecular dynamics simulations. *Journal of the American Chemical Society* **114**, 10024–10035 (1992).

[64] Xie, Y. *et al.* How Much Space Has Been Explored? Measuring the Chemical Space Covered by Databases and Machine-Generated Molecules. *Proc. 11th International Conference on Learning Representations* (2023).

[65] Lee, S. *et al.* Exploring chemical space with score-based out-of-distribution generation. *Proc. 40th International Conference on Machine Learning* (2023).

[66] Jin, W., *et al.* Junction Tree Variational Autoencoder for Molecular Graph Generation. *Proc. 35th International Conference on Machine Learning* (2018).

[67] Shi, C., Xu, M. *et al.* Graphaf: a flow-based autoregressive model for molecular graph generation. *Proc. 8th International Conference on Learning Representations* (2020).

[68] Jeon, W. & Kim, D. Autonomous molecule generation using reinforcement learning and docking to develop potential novel inhibitors. *Scientific Reports* **10**, (2020).

[69] Jin, W. *et al.* Hierarchical generation of molecular graphs using structural motifs. *Proc. 37th International Conference on Machine Learning* (2020).

[70] Luo, Y. *et al.* Graphdf: A discrete flow model for molecular graph generation. *Proc. 38th International Conference on Machine Learning* (2021).

[71] Yang, S. *et al.* Hit and lead discovery with explorative rl and fragment-based molecule generation. *Proc. 35th Neural Information Processing Systems* (2021).

[72] Eckmann, P. *et al.* Limo: Latent inceptionism for targeted molecule generation. *Proc. 39th International Conference on Machine Learning* (2022).

[73] Jo, J., Lee, S., Hwang, S. J. Score-based generative modeling of graphs via the system of stochastic differential equations. *Proc. 39th International Conference on Machine Learning* (2022).

[74] Skinnider, M. A. *et al.* Chemical language models enable navigation in sparsely populated chemical space. *Nature Machine Intelligence* **3**, 759–770 (2021).

[75] RDKit: Open-source cheminformatics. https://www.rdkit.org.

[76] Stewart, J. JP MOPAC: a semiempirical molecular orbital program. *Journal of Computer-aided Molecular Design* **4**, 1–103 (1990).

[77] Rezac, J. & Hobza, P. Advanced corrections of hydrogen bonding and dispersion for semiempirical quantum mechanical methods. *Journal of Chemical Theory and Computation* **8**, 141–151 (2012).

[78] Neese, F. The ORCA program system. *Wiley Interdisciplinary Reviews: Computational Molecular Science* **2**, 73–78 (2012).

[79] Becke, A. D. Density-functional exchange-energy approximation with correct asymptotic behavior. *Physical Review A* **38**, 3098–3100 (1988).

[80] Becke, A. D. A new mixing of Hartree-Fock and local densityfunctional theories. *The Journal of Chemical Physics* **98**, 1372–1377 (1993).

[81] Lee, C. *et al.* Development of the Colle-Salvetti correlation-energy formula into a functional of the electron density. *Physical Review B* **37**, 785–789 (1988).

[82] Weigend, F. & Ahlrichs, R. Balanced basis sets of split valence, triple zeta valence and quadruple zeta valence quality for H to Rn: Design and assessment of accuracy. *Physical Chemistry Chemical Physics* **7**, 3297–3305 (2005).

[83] Weigend, F. & Ahlrichs, R. A consistent and accurate ab initio parametrization of density functional dispersion correction (DFT-D) for the 94 elements H-Pu. *The Journal of Chemical Physics* **132**, (2010).

[84] Svensson, H. G. *et al.* Utilizing reinforcement learning for de novo drug design. *Machine Learning* **113**, 4811–4843 (2024).

[85] Loshchilov, I. & Hutter, F. Adam: A Method for Stochastic Optimization. *Proc. 3rd International Conference on Learning Representations* (2015).

[86] Kingma, D. P. & Ba, J. Decoupled Weight Decay Regularization. *Proc. 7th International Conference on Learning Representations* (2019).
