# Supplementary material for: Sample-efficient generative molecular design using memory manipulation
Source: Nat Mach Intell. 2026 Mar 17;8(3):449–60. doi: 10.1038/s42256-026-01200-4 (PMC13658037; doi:10.1038/s42256-026-01200-4)
Supplement: Supplementary file 2 [file 42256_2026_1200_MOESM2_ESM.pdf]

nature portfolio

Corresponding author(s): Jeff Guo, Philippe Schwaller

Last updated by author(s): 2025/12/29

Reporting Summary

Nature Portfolio wishes to improve the reproducibility of the work that we publish. This form provides structure for consistency and transparency in reporting. For further information on Nature Portfolio policies, see our Editorial Policies and the Editorial Policy Checklist.

Statistics

For all statistical analyses, confirm that the following items are present in the figure legend, table legend, main text, or Methods section.

n/a	Confirmed	
☐	☒	The exact sample size (*n*) for each experimental group/condition, given as a discrete number and unit of measurement
☐	☒	A statement on whether measurements were taken from distinct samples or whether the same sample was measured repeatedly
☐	☒	The statistical test(s) used AND whether they are one- or two-sided<br>*Only common tests should be described solely by name; describe more complex techniques in the Methods section.*
☒	☐	A description of all covariates tested
☒	☐	A description of any assumptions or corrections, such as tests of normality and adjustment for multiple comparisons
☐	☒	A full description of the statistical parameters including central tendency (e.g. means) or other basic estimates (e.g. regression coefficient) AND variation (e.g. standard deviation) or associated estimates of uncertainty (e.g. confidence intervals)
☐	☒	For null hypothesis testing, the test statistic (e.g. *F*, *t*, *r*) with confidence intervals, effect sizes, degrees of freedom and *P* value noted<br>*Give P values as exact values whenever suitable.*
☒	☐	For Bayesian analysis, information on the choice of priors and Markov chain Monte Carlo settings
☒	☐	For hierarchical and complex designs, identification of the appropriate level for tests and full reporting of outcomes
☒	☐	Estimates of effect sizes (e.g. Cohen's *d*, Pearson's *r*), indicating how they were calculated

Our web collection on statistics for biologists contains articles on many of the points above.

Software and code

Policy information about availability of computer code

Data collection	All code is open sourced and provided here: https://github.com/schwallergroup/saturn<br>In the code base, details on other open source software used is provided here: https://github.com/schwallergroup/saturn/tree/master/experimental_reproduction.
Data analysis	The benchmark data was analyzed using python and is partly based on the following code: https://github.com/SeulLee05/GEAM/blob/main/eval.py from the GEAM model which we benchmarked against and follow their metrics. No other tools or commercial software were used.

For manuscripts utilizing custom algorithms or software that are central to the research but not yet described in published literature, software must be made available to editors and reviewers. We strongly encourage code deposition in a community repository (e.g. GitHub). See the Nature Portfolio guidelines for submitting code & software for further information.

Data

Policy information about availability of data

All manuscripts must include a data availability statement. This statement should provide the following information, where applicable:

- Accession codes, unique identifiers, or web links for publicly available datasets
- A description of any restrictions on data availability
- For clinical datasets or third party data, please ensure that the statement adheres to our policy

The datasets used for pre-training are ChEMBL 33 and ZINC 250k, which can be freely downloaded from https://ftp.ebi.ac.uk/pub/databases/chembl/ChEMBLdb/

releases/chembl_33/ and https://github.com/SeulLee05/GEAM/tree/main/data, respectively. The instructions for processing these datasets are provided in the Supplementary Section 1.6. Additionally, the pre-trained models are provided in the code repository: https://github.com/schwallergroup/saturn/tree/master/experimental_reproduction/checkpoint_models. The archived codebase version used in this work is deposited here: https://doi.org/10.6084/m9.figshare.30968380

Research involving human participants, their data, or biological material

Policy information about studies with human participants or human data. See also policy information about sex, gender (identity/presentation), and sexual orientation and race, ethnicity and racism.

Reporting on sex and gender	N/A
Reporting on race, ethnicity, or other socially relevant groupings	N/A
Population characteristics	N/A
Recruitment	N/A
Ethics oversight	N/A

Note that full information on the approval of the study protocol must also be provided in the manuscript.

Field-specific reporting

Please select the one below that is the best fit for your research. If you are not sure, read the appropriate sections before making your selection.

☒ Life sciences ☐ Behavioural & social sciences ☐ Ecological, evolutionary & environmental sciences

For a reference copy of the document with all sections, see nature.com/documents/nr-reporting-summary-flat.pdf

Life sciences study design

All studies must disclose on these points even when the disclosure is negative.

Sample size	In our computational study, we set an oracle (computational simulation) budget to cap the number of property evaluations we allow when developing and benchmarking our method. The sample sizes used in this paper were 1,000 (as a strict budget, as a popular benchmark on efficiency uses 10,000: https://arxiv.org/abs/2206.12411) and 3,000 which was used to enable an exact comparison with a recent method that achieves strong performance (https://icml.cc/virtual/2024/poster/32688).
Data exclusions	No data was excluded.
Replication	Every experiment in the paper was run for 10 replicates. The mean and standard deviation across all 10 replicates is always reported in the paper. The only exception to running 10 replicates is for the DFT experiment which was run for 3 replicates to keep computational costs manageable for us.
Randomization	N/A
Blinding	N/A

Reporting for specific materials, systems and methods

We require information from authors about some types of materials, experimental systems and methods used in many studies. Here, indicate whether each material, system or method listed is relevant to your study. If you are not sure if a list item applies to your research, read the appropriate section before selecting a response.

Materials & experimental systems

n/a	Involved in the study	
☒	☐	Antibodies
☒	☐	Eukaryotic cell lines
☒	☐	Palaeontology and archaeology
☒	☐	Animals and other organisms
☒	☐	Clinical data
☒	☐	Dual use research of concern
☒	☐	Plants

Methods

n/a	Involved in the study	
☒	☐	ChIP-seq
☒	☐	Flow cytometry
☒	☐	MRI-based neuroimaging

Plants

Seed stocks	N/A
Novel plant genotypes	N/A
Authentication	N/A
